# CG14075/Mmt promotes amino acid catabolism and cooperates with AKH during starvation in *Drosophila*

**DOI:** 10.1038/s41467-026-77687-7

**Published:** 2026-09-12

**Authors:** Yuto Yoshinari, Maiko Abe, Ryo Hoshino, Yosuke Mizuno, Yoshitomo Kurogi, Eisuke Imura, Akira Nakamura, Fuminori Tsuruta, Ryusuke Niwa, Takashi Nishimura

**Affiliations:** 1 Metabolic Regulation and Genetics, Institute for Molecular and Cellular Regulation, Gunma University, Maebashi, Japan; 2 Life Science Center for Survival Dynamics, Tsukuba Advanced Research Alliance (TARA), University of Tsukuba, Tsukuba, Japan; 3 Graduate School of Life and Environmental Sciences, University of Tsukuba, Tsukuba, Japan; 4 Department of Germline Development, Institute of Molecular Embryology and Genetics, Kumamoto University, Kumamoto, Japan; 5 Graduate School of Pharmaceutical Sciences, Kumamoto University, Kumamoto, Japan; 6 Institute of Life and Environmental Sciences, University of Tsukuba, Tsukuba, Japan; 7 Present Address: Department of Entomology, Institute for Integrative Genome Biology, University of California, Riverside, CA USA

**Keywords:** Homeostasis, Fat metabolism, Metabolomics, Metabolism, Feeding behaviour

## Abstract

Starvation triggers the sequential mobilization of glycogen, lipids, and amino acids to sustain survival, yet the hormonal mechanisms coordinating these transitions remain poorly understood. Here, we identify CG14075/Marmite (Mmt) as a hormone-like peptide secreted from the corpora cardiaca (CC) of *Drosophila melanogaster* during prolonged nutrient deprivation. CG14075/Mmt is progressively released into the haemolymph during starvation, coinciding with the decline of glycogen and lipid stores. CG14075/Mmt contributes to the regulation of amino acid catabolism, with prominent effects on threonine and branched-chain amino acid metabolism, and its absence leads to amino acid accumulation associated with oxidative stress. Moreover, CG14075/Mmt functions alongside adipokinetic hormone (AKH), the lipid-catabolic glucagon-like hormone, to regulate lipid and amino acid metabolism during starvation. Loss of CG14075/Mmt accelerates lipid and glycogen consumption and reduces starvation resistance, phenotypes that are partly associated with increased AKH secretion. These findings reveal a CC-derived endocrine component that contributes to the coordinated utilization of multiple energy substrates during nutrient stress.

## Abstract (web summary)

How animals survive without food depends on more than stored fat and sugar. This study shows that the fruit fly hormone CG14075/Marmite helps redirect amino acid use during starvation, working with a key hormone, AKH, to sustain energy balance.

## Introduction

Organisms adapt to starvation by mobilizing internal nutrient reserves to sustain survival. The metabolic response to starvation represents a fundamental physiological adaptation, and in many species, including humans, evolutionary pressures are thought to have selected for traits that enhance survival under nutrient-scarce conditions^1,2^. In mammals, metabolic flexibility enables a coordinated shift in energy sources from glycogen to lipids and eventually to amino acids during starvation. In humans, glycogenolysis occurs in the liver and muscle during early fasting^3^. As fasting progresses and glycogen stores decline, the body increasingly relies on fatty acid oxidation, while amino acids contribute to gluconeogenesis and serve as anaplerotic substrates to replenish tricarboxylic acid (TCA) cycle intermediates^4,5^. As starvation progresses, amino acid catabolism becomes increasingly important, not only as a source of glucose precursors but also as an anaplerotic input that replenishes TCA cycle intermediates and supports efficient energy production from lipid oxidation^6,7^. These metabolic transitions require coordinated inter-organ communication involving the central nervous system, liver, adipose tissue, and skeletal muscle. While hormonal signalling through insulin and glucagon is well established in regulating gluconeogenesis^8–10^, the mechanisms that drive the transition toward increased reliance on amino acid-derived fuels, and the metabolic reprogramming required for their efficient utilization during prolonged starvation, remain incompletely understood.

Similar to mammals, the fruit fly *Drosophila melanogaster* stores glycogen and lipids as energy reserves to cope with starvation. *Drosophila* insulin-like peptides (Dilps) induce anabolic processes in peripheral tissues, including the fat body and muscle^11^. During starvation, decreased haemolymph glucose levels trigger glycogenolysis to maintain glucose homeostasis^12^. As glycogen stores become depleted, energy metabolism shifts toward lipid mobilization. Lipolysis is regulated by the interplay between insulin signalling and adipokinetic hormone (AKH), a peptide hormone secreted from the neuroendocrine organ known as the corpora cardiaca (CC)^13–16^. Functionally analogous to mammalian glucagon, AKH promotes catabolic responses during starvation by acting through the Akh receptor (AkhR) in the fat body, thereby regulating the activity of triglyceride lipase(s)^13^. Prolonged starvation triggers autophagy in the fat body and ovaries, leading to the release of free amino acids that can be used as energy substrates^17,18^. Despite extensive studies on the hormonal regulation of carbohydrate and lipid metabolism in *Drosophila*, the endocrine mechanisms that drive amino acid catabolism and coordinate metabolic adaptation during prolonged starvation remain to be fully elucidated.

The activity of insulin-producing cells (IPCs) is stimulated by circulating sugars and amino acids, as well as by signals from other neurons and hormones released from the gut and fat body^19–23^. Conversely, CC cells are activated under nutrient-deprived conditions, and their activity is modulated by dietary sugar levels and enteroendocrine signals from the gut^19,21,24–26^. Anatomically, CC cells are located outside the brain and form a neuroendocrine ganglion attached to the foregut and midgut^14,16^. In many insects, the CC is regarded as a neurohemal organ that both synthesizes and stores neuropeptides, including insulin, derived from other neurosecretory cells^27–29^. The CC also produces a nutrient-responsive secreted peptide, Limostatin (Lst), which acts as a decretin by suppressing insulin production and secretion^30^. Given that AKH and Lst regulate metabolic responses to starvation through distinct mechanisms, these observations raise the possibility that the CC functions as a multifunctional neuroendocrine hub that coordinates organismal catabolism over the course of starvation.

In this study, we identified CG14075/Mmt as a hormone-like peptide that is produced in and secreted from the CC. We demonstrate that CG14075/Mmt is secreted into the haemolymph under both fed and starved conditions, with circulating levels increasing progressively during starvation, and that it is required for survival by supporting metabolic adaptation, including amino acid catabolism. Collectively, our findings establish CG14075/Mmt as a CC-derived hormone-like peptide that contributes to catabolic reprogramming during starvation. These findings provide a framework for understanding how amino acid metabolism is hormonally integrated into systemic energy homeostasis during nutrient deprivation in *Drosophila*.

## Results

### Identification of a CC-derived secreted peptide, CG14075/Mmt

**Fig. 1:**
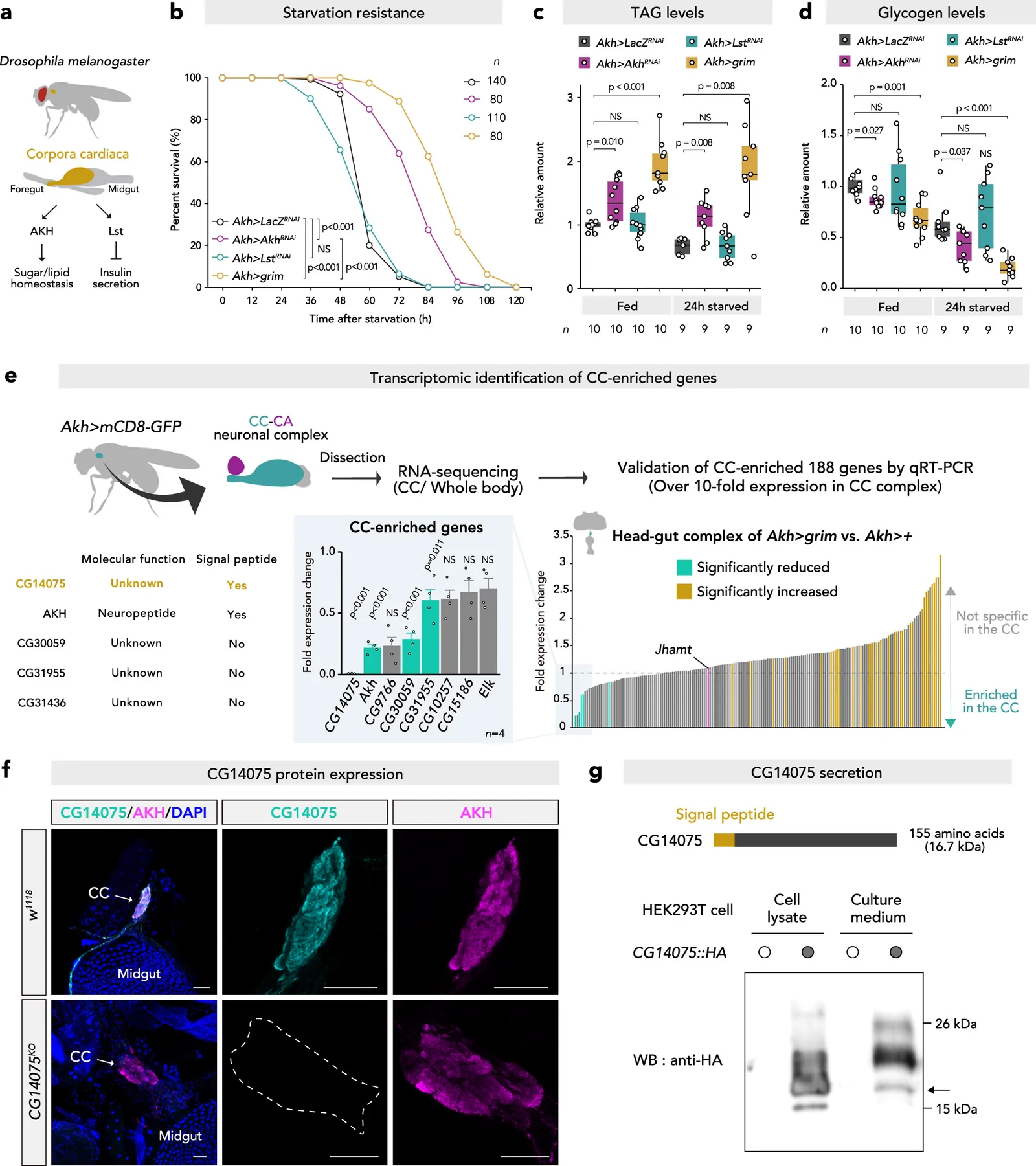
Identification of a peptide produced in the CC. **a** Schematic illustration of the corpora cardiaca (CC). The CC secretes AKH and Lst, which regulate carbohydrate/lipid metabolism and insulin secretion, respectively. **b** Starvation survival curves of virgin female flies. **c** Relative TAG levels in fed or 24 h-starved flies. **d** Relative glycogen levels in fed or 24 h-starved flies. **e** Identification of CC-derived factors by RNA-seq followed by qRT-PCR validation. CC–CA neuronal complexes and whole-body samples were isolated from *Akh* > *mCD8-GFP* flies. qPCR validation was performed using head–anterior gut complexes. Genes showing statistically significant decreases or increases are indicated in cyan and yellow, respectively. Genes downregulated in *Akh>grim* flies were defined as CC-enriched genes (right arrow). **f** Immunostaining of the CC labelled with anti-CG14075 antibody (cyan), anti-AKH antibody (magenta), and DAPI (blue). Arrows indicate the CC. Scale bars, 20 µm. Representative images from two independent experiments with similar results are shown. **g** Domain structure of CG14075 (top) and western blot analysis of CG14075::HA secretion (bottom). CG14075 contains an N-terminal signal peptide. HA-tagged CG14075 is secreted into the medium from HEK293T cells and appears as multiple bands. The arrow indicates the predicted molecular weight of CG14075::HA; the additional bands are likely due to post-translational modifications. A representative western blot from two independent experiments with similar results is shown. Statistics: Pairwise log-rank test (**b**), one-way ANOVA followed by Dunnett’s multiple comparisons test (**c**, **d**), and unpaired two-sided Welch’s t-test (**e**). NS, not significant (*P* > 0.05). Bar plots, mean ± SE. Box plots, centre line (median), box limits (first and third quartiles), and whiskers (most extreme data points within 1.5× the interquartile range). Sample sizes (*n*) are indicated in the respective panels.

In *Drosophila*, the CC is an essential neuroendocrine tissue that regulates glucose and lipid metabolism through the production of AKH and Lst (Fig. 1a). To investigate the contribution of AKH and Lst to the starvation response, we examined starvation resistance in virgin female flies with CC-specific knockdown of either *Akh* or *Lst*. As previously reported^14,24^, *Akh* knockdown increased starvation resistance and reduced consumption of triacylglycerol (TAG) during starvation (Fig. 1b, c). In contrast, *Lst* knockdown did not significantly alter starvation resistance or TAG levels compared to controls. Interestingly, genetic ablation of the CC by overexpression of the proapoptotic gene *grim* under the control of the *Akh-Gal4* driver (hereafter referred to as CC-ablated) resulted in greater starvation resistance than *Akh* knockdown alone (Fig. 1b). In addition, CC-ablated flies exhibited a diminished starvation-induced reduction in TAG levels (Fig. 1c). Paradoxically, CC-ablated flies showed significantly reduced glycogen levels compared to *Akh-* or *Lst-*knockdown flies (Fig. 1d). *Akh* knockdown was highly effective and nearly as complete as CC-ablation (Supplementary Fig. 1a). These findings suggest that the CC contributes to starvation resistance through mechanisms beyond AKH and Lst secretion. Based on these observations, we hypothesized that additional CC-derived factors contribute to starvation resilience.

To explore this hypothesis, we performed transcriptomic analysis of the CC. We dissected the tissue containing the CC and the adjacent corpus allatum (CA), which produces juvenile hormone (JH), and performed RNA-seq (Fig. 1e). Transcript levels of *Akh* and *Jhamt*, encoding a JH-biosynthesizing enzyme^31^, were significantly enriched compared to whole-body samples, confirming successful isolation of the CC-CA complex (Fig. 1e). To identify genes specifically expressed in the CC, we compared transcript levels between control and CC-ablated flies by quantitative reverse transcription (qRT)-PCR. Among 188 genes analysed, five genes (*CG14075, Akh, CG30059, CG31955*, and *CG31436*) were significantly downregulated in CC-ablated flies. Among these candidates, CG14075 showed the most pronounced reduction, with expression decreased to 0.7% of control levels, indicating strong CC-enriched expression.

We validated the CC-specific expression of *CG14075* using CC-targeted RNAi. Knockdown of *CG14075* significantly reduced its mRNA and protein levels without affecting *Akh* levels (Supplementary Fig. 1a, b). Furthermore, CRISPR/Cas9-generated *CG14075* null mutant flies lacked detectable CG14075 protein in the CC (Fig. 1f, Supplementary Fig. 1c). Notably, the mutant showed no detectable effect on developmental viability to adulthood, and the eclosed adults exhibited no obvious morphological abnormalities (Supplementary Fig. 1d). Given its strong CC-enriched expression, we focused our subsequent analyses on CG14075. *CG14075* encodes a protein with an N-terminal signal peptide, suggesting that it functions as a secreted peptide (Fig. 1g). Consistent with this prediction, expression of CG14075 in mammalian cells led to its secretion into the culture medium (Fig. 1g).

### CG14075/Mmt regulates lipid and carbohydrate metabolism during starvation

**Fig. 2:**
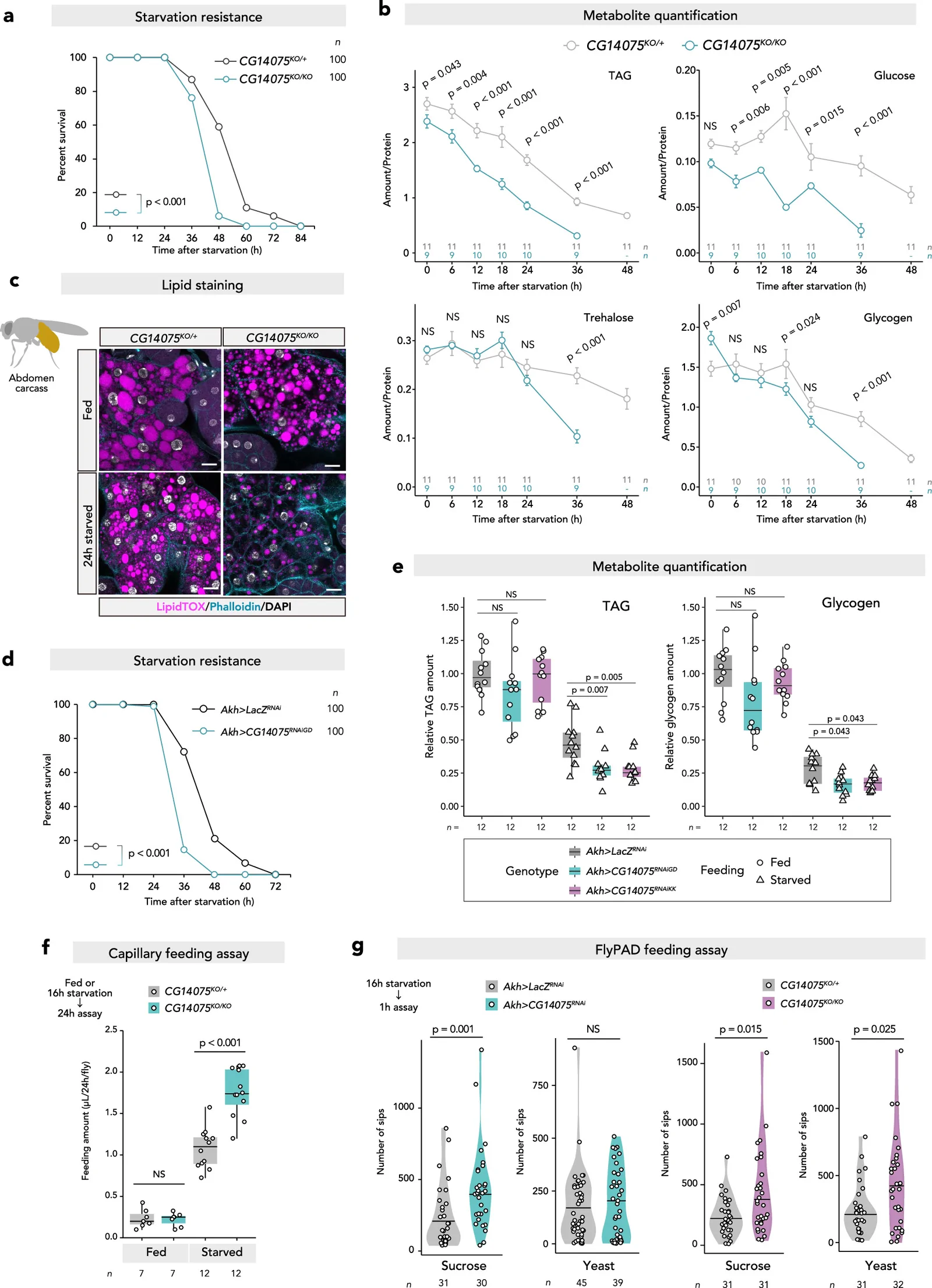
CG14075/Mmt regulates lipid and carbohydrate metabolism during starvation. **a** Starvation survival curves of control and *CG14075*^*KO*^ female flies. **b** Relative whole-body TAG, glycogen, trehalose, and glucose levels in control and *CG14075*^*KO*^ female flies. Starvation-dependent time-course reduction of each metabolite is shown. Two-way ANOVA was used to assess the effects of genotype, starvation time and their interaction. The genotype × starvation time interaction was significant for glycogen, trehalose and glucose (*P* < 0.001 for each), but not for TAG (*P* = 0.104). *n* represents biologically independent samples, each consisting of homogenates from three adult female flies. **c** LipidTOX (magenta), phalloidin (cyan), and DAPI (grey) staining of dissected fat body tissue. Scale bars, 10 µm. Representative images from two independent experiments with similar results are shown. **d** Starvation survival curves of control and CC-specific *CG14075* knockdown female flies. **e** Relative whole-body TAG and glycogen levels in flies under fed and starved conditions. **f** Feeding quantity measurement with CAFÉ assay. Flies were fed or starved for 16 h prior to experiments. **g** Feeding quantity measurement with FlyPAD assay. Flies were starved for 16 h prior to experiments. Number of sips within 60 min was plotted. Statistics: Log-rank test (**a**, **d**), Two-way ANOVA followed by Tukey’s multiple comparisons test (**b**), one-way ANOVA followed by Tukey’s multiple comparisons test (**e**; TAG), and pairwise two-sided Wilcoxon rank-sum test (**e**; glycogen), unpaired two-tailed Student’s t-test (**f**), two-sided Wilcoxon rank-sum test (**g**). NS, not significant (*P* > 0.05). Line graphs, mean ± SE. Box plots, centre line (median), box limits (first and third quartiles), and whiskers (most extreme data points within 1.5× the interquartile range). Violin plots, centre line (median). Sample sizes (*n*) are indicated in each panel.

CG14075 was recently named Marmite (Mmt) based on its role in regulating yeast feeding behaviour^32^. While CG14075/Mmt expression in specific neuronal populations in the brain contributes to this feeding behaviour, its physiological function in the CC remains unknown. Given the involvement of the CC in carbohydrate and lipid metabolism, we investigated whether regulates metabolic responses to starvation using virgin females. We first assessed starvation resistance, which correlates with stored energy reserves. *CG14075/Mmt* null mutant flies were more sensitive to starvation than controls (Fig. 2a). Metabolic analyses further indicated that *CG14075/Mmt* mutants showed accelerated depletion of energy stores during starvation. Notably, glycogen levels declined more rapidly in mutants than in controls, with a significant starvation time × genotype interaction (Fig. 2b, Supplementary Fig. 2a). Trehalose and glucose levels were also significantly reduced in mutants after 36 h of starvation, consistent with enhanced carbohydrate depletion. TAG levels differed significantly between genotypes, although the starvation time × genotype interaction did not reach statistical significance (*P* = 0.10), indicating only a trend toward genotype-dependent differences in TAG depletion (Fig. 2b). In line with the reduced lipid storage, LipidTOX staining of fat body lipid droplets revealed reduced lipid droplet area in mutants (Fig. 2c, Supplementary Fig. 2b). These results suggest that CG14075/Mmt contributes to maintaining lipid and carbohydrate homeostasis during starvation.

Similarly, CC-specific knockdown of *CG14075/Mmt* led to hypersensitivity to starvation and reduced TAG and glycogen levels following starvation (Fig. 2d, e). In contrast, fat body-specific knockdown of *CG14075/Mmt* did not affect starvation resistance or TAG levels (Supplementary Fig. 2c, d), indicating that CG14075/Mmt functions primarily through the CC rather than the fat body. To determine whether CG14075/Mmt acts in the CC to regulate metabolic responses to starvation, we restored *CG14075/Mmt* expression specifically in the CC of *CG14075/Mmt* null mutant flies. CC-specific re-expression of *CG14075/Mmt* slightly but significantly rescued the starvation-sensitive phenotype as well as the reduction in TAG and glycogen levels observed in mutant flies (Supplementary Fig. 2e, f). This partial phenotypic rescue was accompanied by a modest restoration of CG14075/Mmt protein and mRNA levels in the CC (Supplementary Fig. 2g, h). Collectively, these results support a functional role for CC-derived CG14075/Mmt in systemic metabolic regulation.

To exclude potential developmental effects, we performed adult-specific knockdown of *CG14075/Mmt* using temperature-sensitive Gal80 (Supplementary Fig. 3a). Adult-specific *CG14075/Mmt* knockdown after eclosion resulted in reduced TAG and glycogen levels, similar to those observed in constitutive knockdown flies (Supplementary Fig. 3b). These results indicate that the metabolic phenotypes associated with CG14075/Mmt deficiency are acquired during adulthood rather than arising from developmental defects.

Metabolic responses and their regulatory mechanisms vary by sex and reproductive status^33,34^. Therefore, we examined starvation phenotypes in males and mated females. In both cases, *CG14075/Mmt* null mutant flies showed increased starvation sensitivity and accelerated consumption of TAG and glycogen, similar to that observed in mutant virgin females (Supplementary Fig. 3c, d). Notably, *CG14075/Mmt* mutants tended to have lower TAG and glycogen levels under feeding conditions, suggesting that CG14075/Mmt regulates energy store levels even under *ad libitum* feeding conditions. These results suggest that CG14075/Mmt regulates energy metabolism irrespective of sex or mating status.

Given the reciprocal relationship between energy reserves and feeding behaviour^35^, we assessed food intake using the capillary feeding (CAFÉ) assay^36^. *CG14075/Mmt* mutant flies consumed more food containing sucrose and yeast lysate than controls following starvation (Fig. 2f), and adult-specific *CG14075/Mmt* knockdown flies showed a similar increase (Supplementary Fig. 3e). When sucrose and yeast were provided separately, *CG14075/Mmt* mutant flies showed a significant increase in sucrose intake, whereas yeast consumption exhibited only a trend toward increased intake (Supplementary Fig. 3f). Using a FlyPAD system, which allows automated short-term quantification of feeding behaviour^37^, we likewise detected increased sucrose intake in both *CG14075/Mmt* knockdown and mutant flies, whereas increased yeast intake was observed only in the mutants (Fig. 2g). Since *CG14075/Mmt* expressed in brain neurons has been reported to regulate yeast appetite^32^, this difference may reflect distinct nutrient requirements and regulatory mechanisms between CC-specific knockdown and knockout flies.

To distinguish the functional roles of brain- and CC-derived CG14075/Mmt, we knocked down its expression in neurons using *VT013947-Gal4*, which is expressed in CG14075/Mmt-positive brain neurons^32^, but not in the CC (Supplementary Fig. 4a). *CG14075/Mmt* knockdown in brain neurons neither affected starvation resistance nor reduced TAG and glycogen levels (Supplementary Fig. 4b, c). These data suggest that brain-derived CG14075/Mmt regulates appetite for yeast, whereas CC-derived CG14075/Mmt plays a functional role in systemic metabolic adaptation to starvation.

### CG14075/Mmt is secreted into the haemolymph in response to starvation

**Fig. 3:**
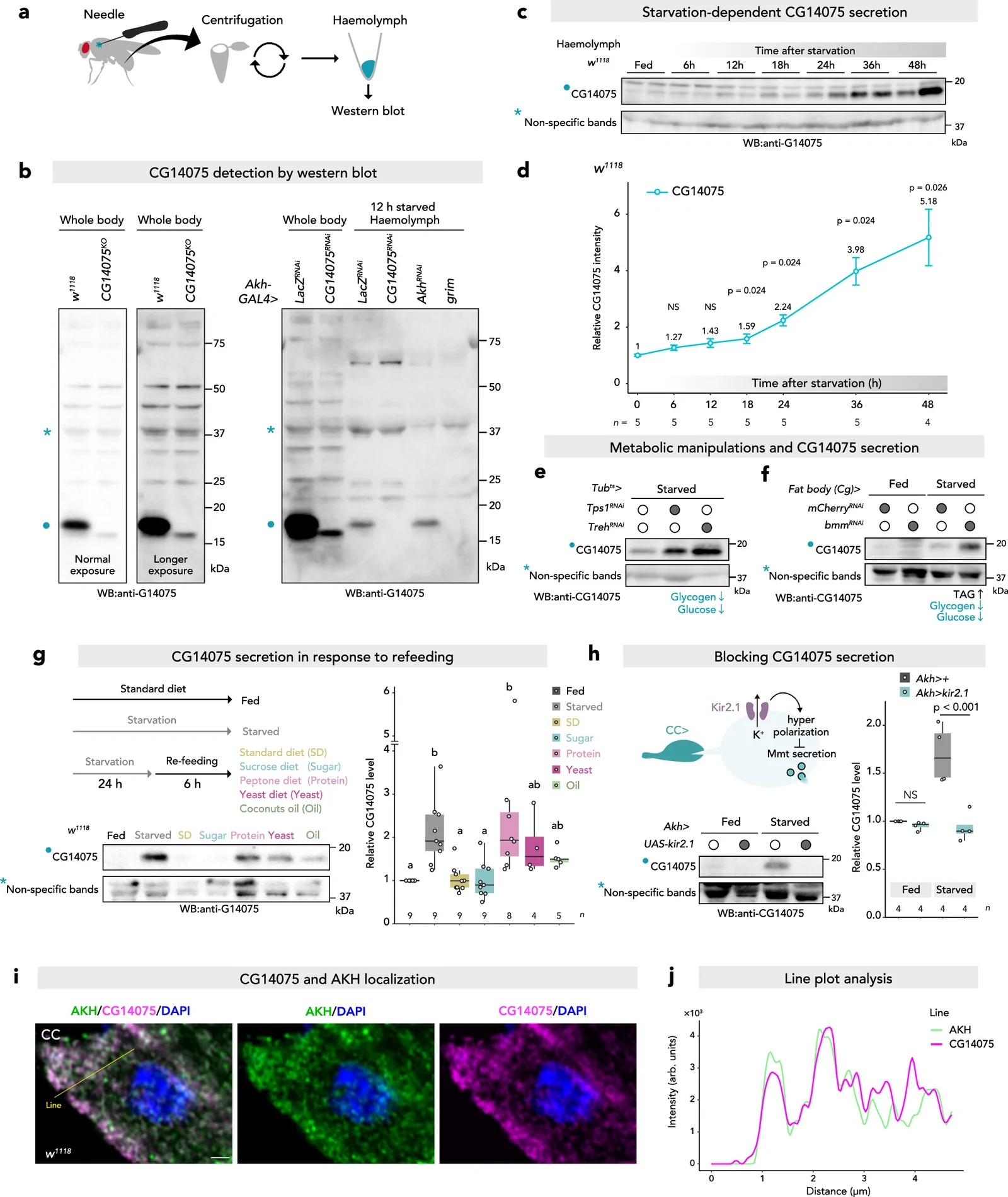
CG14075/Mmt is secreted into the haemolymph upon starvation. **a** An illustration of haemolymph collection. **b** Detection of CG14075 protein by western blot. **c** CG14075 secretion along the starvation timeline. *w*^*1118*^ flies were used. **d** Relative intensity of the CG14075 band. **e** CG14075 secretion in control and trehalose metabolic gene knockdown flies. **f** CG14075 secretion in control and *bmm* knockdown flies. **g** CG14075 secretion in response to refeeding. Flies were refed with each diet for 6 h following 24 h of starvation. *w*^*1118*^ flies were used. Different letters indicate statistically significant differences between groups. Groups sharing the same letter are not significantly different. Exact *P* values are provided in the Source Data file. **h** CG14075 secretion in control and *kir2.1-*overexpressing flies. **i** High-resolution immunostaining images of CC from *w*^*1118*^ labelled by anti-CG14075 antibody (magenta), anti-AKH antibody (green), and DAPI (blue). Scale bar, 1 μm. Representative images from two independent experiments with similar results are shown. **j** Intensity distribution of CG14075 and AKH along the line shown in (i) Blue dots and asterisks indicate CG14075-specific bands and non-specific bands, respectively, in (**b**, **c**, **e**–**h**). Representative western blots from two independent experiments with similar results are shown. Statistics: Pairwise two-sided Wilcoxon rank-sum test (**d**), and one-way ANOVA followed by Tukey’s multiple comparisons test (**g**, **h**). NS, not significant (*P* > 0.05). Line graphs, mean ± SE. Box plots, centre line (median), box limits (first and third quartiles), and whiskers (most extreme data points within 1.5× the interquartile range). Sample sizes (n) are indicated in each panel.

AKH is known to be secreted from the CC into the haemolymph during starvation^28^. To determine whether CG14075/Mmt is similarly regulated, we performed Western blotting on whole-body and haemolymph samples collected from starved flies to detect circulating CG14075/Mmt (Fig. 3a). The anti-CG14075/Mmt antibody detected a specific band of ~15–20 kDa in whole-body adult samples from control animals, but not from *CG14075/Mmt* mutants (Fig. 3b). This band was also undetectable in CC-specific *CG14075/Mmt* knockdown adults, suggesting that CG14075/Mmt is predominantly produced in the CC. In the haemolymph, the CG14075/Mmt band was present in control flies but absent in *CG14075/Mmt* knockdown and CC-ablated flies (Fig. 3b). In contrast, knockdown of *CG14075/Mmt* in brain neurons did not alter its haemolymph levels or whole-body mRNA levels (Supplementary Fig. 4d, e).

Interestingly, circulating CG14075/Mmt increased progressively during starvation. It was detectable under fed conditions, increased approximately two-fold within 18–24 h, and peaked at 36–48 h (Fig. 3c, d). This timing coincided with the decline in TAG and glycogen levels (Fig. 2b), suggesting that CG14075/Mmt secretion is linked to systemic energy reduction. To examine this possibility, we disrupted trehalose metabolism by RNAi-mediated knockdown of the trehalose-synthesizing enzyme *Trehalose-6-phosphate synthase 1* (*Tps1)* or the hydrolysing enzyme *Trehalase (Treh)*. These manipulations enhance glucose and glycogen consumption during starvation^38,39^. Circulating CG14075/Mmt levels were significantly increased in both *Tps1* and *Treh* knockdown flies (Fig. 3e), suggesting that carbohydrate depletion promotes CG14075/Mmt release. We next examined whether lipid metabolism also affects CG14075/Mmt secretion. Knockdown of *brummer (bmm)*, which encodes an adipocyte TAG lipase^40^, increased CG14075/Mmt in the haemolymph after starvation (Fig. 3f). Notably, *bmm* knockdown flies displayed impaired lipolysis and instead relied more heavily on glycogen consumption during starvation (Supplementary Fig. 5a), further suggesting that CG14075/Mmt secretion is sensitive to carbohydrate availability rather than directly to TAG levels. Consistent with this idea, refeeding with sucrose robustly suppressed starvation-induced CG14075/Mmt secretion (Fig. 3g). By contrast, peptone refeeding had little effect, whereas yeast or coconut oil refeeding led to only a partial reduction in circulating CG14075/Mmt levels (Fig. 3g). Together, these results indicate that carbohydrate availability is a primary determinant of CG14075/Mmt secretion, while protein- or lipid-derived nutrients are insufficient to fully suppress its release following starvation.

To determine whether the increase in circulating CG14075/Mmt during starvation and after peptone refeeding originates from the CC, we knocked down *CG14075/Mmt* specifically in the CC. CC-specific knockdown abolished the increase in circulating CG14075/Mmt levels observed under both conditions (Supplementary Fig. 5b), indicating that the CC is the primary source of CG14075/Mmt released into the haemolymph. In contrast, inhibition of the TOR pathway in the fat body by expression of a dominant-negative form of mTOR (mTOR^DN^) failed to induce CG14075/Mmt secretion under fed conditions (Supplementary Fig. 5c), suggesting that reduced fat body TOR signalling is not sufficient to trigger its release. Finally, suppressing CC cell excitability by expression of the inward-rectifying potassium channel Kir2.1 markedly reduced starvation-induced CG14075/Mmt secretion (Fig. 3h), indicating that CG14075/Mmt release requires CC cell excitability.

The amount of AKH stored in CC cell bodies is known to decrease during starvation as a result of secretion^19,21,24^. We therefore compared the temporal dynamics of AKH and CG14075/Mmt protein levels in the CC. AKH protein levels declined from 12 h after starvation onset, while CG14075/Mmt levels started to decrease later, around 18 h (Supplementary Fig. 5d, e). mRNA levels of both genes remained largely constant during starvation, suggesting that the observed decrease in protein levels reflects secretion rather than reduced transcription.

Immunostaining and higher-resolution imaging revealed spatially heterogeneous, punctate staining of CG14075/Mmt and AKH within CC cells (Fig. 3i). Line plot analysis showed that their intensity profiles did not fully overlap, suggesting possible differences in their subcellular distribution (Fig. 3j). To further examine the localization of CG14075/Mmt relative to the plasma membrane, we expressed the plasma membrane marker mCD8-GFP in CC cells. CG14075/Mmt signals were observed close to, but not fully overlapping with, mCD8-GFP-labelled plasma membrane signals (Supplementary Fig. 5f). These observations suggest a spatial relationship between CG14075/Mmt-positive intracellular structures and membrane-proximal regions. Importantly, these punctate CG14075/Mmt signals were abolished upon CC-specific knockdown, confirming the specificity of the staining (Supplementary Fig. 5g).

In CC axon-like projections to the anterior midgut, AKH signals increased in a starvation-dependent manner (Supplementary Fig. 5h). In contrast, CG14075/Mmt did not show a comparable starvation-induced increase. Together, these observations suggest that CG14075/Mmt and AKH may be differentially organised within CC cells, despite being produced in the same endocrine tissue.

### CG14075/Mmt regulates amino acid catabolism during starvation

**Fig. 4:**
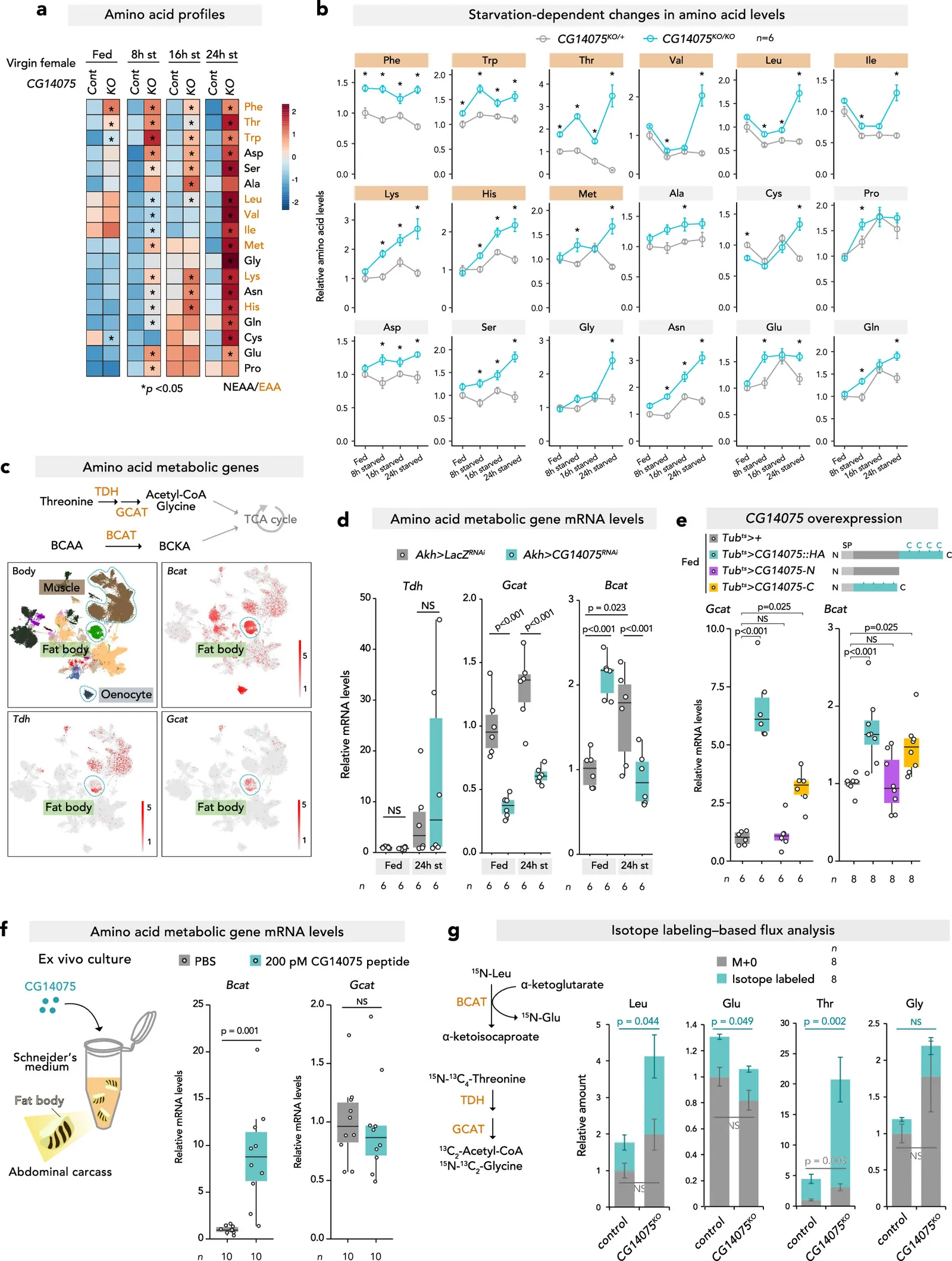
CG14075/Mmt regulates amino acid catabolism. **a** Heatmap of amino acid profiles of control and *CG14075*^*KO*^ virgin female flies. EAAs are highlighted in orange. Asterisks indicate statistical significance between control and *CG14075*^*KO*^ under fed and each starved condition. **b** Changes in amino acid levels under fed conditions and at 8 h, 16 h, and 24 h of starvation. Asterisks indicate statistical significance between control and *CG14075*^*KO*^ under each condition. **c** Single-cell RNA expression profiles of the threonine catabolic genes *Tdh* and *Gcat*, and the BCAA catabolic gene *Bcat*, visualized using the Fly Cell Atlas. **d** *Tdh, Gcat*, and *Bcat* mRNA expression profiles in the abdominal carcass of control and *CG14075* knockdown flies. Flies were fed or starved for 24 h prior to sampling. **e** Relative mRNA expression of *Gcat* and *Bcat* of the abdominal carcass of control, full-length *CG14075* overexpressing (*Tub*^*ts*^ > *CG14075::HA*) flies, *CG14075 N-terminal region* overexpressing (*Tub*^*ts*^ > *CG14075-N*) flies, and *CG14075 C-terminal region* overexpressing (*Tub*^*ts*^ > *CG14075-C*) flies. **f** An illustration of the ex vivo culture system for the fly abdominal carcass and mRNA expression profiles of *Bcat* and *Gcat*. Dissected fly abdomens were incubated in Schneider’s *Drosophila* medium for 2 h with or without 200 pM CG14075 synthetic peptide. **g** Isotope tracing experiments for control (*CG14075*^*KO/+*^) and *CG14075*^*KO/KO*^. After 24 h of starvation, flies were transferred to agar supplemented with 3 mM ^15^N-Leu or ^15^N-^13^C_4_-Thr for 24 h. Cyan statistical annotations indicate comparisons between isotope-labelled metabolites, whereas grey statistical annotations indicate comparisons between the corresponding unlabelled metabolites. Statistics: two-sided Wilcoxon rank-sum test (**a**, **b**), one-way ANOVA followed by Tukey’s multiple comparisons test (**d**), one-way ANOVA followed by Dunnett’s test (**e**), Unpaired two-tailed Welch’s t-test (**f**), and unpaired two-tailed Student’s t-test (**g**). *P < 0.05; NS, not significant (*P* > 0.05). Asterisks indicate statistical significance; exact *P* values are provided in the Source Data file. Bar plots and line graphs, mean ± SE. Box plots, centre line (median), box limits (first and third quartiles), and whiskers (most extreme data points within 1.5× the interquartile range). Sample sizes (*n*) are indicated in each panel.

Our results indicate that CG14075/Mmt secretion increases gradually during starvation, becoming further elevated at mid-to-late (e.g., 18–24 h) stages when fat and glycogen stores are substantially depleted. During prolonged starvation, organisms increasingly mobilize amino acids to support gluconeogenesis and ketogenesis^41^. To understand how amino acid metabolism is affected in the absence of CG14075/Mmt, we analysed *CG14075/Mmt* knockout flies, which exhibit excessive depletion of lipid and carbohydrate stores. In control flies, several amino acids, including threonine and branched-chain amino acids (BCAAs; valine, leucine, and isoleucine), decreased during starvation (Fig. 4a, b). In contrast, *CG14075/Mmt* mutants showed a marked increase in threonine and BCAAs after 24 h of starvation. More broadly, *CG14075/Mmt* mutants exhibited starvation-dependent accumulation of both essential and non-essential amino acids (Fig. 4a, b). A similar accumulation of threonine and BCAAs was observed in mated female mutants 48 h after starvation (Supplementary Fig. 6a, b). Importantly, this starvation-induced amino acid accumulation was partially rescued by CC-specific overexpression of CG14075/Mmt, indicating that CC-derived CG14075/Mmt contributes to the regulation of amino acid metabolism, in parallel with its effects on TAG and glycogen homeostasis (Supplementary Fig. 6c, d).

We next examined the expression of key catabolic enzymes involved in the metabolism of threonine and BCAAs, which showed the most pronounced accumulation in *CG14075/Mmt* mutants during starvation. Threonine is metabolized by threonine dehydrogenase (Tdh) and glycine C-acetyltransferase (Gcat)^42^. A single-cell transcriptome atlas^43^ suggests that *Tdh* is highly expressed in the fat body and muscle, while *Gcat* is predominantly expressed in the fat body (Fig. 4c). BCAAs are metabolized by branched-chain amino acid transaminase (Bcat), which is expressed in several tissues including the fat body, oenocytes, and muscle (Fig. 4c). Upon starvation, control flies upregulated *Gcat* and *Bcat* expression in the abdominal carcass (Fig. 4d), suggesting active catabolism of threonine and BCAAs. However, this induction was attenuated in *CG14075/Mmt* knockdown flies, consistent with the observed accumulation of these amino acids. Although *Tdh* expression showed a modest increase during starvation, these changes were not statistically significant (Fig. 4d).

To test whether CG14075/Mmt is sufficient to induce amino acid catabolism, we first performed CC-specific overexpression of CG14075/Mmt. This manipulation led to a modest increase in whole-body *CG14075/Mmt* mRNA levels and induced *Gcat* expression in abdominal carcass (Supplementary Fig. 7a). However, overexpression of neuropeptides within their native neurons is not always sufficient to induce signalling, because secretion is often regulated non-cell-autonomously^14,44^.

To more robustly induce CG14075/Mmt signalling, we used a temporally controlled overexpression system (*Tub-GAL4, Tub-GAL80*^*ts*^). Transient induction of CG14075/Mmt for 24 h under fed conditions upregulated *Gcat* and *Bcat* expression (Supplementary Fig. 7b). Consistently, this overexpression reduced whole-body BCAA and threonine levels (Supplementary Fig. 7c). The reduction in threonine was abolished by knockdown of *Tdh* (Supplementary Fig. 7d), indicating that the decrease is mediated by enhanced threonine catabolism.

Notably, *CG14075/Mmt* mutants exhibited altered amino acid levels not only at 24 h starvation, but also under fed conditions and at early stages of starvation (e.g., 8 h) (Fig. 4a, b). Consistent with this observation, mRNA expression of *Bcat* and the lipase *bmm* was already elevated in *CG14075/Mmt* mutants under fed conditions (Supplementary Fig. 7e). Taken together with the early reduction in lipid stores and the observation that CG14075/Mmt secretion is detectable at early stages of starvation, these findings suggest that CG14075/Mmt contributes to the regulation of amino acid and lipid metabolism across nutritional states, including the fed condition, early starvation, and mid-to-late stages of starvation.

CG14075/Mmt contains a C-terminal domain with evolutionarily conserved cysteine residues (Supplementary Fig. 8a)^32^. Notably, the regions surrounding these four conserved cysteine residues exhibit higher amino acid conservation compared with the corresponding N-terminal region. In Deuterostomia, this region shows similarity to the C-terminal region of neuropeptide B (NPB) (Supplementary Fig. 8a)^32^. Structural modelling using AlphaFold2^45,46^ predicted a hairpin-like conformation in the C-terminal region, with cysteine residues Cys^101^–Cys^110^ and Cys^89^–Cys^124^ forming disulfide bonds, while the N-terminal region was predicted to be flexible (Supplementary Fig. 8b-f). To test the functional role of each region, we generated transgenic flies expressing either the C-terminal or N-terminal region of CG14075/Mmt. Overexpression of the C-terminal fragment induced *Gcat* and *Bcat* expression, whereas the N-terminal fragment had no effect (Fig. 4e), indicating that the C-terminal cysteine-rich region is functionally essential. Given that the effect of the C-terminal fragment was weaker than that of the full-length transgene, the N-terminal region may play a supportive role in CG14075/Mmt function.

To test whether CG14075/Mmt can act on peripheral tissues, we synthesized a recombinant peptide corresponding to the C-terminal region with two predicted disulfide bonds. Ex vivo culture of fly abdominal carcass with the CG14075/Mmt peptide for 2 h induced *Bcat* expression, with peak induction between 10 and 100 pM (Fig. 4f, Supplementary Fig. 8g). This concentration range is physiologically relevant and similar to that of Dilps (100–350 pM) and AKH (approximately 300 pM)^47,48^. Although *Gcat* expression was not induced under these conditions, these results suggest that abdominal peripheral tissues are capable of responding to CG14075/Mmt by altering amino acid catabolic gene expression.

We further conducted stable isotope tracing to assess whether CG14075/Mmt influences amino acid turnover. Feeding flies with ^15^N-leucine or ^15^N-^13^C_4_-threonine during starvation revealed that labelled amino acids accumulated to higher levels in *CG14075/Mmt* mutants than in controls (Fig. 4g). Moreover, ^15^N-glutamate, a product of BCAA catabolism by Bcat, was reduced in mutants. Although ^15^N-^13^C_2_-glycine, a product of threonine catabolism, did not differ between genotypes, ^15^N-^13^C_4_-threonine itself accumulated in mutants. These results suggest that leucine and threonine are metabolized more slowly in *CG14075/Mmt* mutants. Together, these findings support a role for CG14075/Mmt in promoting amino acid catabolism during starvation, likely through regulation of catabolic enzyme expression and, potentially, enzymatic activity.

### Threonine catabolism in the fat body is required for starvation resistance

**Fig. 5:**
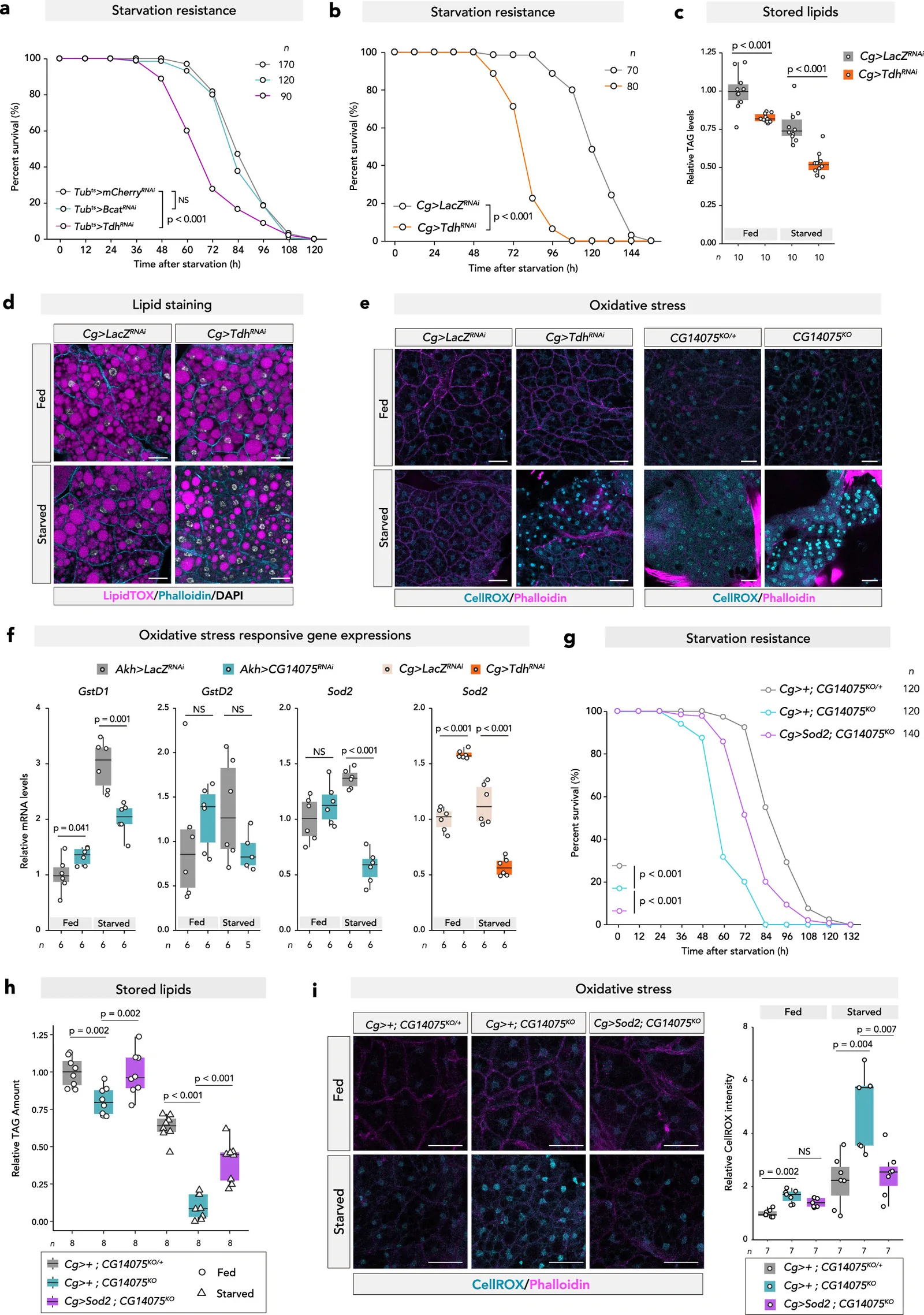
Threonine catabolism is required for the starvation response. **a** Starvation survival curves of control and whole-body amino acid catabolic gene knockdown female flies. **b** Starvation survival curves of control and adipose tissue-specific *Tdh* knockdown female flies. **c** Relative TAG levels in control and adipose tissue-specific *Tdh* knockdown flies. **d** LipidTOX (magenta), phalloidin (cyan), and DAPI (grey) staining of dissected fat body tissue from control and *Tdh* knockdown flies. Scale bars, 10 µm. **e** Left, CellROX staining (cyan) of the fat body from control and *Tdh* knockdown flies. Right, CellROX staining of the fat body from control and *CG14075*^*KO*^ flies. Phalloidin (magenta) labels muscle and fat body cell membranes. Scale bars, 20 µm. **f** Relative mRNA expression levels of oxidative stress–responsive genes in abdominal carcasses of fed or 24 h-starved control and *CG14075* knockdown flies (left), and control and fat body-specific *Tdh* RNAi flies (right). **g** Starvation survival curves of control, *CG14075*^*KO*^, and adipose tissue-specific *Sod2* overexpression flies. **h** Relative TAG levels in flies as in (**g**). **i** CellROX staining (cyan) of the fat body from flies as in (**g**). Phalloidin (magenta) labels muscle and fat body cell membranes. Scale bars, 20 µm. Statistics: Log-rank test (**a**, **b**), pairwise log-rank test (**g**), unpaired two-tailed Student’s t-test (**c**, **f**), one-way ANOVA followed by Tukey’s multiple comparisons test (**h**), and pairwise two-sided Wilcoxon rank-sum test (**i**). NS, not significant (*P* > 0.05). Representative images from two independent experiments with similar results are shown in (d, e, g). Box plots, centre line (median), box limits (first and third quartiles), and whiskers (most extreme data points within 1.5× the interquartile range). Sample sizes (*n*) are indicated in each panel.

Given the impaired amino acid catabolism in *CG14075/Mmt* mutants, we next tested whether disruption of threonine and BCAA catabolism affects starvation tolerance. Temporal knockdown of *Tdh* in adults using *Tub-Gal4*^*ts*^ led to heightened starvation sensitivity, whereas knockdown of *Bcat* had no significant effect (Fig. 5a). Amino acid profiling revealed that *Bcat* knockdown led to BCAA accumulation, whereas *Tdh* knockdown caused broader changes in amino acid levels, including isoleucine, leucine, glycine, serine, and threonine (Supplementary Fig. 9a, b).

The fat body, where *Tdh* and *Gcat* are highly expressed, serves as the major nutrient storage organ and a central hub for systemic energy metabolism in insects^49^. We found that tissue-specific knockdown of *Tdh* in the adult fat body recapitulated the starvation-sensitive phenotype (Fig. 5b). Fat body-specific *Tdh* knockdown also reduced TAG levels under both fed and starved conditions (Fig. 5c). Consistent with this, LipidTOX staining revealed a decreased lipid droplet area in the knockdown flies (Fig. 5d, Supplementary Fig. 9c). To further examine how fat body threonine catabolism contributes to starvation adaptation, we performed stable isotope tracing using ^15^N,^13^C_4_-threonine in fat body-specific *Tdh* knockdown flies. *Tdh* knockdown reduced label incorporation from threonine into glycine and serine, consistent with impaired Tdh-dependent threonine catabolism (Supplementary Fig. 9d). In addition, labelled fumarate was reduced, suggesting that threonine-derived carbon contributes, at least in part, to TCA-cycle-related metabolism during starvation. These results support the idea that fat body Tdh contributes to starvation adaptation by linking threonine catabolism to glycine/serine metabolism and mitochondrial metabolic intermediates.

Starvation induces oxidative stress, and both *Akh* mutants and flies undergoing excessive lipid catabolism exhibit heightened sensitivity to oxidative damage^15,50^. Given the lipid-consuming phenotype, we hypothesized that CG14075/Mmt-dependent threonine catabolism contributes to maintaining redox homeostasis during starvation. To test this, we assessed oxidative stress levels in the fat body using CellROX, a ROS-sensitive fluorescent probe. Under starvation, both *CG14075/Mmt* mutants and fat body-specific *Tdh* knockdown flies showed significantly increased fluorescence compared to controls (Fig. 5e, Supplementary Fig. 9e), suggesting elevated oxidative stress in the fat body. We next examined sensitivity to the oxidative stress inducer paraquat. *CG14075/Mmt* mutants showed increased susceptibility to paraquat when it was added to food (Supplementary Fig. 10a, b). When paraquat was administered during starvation, it further increased susceptibility in both the mutants and *Tdh* knockdown flies (Supplementary Fig. 10c-e), suggesting that CG14075/Mmt and Tdh-dependent threonine catabolism protect against oxidative stress under nutrient-deprived conditions. Conversely, antioxidant vitamin C modestly improved starvation survival (Supplementary Fig. 10f, g).

We next examined glutathione, a major redox-active metabolite. Under starvation conditions, both *CG14075/Mmt* mutants and fat body–specific *Tdh* knockdown flies exhibited increased levels of oxidized glutathione (GSSG) (Supplementary Fig. 10h, i). However, reduced glutathione (GSH) was also elevated, and the GSSG/GSH ratio showed only a modest increasing trend during starvation. To investigate the basis of the increased GSH levels, we quantified the mRNA levels of glutathione biosynthetic enzymes (*gclc, gclm*, and *gss*) in abdominal carcass. Although *gclc* and *gss* expression were elevated under fed conditions in both genotypes (Supplementary Fig. 10j, k), these changes did not clearly explain the increased GSH levels observed during starvation.

To further explore the link between oxidative stress and starvation hypersensitivity, we examined the expression of oxidative stress-responsive genes. In control flies, starvation induced expression of *glutathione S-transferase D1* (*GstD1*), a canonical oxidative stress marker^51^; however, this induction was lost in *CG14075/Mmt* knockdown flies (Fig. 5f). In contrast, *GstD2*, another glutathione-related gene, showed no significant changes between control and knockdown flies. Expression of *superoxide dismutase 2* (*Sod2*), which neutralizes mitochondrial reactive oxygen species (ROS)^52^, was also reduced in *CG14075/Mmt* knockdown flies and *Tdh* knockdown flies (Fig. 5f). Given that threonine and BCAA catabolism primarily occurs in mitochondria, we focused on Sod2 as a key regulator of mitochondrial oxidative stress. To test whether mitochondrial ROS contributes to the observed phenotypes, we overexpressed *Sod2* specifically in the fat body in the *CG14075/Mmt* mutant background. This manipulation partially rescued starvation sensitivity and attenuated the accelerated TAG depletion observed in *CG14075/Mmt* mutants (Fig. 5g, h). Furthermore, *Sod2* overexpression suppressed the elevation of ROS levels in the fat body during starvation (Fig. 5i). However, fat body-specific *Sod2* overexpression did not suppress the starvation-induced accumulation of threonine, BCAAs, or other amino acids in *CG14075/Mmt* mutants (Supplementary Fig. 11a, b). These results suggest that mitochondrial oxidative stress contributes to starvation hypersensitivity and excessive lipid consumption without correcting the underlying defects in amino acid metabolism. Collectively, our findings support a model in which impaired amino acid catabolism in *CG14075/Mmt* mutants promotes oxidative stress, which in turn contributes, at least in part, to starvation hypersensitivity and accelerated lipid consumption.

We next examined nutrient- and amino acid-responsive signalling pathways, including mTOR and AMPKα. The level of phosphorylated S6K, a downstream target of mTOR signalling, was not markedly altered in either *CG14075/Mmt* mutants or *Tdh* knockdown flies (Supplementary Fig. 11c, d). Similarly, AMPKα phosphorylation did not show a common change between these two conditions. Notably, the total level of AMPKα was reduced in *Tdh* knockdown flies, resulting in a higher p-AMPKα/AMPKα ratio.

Together, these results suggest that CG14075/Mmt-dependent regulation of amino acid metabolism, particularly threonine catabolism, contributes to starvation adaptation by modulating oxidative stress and preventing excessive lipid depletion.

### CG14075/Mmt and AKH coordinately regulate starvation responses

**Fig. 6:**
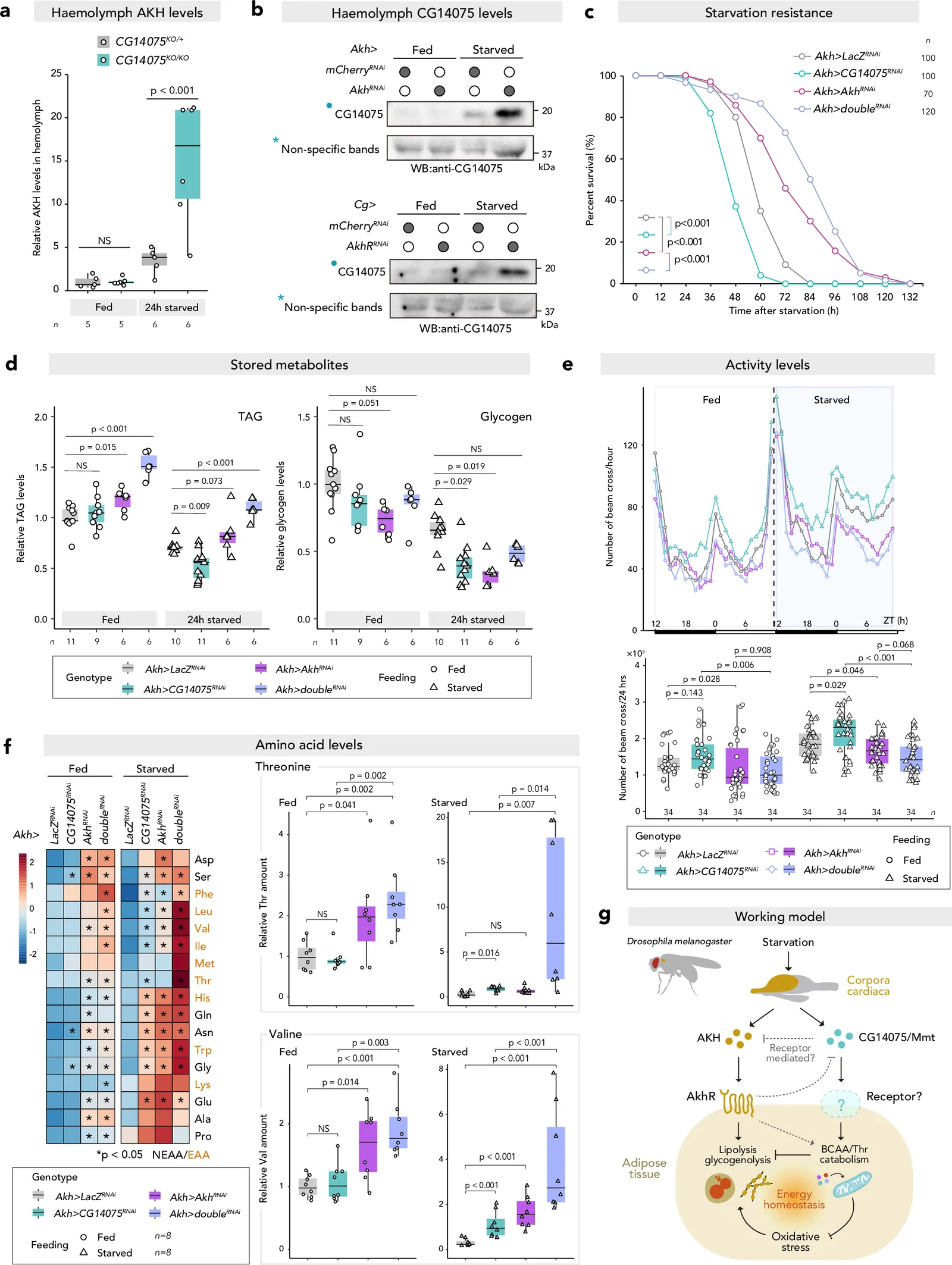
CG14075/Mmt and AKH cooperatively regulate the starvation response. **a** Relative circulating AKH levels in the haemolymph. **b** Detection of circulating CG14075 in *Akh* and *AkhR* knockdown flies. Blue dots and asterisks indicate CG14075-specific bands and non-specific bands (used as loading controls), respectively. Representative western blots from two independent experiments with similar results are shown. **c** Starvation survival curves of control flies and flies with CC-specific knockdown of *CG14075*, *Akh* or both genes. double RNAi denotes simultaneous *CG14075* and *Akh* knockdown. **d** Relative TAG (left) and glycogen (right) levels as in (**c**). **e** Top, activity profiles showing the number of beam crossings over time in control and knockdown flies under fed and starved conditions. Bottom, quantification of activity counts per fly under fed and starved conditions. **f** Left, heatmap of amino acid profiles in control and CC-specific *CG14075* and/or *Akh* knockdown female flies. EAAs are highlighted in orange. Asterisks indicate statistical significance between control and each RNAi genotype under fed and starved conditions. Right, relative levels of threonine and valine. **g** Proposed model of CG14075 function. CG14075 and AKH contribute to the regulation of lipid, carbohydrate, and amino acid metabolism across the starvation timeline to maintain energy homeostasis. Statistics: Unpaired two-tailed Student’s t-test (**a**), pairwise log-rank test (**c**), pairwise Wilcoxon rank-sum test (**d**; TAG, **e**, **f**), and one-way ANOVA followed by Tukey’s multiple comparisons test (**d**; glycogen). NS, not significant (*P* > 0.05). Box plots, centre line (median), box limits (first and third quartiles), and whiskers (most extreme data points within 1.5× the interquartile range). Sample sizes (*n*) are indicated in each panel.

Because loss of CG14075/Mmt causes excessive lipid depletion and starvation sensitivity during starvation, we asked whether these phenotypes are driven by dysregulated AKH secretion. In *CG14075/Mmt* knockout flies, AKH secretion was markedly elevated during starvation, as quantified by liquid chromatography-tandem mass spectrometry (LC-MS/MS)^53^ (Fig. 6a). Conversely, knockdown of *Akh* in the CC or *AkhR* in the fat body resulted in increased CG14075/Mmt secretion during starvation (Fig. 6b, Supplementary Fig. 12a). These results suggest that perturbation of one hormonal pathway is associated with altered secretion of the other, consistent with a functional interaction between CG14075/Mmt and AKH signalling during starvation.

We next tested whether the metabolic phenotypes caused by loss of CG14075/Mmt depend on AKH. Simultaneous knockdown of *CG14075/Mmt* and *Akh* increased starvation resistance and retained more TAG after starvation compared with either single knockdown (Fig. 6c, d). Notably, glycogen levels, which were reduced by single knockdown of either *CG14075/Mmt* or *Akh*, were preserved during starvation in the double-knockdown animals. Thus, the excessive depletion of lipid and glycogen stores caused by *CG14075/Mmt* knockdown is largely suppressed by reducing AKH signalling.

We further examined behavioural activity, as AKH signalling promotes food-seeking behaviour and starvation-induced hyperactivity^14,15,54^. As expected, *Akh* knockdown reduced starvation-induced hyperactivity, whereas *CG14075/Mmt* knockdown enhanced it (Fig. 6e). This increase in activity was fully suppressed by simultaneous knockdown of *Akh*. These results suggest that the excessive activity caused by *CG14075/Mmt* knockdown results from AKH hypersecretion.

Finally, we examined amino acid levels to determine whether this phenotype is also mediated by AKH. Under fed conditions, *CG14075/Mmt* knockdown caused only minor changes in amino acid levels, whereas *Akh* knockdown and the double-knockdown affected a larger number of amino acids (Fig. 6f). Under starvation conditions, *CG14075/Mmt* knockdown led to pronounced accumulation of threonine and BCAAs as observed in knockout animals (Fig. 6f, Supplementary Fig. 12b). In contrast, *Akh* knockdown significantly increased BCAA levels but did not significantly elevate threonine. Notably, simultaneous knockdown of *CG14075/Mmt* and *Akh* resulted in even greater accumulation of both BCAAs and threonine than observed in either single knockdown. A similar trend was observed in adult-specific knockdown experiments, in which the double knockdown again showed a tendency toward threonine and BCAA accumulation (Supplementary Fig. 12c). Thus, unlike the lipid and behavioural phenotypes, the amino acid accumulation phenotype caused by *CG14075/Mmt* knockdown was not rescued by *Akh* knockdown. These results suggest that CG14075/Mmt regulates amino acid metabolism at least partly independently of AKH signalling.

Taken together, our results identify CG14075/Mmt as a starvation-associated hormone-like peptide that contributes to threonine and BCAA catabolism and redox homeostasis during nutrient deprivation (Fig. 6g). In parallel with AKH, which promotes lipid mobilization during starvation, CG14075/Mmt contributes to starvation adaptation by supporting amino acid catabolism. Although our data suggest a functional interaction between CG14075/Mmt and AKH signalling, the mechanistic relationship between these pathways remains incompletely understood. These findings suggest that the CC coordinates starvation responses through multiple hormonal outputs that regulate distinct but interconnected aspects of systemic metabolism.

## Discussion

During starvation, organisms sequentially mobilize internal energy reserves, including glycogen, lipids, and eventually amino acids, to sustain survival. Although the general sequence of nutrient utilization is well conserved across species, the hormonal mechanisms that coordinate these metabolic transitions remain incompletely understood. Here, we identify CG14075/Mmt as a starvation-induced, hormone-like peptide secreted from the CC that promotes amino acid catabolism while limiting excessive depletion of energy stores during nutrient deprivation. Loss of CG14075/Mmt is associated with impaired degradation of threonine and BCAAs, together with enhanced mobilization of glycogen and TAGs (Fig. 6g). Together with AKH and Lst, our findings expand the repertoire of catabolic hormones secreted from the CC. AKH mobilizes lipids and carbohydrates as an early catabolic response, whereas the transcriptionally regulated lipase *bmm* promotes lipolysis as a late response in the fat body, acting in parallel with AKH^13^. The progressive increase in CG14075/Mmt secretion, particularly at mid-to-late stages of starvation, supports a temporally coordinated endocrine program rather than a simple linear relay. CG14075/Mmt appears to promote metabolic adaptation toward amino acid catabolism as starvation progresses, within a hormonal program that also includes AKH signalling^53^. These distinct temporal profiles of AKH and CG14075/Mmt likely help balance substrate utilization and sustain metabolic homeostasis during starvation. These results support the notion that the CC functions as a dynamic neuroendocrine hub that coordinates organismal catabolism through temporally and functionally distinct hormonal outputs.

Our data indicate that CG14075/Mmt broadly influences amino acid catabolism during starvation, with particularly prominent effects on threonine and BCAA metabolism. Disruption of these catabolic pathways, especially threonine metabolism, reduced starvation resistance and accelerated TAG depletion. Because both threonine and BCAAs are catabolized into TCA cycle intermediates such as acetyl-CoA and succinyl-CoA, they likely serve as important energy substrates under nutrient-deprived conditions. In *CG14075/Mmt* mutants, which fail to properly degrade these amino acids, excessive TAG mobilization may compensate for the reduced availability of amino acid-derived energy substrates. Interestingly, knockdown of the threonine catabolic enzyme Tdh led to accumulation not only of threonine but also of glycine, serine, and BCAAs. Although threonine catabolism normally contributes to glycine and serine production, its disruption would be expected to reduce these pools, particularly during nutrient limitation. Their accumulation instead suggests broader metabolic remodelling or compensatory activation of alternative pathways. These findings indicate that Tdh-dependent threonine catabolism influences systemic amino acid homeostasis more broadly than anticipated.

In addition to metabolic imbalance, impaired amino acid catabolism also affects redox homeostasis. *CG14075/Mmt* mutants and *Tdh* knockdown flies display increased oxidative stress, and antioxidant manipulations partially suppress the associated starvation-sensitive phenotype. Because threonine and BCAA catabolism occur primarily in mitochondria, impaired amino acid degradation could enhance mitochondrial ROS production. Recent studies also implicate BCAA catabolism in the synthesis of glutathione, a key antioxidant^53,55^. Moreover, reduced lipid droplet abundance in *CG14075/Mmt* mutants may further exacerbate oxidative damage, as lipid droplets have antioxidant properties^50^. Together, these observations support a model in which oxidative stress contributes to the link between impaired amino acid catabolism and starvation sensitivity. Nevertheless, the precise contribution of CG14075/Mmt-dependent threonine metabolism to starvation adaptation remains incompletely understood. Although perturbation of threonine catabolism by *Tdh* knockdown partially phenocopies the metabolic and oxidative stress phenotypes observed in *CG14075/Mmt* mutants, CG14075/Mmt loss affects not only threonine but also BCAAs and other essential and non-essential amino acids. Thus, Tdh-dependent threonine metabolism is likely to represent one component of a broader CG14075/Mmt-regulated amino acid metabolic programme, rather than the sole pathway responsible for starvation adaptation. Beyond supplying metabolic intermediates, amino acid catabolism could influence oxidative stress regulation and mitochondrial function. Future studies identifying the downstream signalling pathways of CG14075/Mmt, together with systematic analysis of how individual amino acid species are regulated and contribute to starvation responses, will be required to determine how amino acid metabolism is coordinated to support organismal survival during nutrient stress.

Hormonal regulation of amino acid metabolism remains relatively unexplored in *Drosophila*. In mammals, by contrast, glucagon secreted from pancreatic α-cells stimulates amino acid catabolism in the liver^56^. Other hormones, including cortisol and adrenaline, are also known to modulate metabolic adaptations to starvation^57,58^. Notably, in humans, glucagon, cortisol, and BCAAs all reach their peak levels ~2–3 days into fasting^5,59^, and during this phase, glucagon and cortisol act synergistically to regulate amino acid metabolism^41^. In flies, our results suggest that AKH and CG14075/Mmt represent distinct but interconnected hormonal systems that together shape metabolic adaptation to starvation. Our data suggest a functional interaction between CG14075/Mmt and AKH signalling during starvation. Loss of CG14075/Mmt was associated with increased AKH secretion, whereas inhibition of AKH signalling was accompanied by elevated circulating CG14075/Mmt levels. These observations raise the possibility that the two hormonal pathways are functionally coupled, either directly or indirectly, to shape metabolic responses during starvation. However, the mechanisms underlying this relationship remain unresolved. In particular, the increase in circulating CG14075/Mmt following fat body *AkhR* knockdown is consistent with the possibility that peripheral AKH/AkhR signalling influences CG14075/Mmt levels, but does not establish a direct feedback pathway from the fat body to the CC. One potential indirect component of such regulation may involve amino acids themselves. Recent work has shown that threonine can activate CC cells in *Drosophila* larvae^60^. It is therefore conceivable that amino acids that accumulate upon inhibition of CG14075/Mmt or AKH signalling, such as threonine and BCAAs, could modulate CC activity in adults and thereby contribute to changes in hormone secretion^53^. Although this model remains to be tested directly, it provides a potential mechanism by which nutrient-derived metabolic signals could influence CC endocrine output. This functional relationship between the two hormones could reflect their distinct roles in different phases of starvation, with AKH driving rapid responses to acute energy demands and CG14075/Mmt contributing to the slower adaptation to prolonged nutrient depletion. Consistent with this idea, AKH is rapidly secreted in response to immediate energy demands, such as flight^61^, whereas CG14075/Mmt accumulates progressively in the haemolymph as stored nutrients are depleted. Interestingly, CG14075/Mmt and AKH appeared to show different subcellular distributions within CC cells. This observation raises the possibility that the two hormones are differentially organised within CC cells, which may contribute to their distinct secretion dynamics and physiological functions during starvation. Elucidating the upstream cues and vesicle trafficking mechanisms that regulate starvation-induced secretion of CG14075/Mmt and AKH will therefore be critical for understanding how the CC orchestrates metabolic transitions during nutrient deprivation.

CG14075, recently named Marmite, was originally identified for its role in regulating yeast-specific feeding behaviour in a pair of brain neurons^32^. Our analysis revealed that CG14075/Mmt is also expressed in the CC. Interestingly, while neuronal CG14075/Mmt controls yeast preference, targeted knockdown in the CC selectively promoted sucrose consumption in starved flies without affecting yeast intake. This behavioural shift is consistent with the starvation-associated accumulation of amino acids and depletion of lipid and carbohydrate stores, suggesting a compensatory response to restore energy balance. These findings suggest that brain- and CC-derived CG14075/Mmt fulfill distinct physiological roles. Supporting this notion, the majority of CG14075/Mmt detected in the haemolymph originates from the CC. However, current annotations do not support distinct CG14075/Mmt isoforms, and our data do not determine whether brain- and CC-derived CG14075/Mmt differ at the molecular level. Nevertheless, tissue-specific peptide processing, post-translational modification, secretion dynamics, or local availability could contribute to their distinct physiological roles. Thus, the functional distinction between neuronal and CC-derived CG14075/Mmt may reflect not only the anatomical site of secretion and target tissue context, but also how the peptide is processed, secreted, or deployed in a tissue-specific manner. The phenomenon in which a single neuropeptide elicits functionally divergent outcomes depending on its site of secretion has been reported for certain gut hormones^21^. Such spatially contextualized deployment of a shared peptide signal across central and peripheral tissues likely represents an evolutionary strategy for maximizing signalling diversity with minimal genetic burden.

Finally, CG14075/Mmt shares structural similarity with mammalian NPB, particularly in the C-terminal region containing four conserved cysteine residues^32^. Synthetic peptides mimicking the predicted disulfide-linked C-terminal domain of CG14075/Mmt were sufficient to induce *Bcat* expression, supporting the functional importance of this conserved motif. Consistently, the C-terminal region alone can trigger amino acid catabolic gene expression, suggesting that CG14075/Mmt acts through a structurally defined, cysteine-rich motif, similar to many other peptide hormones^62^. Given that mammalian NPB binds to a specific class of G protein–coupled receptors (GPCRs)^63^, it is plausible that CG14075/Mmt also acts through a GPCR-like receptor expressed in peripheral tissues such as the fat body. However, we cannot exclude the possibility that CG14075/Mmt may also exert receptor-independent effects on peripheral tissues. For example, cysteine-rich peptides can influence cellular function through direct interactions with membranes or extracellular proteins^64,65^. In this context, CG14075/Mmt may regulate metabolic pathways not only via canonical receptor-mediated signalling but also through alternative mechanisms that do not require a dedicated receptor.

A major limitation of the present study is that the receptor, direct target tissues and downstream signalling pathways of CG14075/Mmt remain unidentified. Consequently, the mechanisms linking CC-derived CG14075/Mmt to starvation-induced changes in amino acid metabolism remain incompletely understood. In particular, we cannot distinguish whether the metabolic alterations observed in CG14075/Mmt mutants reflect direct actions of CG14075/Mmt on peripheral target tissues or secondary systemic responses to altered energy balance, feeding behaviour, amino acid accumulation, or AKH secretion. Future efforts to identify the receptor for CG14075/Mmt, define its tissue-specific sites of action, and characterize its downstream signalling cascades should help clarify how this peptide regulates amino acid metabolism in peripheral tissues during starvation.

## Methods

### Fly stocks and husbandry

Flies were raised on a standard fly food (8 g agar, 100 g glucose, 40 g dry yeast, 40 g corn flour, 4 mL propionic acid, and 3.5 mL 10% butyl p-hydroxybenzoate (in 100% ethanol) per litre) in 12:12 h light:dark conditions at 25 °C for 5–6 days before experiments. Genotypes that contained temperature-sensitive *Tub-GAL80ts* were raised at 20 °C from larvae to 3 days post-eclosion, after which they were incubated at 29 °C for 4 days to induce transgene expression before experiments began. For overexpression using *Tub-Gal4*^*ts*^ (the *Tub-Gal4, Tub-GAL80ts* line), flies were maintained at 20 °C for 4 days after eclosion and then shifted to 29 °C for 24 h to induce transgene expression.

The following transgenic and mutant stocks were used: *Akh-Gal4* (Bloomington *Drosophila* Stock Center [BDSC] #25684), *Akh-Gal4* (BDSC#25683), *UAS-LacZ*^*RNAi*^ (a gift from Masayuki Miura, the University of Tokyo), *UAS-mCherry*^*RNAi*^ (BDSC#35785), *UAS-Luciferase* (BDSC#35788), *UAS-CG14075*^*RNAiGD*^ (referred to as *CG14075*^*RNAi*^, Vienna *Drosophila* Resource Center [VDRC] #14238), *UAS-CG14075*^*RNAiKK*^ (VDRC#102202), *UAS-Akh*^*RNAi*^ (VDRC#105063), *UAS-Lst*^*RNAi*^ (VDRC#106861), *UAS-grim* (a gift from Dr. Michael B. O’Connor), *UAS-mCD8-GFP* (BDSC#32186), *Tub-Gal4* (BDSC#5138), *Tub-Gal80ts* (BDSC#7017), *Cg-Gal4* (BDSC#7011), *UAS-Tps1*^*RNAi*^ (NIG-FLY Stock Center [NIG] #4104R-6), *UAS-Treh*^*RNAi*^ (NIG#9364R-1), *UAS-bmm*^*RNAi*^ (BDSC#25926), *UAS-Bcat*^*RNAi*^ (BDSC#38363), *UAS-Gcat*^*RNAi*^ (NIG#HMC02902), *UAS-Tdh*^*RNAi*^ (NIG#5955R-3), *VT013947-Gal4* (VDRC#205650), *UAS-stinger* (BDSC#84277), *UAS-kir2.1* (BDSC#6596), *UAS-CG14075::HA* (This study), *UAS-CG14075-N* (This study), *UAS-CG14075-C* (This study), and *CG14075*^*KO*^ (This study).

*w*^*1118*^, *UAS-LacZ*^*RNAi*^, *UAS-mCherry*^*RNAi*^, and *UAS-Luciferase* were used as controls. CC ablation was performed by continuous expression of the *UAS-grim* transgene from the larval stage.

### *CG14075/Mmt* null mutant generation

We utilised a CRISPR/Cas9-mediated method^66,67^ to generate a knockout strain by inserting the 3xP3-DsRed cassette into the *CG14075/Mmt* locus. Approximately 500-bp sequences flanking the entire coding sequence of *CG14075/Mmt* were PCR amplified from the genomic DNA of the *w*^*1118*^ strain. The reporter cassette excised from pHD-DsRed (Addgene #51434), as well as the left and right homology arms, were assembled and cloned into SmaI-digested pBluescriptII SK(-) in a single enzymatic reaction using the In-Fusion Cloning Kit (TAKARA) to make a donor vector. gRNA vectors were constructed in pDCC6 (Addgene #59985). We selected two 20-bp gRNA target sequences that flank the start and end codons of *CG14075/Mmt*. To integrate a reporter cassette into the desired location in the genome, a mixture of a donor vector (150 ng/µL) and gRNA (150 ng/µL) vectors was injected into *w*^*1118*^ fertilized eggs. After crossing with a balancer strain, transformants in the F1 progeny were selected by eye-specific RFP expression from the 3×P3-DsRed marker gene in adults. Then, 3×P3-DsRed marker cassette was removed by crossing with the fly expressing Cre recombinase (BDSC#1092). The generated *CG14075/Mmt* null mutant was backcrossed six times with *w*^*1118*^. The primers used in the generation of *CG14075/Mmt* null mutant are listed in Supplementary Data 1.

### Generation of transgenic flies

For the generation of HA-tagged CG14075/Mmt-expressing plasmids, pUASTattB vector containing *CG14075/Mmt* coding sequence region (CDS) with an HA-encoding sequence at the C-terminus was synthesized by VectorBuilder.

To obtain fragmented CG14075/Mmt-expressing UAS-plasmids, the *CG14075/Mmt* CDS was amplified by PCR with adult *w*^*1118*^ whole-body cDNA using the primers UAS-CG14075_forward_EcoR1 and UAS-CG14075-N-fragment_R_Xba1. Following digestion with *EcoR*I (TAKARA) and *Xba*I (TAKARA), the N-terminal sequence of *CG14075/Mmt* was ligated with *EcoR*I-*Xba*I-digested pWALIUM10-moe vector (*Drosophila* Genomics Resource Center). The C-terminal fragment of *CG14075/Mmt* with the nucleotides from position 85 to 327 of the CDS removed was synthesized by IDT Inc. This fragment was then amplified by PCR using primers UAS-CG14075-C-fragment_F and UAS-CG14075-C-fragment_R, digested with *EcoR*I and *Xba*I, and subsequently cloned into the vector. Those vectors were then injected into fly embryos carrying *P{y[+t7.7]=CaryP}attP40* by WellGenetics. The primers used are listed in Supplementary Data 1.

### Quantitative reverse transcription PCR

To quantify changes in gene expression, whole bodies from three adult flies, fly abdominal carcasses from six adult flies, and head-anterior gut complexes from five adult flies were dissected for each sample. Total RNA was extracted using TRIzol (Thermo Fisher Scientific). cDNA was prepared with PrimeScript RT reagent Kit with gDNA Eraser (TAKARA). Quantitative reverse transcription PCR (RT-qPCR) was performed using ABI PRISM 7500 (Thermo Fisher Scientific) using TB Green Premix Ex TaqII (Takara Bio). Transcript levels of the target mRNA were normalized to *rp49* (*RpL32*) levels in the same samples. Primers used to measure transcript levels are listed in Supplementary Data 1.

For the validation of the RNA-seq data in Fig. 1, qPCR was performed using head–anterior gut complex samples. The qPCR primers used for the validation are listed in Supplementary Data 2.

### RNA-seq

For sampling CC containing neuronal ganglia, *Akh* > *mCD8-GFP* fly tissues were dissected in Schneider’s *Drosophila* Medium (Thermo Fisher Scientific, #21720024) under a fluorescent microscope. RNA was extracted from ten CC-containing neuronal ganglia and whole-body samples using TRIzol (Thermo Fisher Scientific). RNA was purified by NEBNext Poly(A) mRNA Magnetic Isolation Module (New England Biolabs [NEB], #E7490), followed by the NEBNext Ultra Directional RNA Library Prep Kit for Illumina (NEB, #E7420). The samples were quantified by Agilent Bioanalyzer with Agilent High Sensitivity DNA kit (Agilent, #5067-4626). Sequencing was performed using a NextSeq 500 (Illumina) with the High Output Kit v2. Expression analysis of the RNA sequencing data was performed using the CLC Genomics Workbench software (Qiagen). Reads were mapped to the BDGP6 reference with the RNA-Seq Analysis tool, and RPKM values were calculated. The RNA-seq read counts and related data are provided in Supplementary Data 3. Genes that showed higher expression in the CC compared to whole-body samples were curated and subsequently selected as targets for qRT-PCR analysis. The RNA-seq transcriptional data obtained from each genotype used for Fig. 1 is available from DNA Data Bank of Japan Sequence Read Archive (Accession number PRJDB35995).

### Feeding assays

CAFÉ assays were conducted as follows^36^. For each biological replicate, four adult flies were placed in a vial. Two calibrated glass micropipettes (5 μL; Drummond Scientific Company, #2-000-001-90) were filled by capillary action with liquid medium containing 5% sucrose and 2.5% autolysed yeast extract, and inserted through the sponge cap. Flies were allowed to feed for 24 h at 25 °C and 60% relative humidity. Evaporation was corrected by subtracting the volume change measured in identical CAFÉ chambers without flies. Liquid medium consumption was measured manually and divided by four to obtain μL/fly/h. For adult-specific knockdown experiments, flies were starved for 6 h and then assayed for 18 h at 29 °C.

FlyPAD assays were performed using the FlyPAD system^37^ as follows. A single fly starved in 1% agar for 16 h was placed in an arena with either 5% sucrose or 2.5% yeast autolysate solution, each mixed with 1% agar on a patch. In all experiments, flies were transferred to the FlyPAD arena under light anaesthesia and fed for 1 h at 25 °C and 60% relative humidity. The total number of sips per fly was calculated using FlyPAD algorithms.

### Starvation analysis

Ten 6–7-day-old adult flies were transferred into vials containing 2 mL of 1% agar (in dH₂O). Dead animals were counted every 12 h. Statistical significance was assessed using the log-rank test or pairwise log-rank test in R.

For antioxidant supplementation experiments, adult flies were maintained for 3 days on standard food with or without 20 mM L(+)-ascorbic acid sodium salt (FUJIFILM-Wako #198-01251), and starvation assays were subsequently performed as described above.

### Sleep and activity analysis

Sleep assays were carried out in a 12:12 h light:dark cycle at 25 °C using the DAM system (Trikinetics: http://www.trikinetics.com/). Sleep was analysed for 3 days starting from the day after the flies were loaded. Flies were fed 5% sucrose+2% agar. After the second light cycle, flies were transferred into the vial containing 2% agar without any sugars for starvation. Flies were considered asleep when they did not move for at least 5 min. Sleep time and activity were analysed by insomniac3^68,69^.

### Immunohistochemistry and fluorescence quantification

Fly tissues were dissected in 1× PBS and fixed in 4% formaldehyde in PBS for 30–60 min at room temperature (RT). Fixed samples were washed three times in PBS supplemented with 0.1% Triton X-100 (0.1% PBT). Samples were blocked in blocking solution (PBS with 0.1% Triton X-100 and 2% bovine serum albumin [BSA]) for 1 h at RT and then incubated with a primary antibody in the blocking solution at 4 °C overnight. Primary antibodies used in this study were chicken anti-GFP (1:4000, Abcam, #ab13970), guinea pig anti-CG14075/Mmt (1:10,000, this study), and mouse anti-AKH (1:200^70^). After washing, fluorophore (Alexa Fluor 488, 546, 555, or 633)-conjugated secondary antibodies (Thermo Fisher Scientific) and phalloidin dye were used at a 1:500 dilution, and samples were incubated for 2 h at RT in blocking solution.

For lipid droplet staining, the fly abdominal carcasses containing the fat body were stained with LipidTOX (Thermo Fisher Scientific #H34476; 1:500 in 0.1% PBT) for 2 h at RT after fixation.

Samples were visualized using a Zeiss LSM 800 confocal microscope. High-resolution images shown in Fig. 3i were acquired using a Zeiss LSM 990 confocal microscope equipped with Airyscan. Images were processed using Fiji. Fluorescence intensities in confocal sections were measured with Fiji version 2.16.0 (ImageJ), and the data were processed with Excel version 16.101, R version 4.4.2 and RStudio version 2024.12.0. Sum-intensity projections were generated to measure the total fluorescence intensity across the object of interest.

For line plot analysis, a line was manually drawn across the CC using Fiji, and fluorescence intensities of CG14075/Mmt and AKH were measured along the line. The resulting intensity profiles were exported and plotted using R.

For lipid droplet quantification, images corresponding to focal planes containing nuclei of fat body cells were selected. Lipid droplet area was measured using Fiji. The quantified areas were grouped by genotype and feeding condition and visualized using R.

### Measurement of protein, TAG, and sugar levels

Three flies from each group were homogenized using a pellet pestle with 300 μL of cold PBS containing 0.1% Triton X-100 and heated at 80 °C for 10 min. The supernatant was collected after centrifugation at 10,000 × *g* for 5 min at 4 °C. Six μL of supernatant was used for protein quantification with a BCA protein assay kit (Thermo Fisher Scientific, #23225). To measure whole-body triglycerides, we processed six μL of supernatant using a Serum Triglyceride Determination kit (Sigma-Aldrich, TR0100).

For sugar levels, six μL of the supernatant was mixed with 10 μL of PBS containing amyloglucosidase (Roche, #10102857001) or bacterially produced recombinant His-tagged cTreh^71^. Six μL of the supernatant was mixed with 10 μL of PBS without any enzymes in parallel for the determination of glucose levels. The reaction was carried out in a 16 μL assay mixture. The amount of glucose was determined by a glucose assay kit (Sigma-Aldrich, #GAGO20). The trehalose and glycogen concentrations for each sample were determined by subtracting the values of free glucose in the untreated samples. The amounts of TAG, trehalose, glycogen, and glucose were normalized to the total protein level.

### Antibody preparation

An antibody against CG14075/Mmt protein was raised in guinea pigs by Biogate Inc. A KLH-conjugated synthetic peptide (N-GAENEAKSKVGFPIDLDDG-C) corresponding to the CG14075/Mmt amino acid residues (GenBank accession number NP_649080.1) was used for immunization.

### CG14075/Mmt secretion assay with mammalian cells

HEK293T cells were obtained from the American Type Culture Collection. The cells were cultured in Dulbecco’s Modified Eagle Medium (DMEM, High Glucose; FUJIFILM-Wako) supplemented with 5% fetal bovine serum (FBS), 100 units/ml penicillin, and 100 μg/ml streptomycin (FUJIFILM-Wako) at 37 °C with 5% CO₂. For transfection, cells were seeded at a density of 5.0–7.5 × 10⁴ cells/cm² and incubated for 24 h. Plasmids were diluted in Opti-MEM (Thermo Fisher Scientific), and 1.0 μg/μL of polyethyleneimine MAX (PEI MAX; Polysciences) solution was also mixed with Opti-MEM. The plasmids and PEI solutions were then combined and incubated at room temperature for 20 minutes. The ratio of plasmid (μg) to PEI (μL) was 1:4. The mixture was subsequently added to the cells and incubated for 1 hour. After incubation, the medium was replaced with fresh culture medium (DMEM with 5% FBS and 1x PS), and the cells were incubated for an additional 18–24 h.

The cells expressing CG14075::HA and their culture media were processed with 2× sample buffer containing cOmplete protease inhibitor (Roche) after collection and subsequently used for Western blotting. The CG14075::HA-expressing plasmid was constructed as follows: after digestion with EcoRI (TAKARA) and XbaI (TAKARA), the full-length HA-tagged *CG14075/Mmt* fragment was ligated into the EcoRI–XbaI-digested pcDNA3 vector.

### Oxidative stress analysis

Ten flies were placed in vials containing 5 mM or 10 mM paraquat + 5% sucrose + 1% agar. Vials were stored at 25 °C and survival counts were taken every other day. Log-rank test or pairwise log-rank test was used to assess statistical significance using R.

Starvation assays with or without paraquat treatment were carried out under a 12:12 h light:dark cycle at 25 °C using the DAM system. Fly activity data were analysed until all flies were dead. Flies were fed 5 mM paraquat + 2% agar or 2% agar solution. The activity data were analysed by insomniac3^68,69^.

### CellROX staining

Prior to the experiment, 5-day-old flies were fed or placed in 1% agar for 36–48 h. The abdominal carcasses of the flies were dissected in Schneider’s *Drosophila* Medium, placed in 100 μL of Schneider’s *Drosophila* Medium containing 1:500 CellROX (Thermo Fisher Scientific #C10444), and incubated for 30 min at RT. The samples were rinsed twice with 1xPBS, and fixed in 4% PFA/PBS for 30 min. Then, samples were washed and incubated in 0.1% PBT containing Alexa Fluor 555–conjugated phalloidin and DAPI for 30 min. After staining, samples were mounted on glass slides and observed using a fluorescence microscope.

For CellROX intensity quantification, regions of interest (ROIs) were manually defined in Fiji based on DAPI-stained nuclei. CellROX fluorescence intensity was measured within each ROI, and the mean intensity per abdominal carcass was calculated for each sample. The resulting data were visualized using R.

### Ex vivo culture

The abdominal carcasses of the flies were dissected in Schneider’s *Drosophila* Medium and placed in 150 μL of Schneider’s *Drosophila* Medium, supplemented with fetal bovine serum (Gibco, #12483) and penicillin/streptomycin (Nacalai Tesque, #26253-84), with/without CG14075/Mmt synthetic peptide. The CG14075/Mmt synthetic peptide with the following sequence was synthesized by Peptide Institute Inc.; NH_2_-SAHGHRTCHVEIVSKVQGICQPMPIGSACVSDDYMDLYTDANC-COOH with disulfide bonds at C^8^-C^43^ and C^20^-C^29^. The samples were incubated for 2 h at RT on orbital rotation. After incubation, samples were processed for RNA extraction.

### Western blot

Three adult flies were homogenized in 100 μL of 1× SDS sample buffer containing cOmplete protease inhibitor cocktail (Roche) and phosphatase inhibitor cocktail (FUJIFILM-Wako) using a pellet pestle and boiled for 5 min.

For haemolymph extractions, 40 female flies were perforated with a tungsten needle and placed in a 0.5 mL tube perforated with a 27 G needle. The tubes were placed inside 1.5 mL tubes and centrifuged for 5 min at 5,000 × *g* at 4 °C to collect haemolymph. A 1 μL aliquot of the collected haemolymph was diluted in 9 μL of 1× SDS sample buffer containing cOmplete protease inhibitor cocktail (Roche) and boiled for 5 min. The samples were centrifuged at 20,000 × *g* for 5 min at RT, and the supernatants were subjected to SDS–PAGE followed by Western blotting using a PVDF membrane (Immobilon-P, Millipore). Primary and secondary antibodies and their dilutions were mouse anti-HA (1:1,000, Cell Signaling Technology, #2367), guinea pig anti-CG14075 antibody (1:10,000, This study), rabbit anti-phospho-S6K (1:500, Cell Signaling Technology, #9209), rabbit anti-phospho-AMPKα (1:1000, Cell Signaling Technology, #2535), rabbit anti-total AMPKα (1:1,000, Cell Signaling Technology, #2532), rat anti-αTubulin (1:1,000, # MA1-80017, Thermo Fisher), HRP-linked anti-guinea pig IgG (1:10,000, Millipore, #AP193P), HRP-linked anti-rabbit IgG (1:10,000, Cell Signaling Technology, #7074), HRP-linked anti-mouse IgG (1:10,000, Cell Signaling Technology, #7076) and HRP-linked anti-rat IgG (1:10,000, Cell Signaling Technology, #7077) antibodies. The proteins were visualized using ECL Prime Western blotting detection reagent (Amersham) and detected by a LuminoGraph II imaging system (ATTO). Images were processed and analysed using Fiji software. For the refeeding experiments shown in Fig. 3g, flies were starved on 1% agar for 24 h and then refed for 6 h with agar-based diets containing 10% sucrose, 2% peptone, or 2% yeast extract, standard diet, or filter paper soaked with coconut oil (FUJIFILM Wako, #036-20905). Haemolymph was then collected and analysed by western blotting as described above.

### Metabolite extraction and amino acid profiling

Frozen samples in 1.5-mL plastic tubes were homogenized in 300 μL cold methanol with a φ3 mm zirconia bead using a freeze crusher (TAITEC) at 33.3 Hz for 2 min. Homogenates were mixed with 200 μL of methanol, 200 μL of H_2_O, and 200 μL of CHCl_3_ and vortexed for 20 min at room temperature. Samples were then centrifuged at 20,000 × *g* for 15 min at 4 °C. The insoluble pellets were used to quantify total protein using a BCA protein assay kit (ThermoFisher Scientific). Supernatants were mixed with 350 μL of H_2_O, vortexed for 10 min at room temperature, and centrifuged at 20,000 × *g* for 15 min at 4 °C. Aqueous phases were collected and dried down using a vacuum concentrator. The dried material was redissolved in 15 μL of acetonitrile and 15 μL of MTBSTFA + 1% TBDMCS (Invitrogen, #TS-48927) and incubated for 60 min at 60 °C to derivatize. Derivatized metabolites were analysed with an Agilent 7890B GC coupled to a 5977 A Mass Selective Detector (Agilent Technologies) using a DB-5MS + DG column (30 m × 0.25 mm, 0.25-μm film thickness; Agilent Technologies). GC–MS data were acquired in SIM mode using MassHunter GC/MS Acquisition B.07.04.2260 (Agilent Technologies). Peak areas of interest were analysed using MassHunter Quantitative Analysis version B.07.01 (Agilent Technologies). Metabolite signals in whole-body samples were normalized to the total protein level of the corresponding sample. Heatmaps were drawn in MetaboAnalyst 5.0 (https://www.metaboanalyst.ca/home.xhtml). For mass spectrometry-based metabolite analyses, sample sizes and the number of biologically independent samples are indicated in the corresponding figure panels and Source Data file. For whole-body amino acid and metabolite profiling, each biological replicate consisted of two adult female flies. For all mass spectrometry-based analyses, sample sizes indicate biologically independent samples. Technical replicates, if any, were used only to assess measurement reproducibility and were not treated as independent biological replicates for statistical analyses.

### Isotope tracing experiments

For the experiments shown in Fig. 4g, 24 h-starved flies were transferred to 1% agar supplemented with either 3 mM L-Leu-^15^N (Cambridge Isotope Laboratories, #NLM-142-1) + 1% agar or 3 mM L-Thr-^13^C_4_,^15^N (Sigma, #607770) + 1% agar for 24 h. For the experiments shown in Supplementary Fig. 9e, flies were starved for 48 h and then transferred to 1% agar supplemented with 3 mM L-Thr-^13^C_4_,^15^N (Sigma, #607770) for 6 h. Abdominal carcasses from four flies were dissected in 1× PBS and immediately transferred into 200 μL of ice-cold methanol. The samples were then used for metabolite extraction as described above.

### AKH measurement by LC-MS/MS

For haemolymph AKH measurements^53^, 40–50 flies were perforated with a tungsten needle and placed in a 0.5 mL tube perforated at the bottom with a 27 G needle. These tubes were placed inside 1.5 mL tubes and centrifuged at 5,000 × *g* for 5 min at 4 °C to collect 1 μL aliquot of haemolymph. A 5 μL aliquot of haemolymph was used per sample.

Frozen samples were mixed with 500 μL methanol, 200 μL H_2_O, and 200 μL CHCl_3_, vortexed for 20 min at room temperature, and centrifuged at 20,000 × *g* for 15 min at 4°C. The supernatant was mixed with 350 μL H_2_O, vortexed for 10 min, and centrifuged again. The aqueous phase was collected and dried down using a vacuum concentrator. Dried extracts were reconstituted in 15 μL of 10% methanol prior to LC-MS/MS.

Chromatography was performed on an ACQUITY UPLC BEH C18 column (50 ×  2.1 mm, 1.7 μm particles, Waters) using an ACQUITY UPLC H-Class System (Waters). Mass spectrometric analysis was performed using a Xevo TQD triple quadrupole mass spectrometer (Waters) with an electrospray ionization source in the positive ion mode. Peak areas were analysed using MassLynx 4.1 software (Waters). Multiple reaction monitoring (MRM) transitions were m/z 975.6 > 416.3 and 958.7. Parameters were optimized using synthetic mature AKH peptide: pQLTFSPDWa, where pQ is pyroglutamic acid and Wa is tryptophan carboxamide (GenScript Japan).

### Glutathione measurement by LC-MS/MS

Frozen samples in 1.5 mL tubes were homogenized in 300 µL cold methanol containing 0.2% formic acid with a φ3 mm zirconia bead using a freeze crusher (TAITEC) at 33.3 Hz for 2 min. The homogenates were mixed with 200 µL methanol, 200 µL H_2_O, and 200 µL CHCl_3_, vortexed for 20 min at room temperature, and centrifuged at 20,000 × *g* for 15 min. The supernatant was mixed with 350 µL H_2_O, vortexed for 10 min, and centrifuged again. The aqueous phase was dried using a vacuum concentrator. Dried extracts were reconstituted in 0.1% formic acid containing 5% methanol prior to LC-MS/MS. The insoluble pellets were washed with 70% ethanol, solubilized in 400 μL of 0.2 N NaOH, heat-denatured, and used for total protein quantification with a BCA Protein Assay Kit (Thermo Fisher Scientific).

Chromatography was performed on an ACQUITY HSS T3 column (100 × 2.1 mm, 1.8 µm particles, Waters) using an ACQUITY UPLC H-Class System (Waters). Mass spectrometric analysis was performed using a Xevo TQD triple quadrupole mass spectrometer (Waters) with an electrospray ionization source. Peak areas of target metabolites were quantified using MassLynx 4.1 software (Waters). Metabolite signals were normalized to the total protein level of the corresponding sample. For glutathione measurements, each biological replicate consisted of two adult female flies.

### Alignment of CG14075/Mmt-related proteins

Using BlastP (https://blast.ncbi.nlm.nih.gov/Blast.cgi), we searched for proteins related to CG14075/Mmt based on its four cysteine residues. In addition, informed by a previous study^32^, we performed a motif search with the Motif Search tool from the Kyoto University Bioinformatics Center (https://www.genome.jp/tools/motif/MOTIF2.html) to identify homologous proteins in Deuterostomia. From the obtained amino acid sequences, signal sequences were predicted with SignalP 6.0 (https://services.healthtech.dtu.dk/services/SignalP-6.0/), and the regions excluding the signal sequences were aligned using the Constraint-based Multiple Alignment Tool (https://www.ncbi.nlm.nih.gov/tools/cobalt/re_cobalt.cgi). The protein/gene names, species, accession numbers or database IDs, and amino acid sequences used for the alignment are provided in Supplementary Data 4.

### CG14075 structure modelling

The structure of CG14075 protein was predicted using AlphaFold2 ColabFold^45,46^, excluding the signal sequence portion (MAPLHLLLHCATTLLIANA). The cysteine residues were then visualized using VMD version 1.9.4 for the Rank 1 model.

### Statistics and reproducibility

All statistical analyses were carried out using “R”. All experiments were performed independently at least twice. All immunohistochemical and western blot experiments were repeated at least twice with similar results. In each experiment, we analysed three or more specimens. Experiments were not randomized, and investigators were not blinded. No statistical methods were used to predetermine sample sizes. Sample sizes were chosen based on prior experience, feasibility of the assays and sample sizes commonly used in previous studies with similar experimental designs. Comparable sample sizes were used across experimental groups where applicable.

### Manuscript preparation

During manuscript preparation, the authors used ChatGPT (OpenAI) to improve clarity and readability. All content was reviewed and edited by the authors, who take full responsibility for the final version of the manuscript.

### Reporting summary

Further information on research design is available in the Nature Portfolio Reporting Summary linked to this article.

## Supplementary information

Supplementary information

Description of Additional Supplementary Files

Supplementary Data 1-4

Reporting Summary

Transparent Peer Review file

## Source data

Source Data

## Data Availability

The RNA-seq data generated in this study have been deposited in the DNA Data Bank of Japan Sequence Read Archive under BioProject accession code PRJDB35995. The RNA-seq read counts and related data are provided in Supplementary Data 3. The *Drosophila melanogaster* BDGP6 reference genome used for RNA-seq read mapping is available under accession code GCA_000001215.4 [https://www.ncbi.nlm.nih.gov/datasets/genome/GCA_000001215.4/]. The previously published Fly Cell Atlas datasets used for single-cell expression visualization are available under accession codes E-MTAB-10519 and E-MTAB-10628. Primer information is provided in Supplementary Data 1 and 2. Protein/gene names, species, accession numbers or database IDs, and amino acid sequences used for the CG14075/Mmt-related protein alignment are provided in Supplementary Data 4. No custom code was generated for this study. Source data are provided with this paper.
