# Supplementary material for: CG14075/Mmt promotes amino acid catabolism and cooperates with AKH during starvation in Drosophila
Source: Nat Commun. 2026 Sep 12;17:10766. doi: 10.1038/s41467-026-77687-7 (PMC13661902; doi:10.1038/s41467-026-77687-7)
Supplement: Supplementary file 1 [file 41467_2026_77687_MOESM1_ESM.pdf]

Supplementary Information

Supplementary Figures 1–12 and their legends

CG14075/Mmt promotes amino acid catabolism and cooperates with AKH during starvation in *Drosophila*

Yuto Yoshinari[1,2]*, Maiko Abe[3], Ryo Hoshino[2,3], Yosuke Mizuno[3], Yoshitomo Kurogi[3], Eisuke Imura[3,7], Akira Nakamura[4,5], Fuminori Tsuruta[6], Ryusuke Niwa[2]*, Takashi Nishimura[1]*

[1] Metabolic Regulation and Genetics, Institute for Molecular and Cellular Regulation, Gunma University, Maebashi, Gunma, Japan

[2] Life Science Center for Survival Dynamics, Tsukuba Advanced Research Alliance (TARA), University of Tsukuba, Tsukuba, Ibaraki, Japan

[3] Graduate School of Life and Environmental Sciences, University of Tsukuba, Tsukuba, Ibaraki, Japan

[4] Department of Germline Development, Institute of Molecular Embryology and Genetics, Kumamoto University, Kumamoto, Japan

[5] Graduate School of Pharmaceutical Sciences, Kumamoto University, Kumamoto, Japan

[6] Institute of Life and Environmental Sciences, University of Tsukuba, Tsukuba, Japan.

[7] Present address: Department of Entomology, Institute for Integrative Genome Biology, University of California, Riverside, 900 University Ave, Riverside, CA 92521, USA.

*Correspondence

Yuto Yoshinari (yuto.yoshinari@gunma-u.ac.jp)

Ryusuke Niwa (ryusuke-niwa@tara.tsukuba.ac.jp)

Takashi Nishimura (t-nishimura@gunma-u.ac.jp)

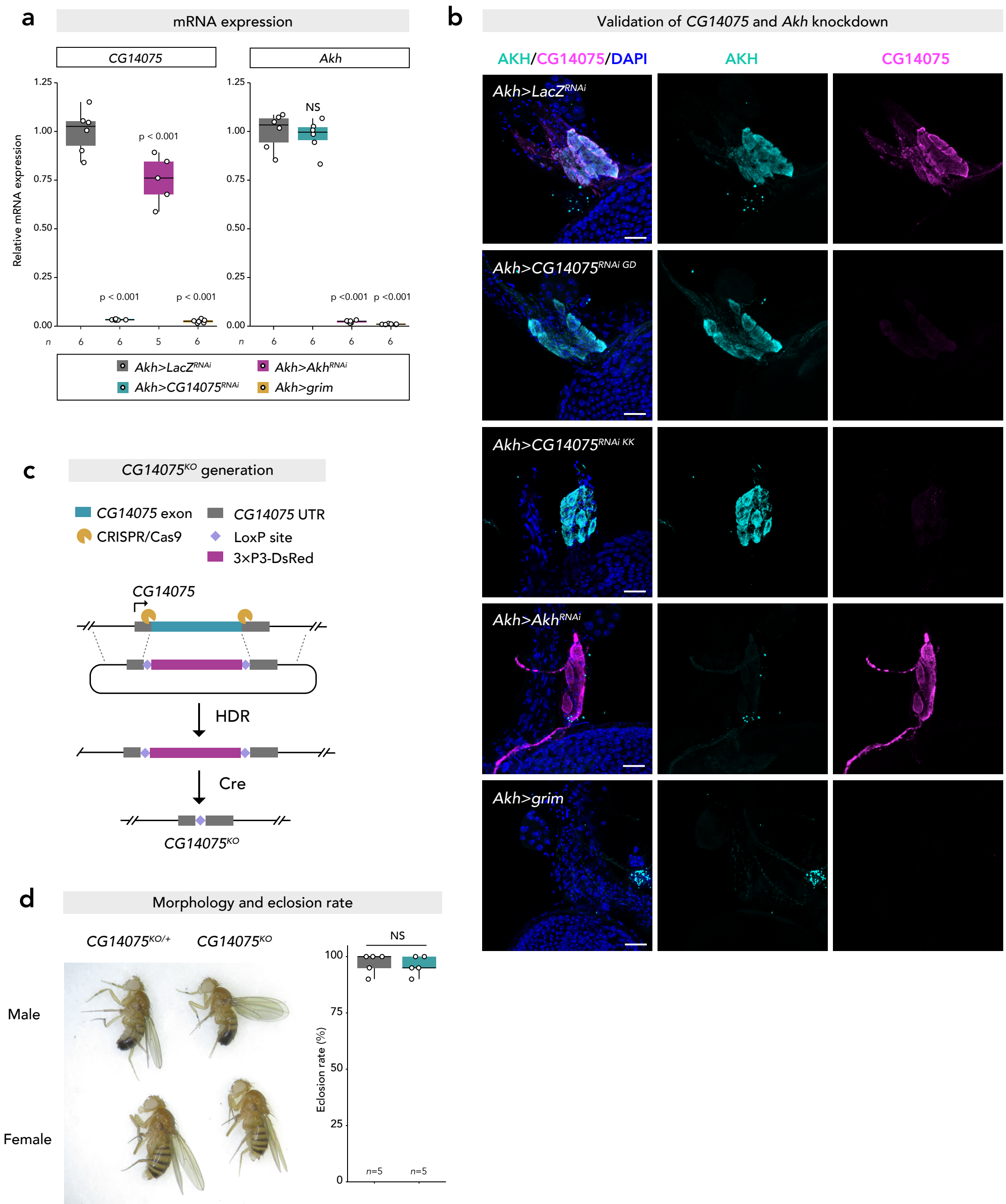

Supplementary Fig. 1 | Validation of *CG14075* RNAi and anti-CG14075 antibody.

a, Relative mRNA expression levels of *Akh* and *CG14075* in whole-body samples. **b**, Immunostaining of the CC labelled with anti-AKH (cyan) and anti-CG14075 (magenta) antibodies. Scale bars, 20 μm. Representative images from two independent experiments with similar results are shown. **c**, Schematic of generation of the *CG14075* null mutant. CRISPR–Cas9-mediated homologous recombination (homology-directed repair, HDR) was used to insert 3×P3-DsRed into the *CG14075* coding region, followed by removal of the cassette using Cre recombinase. **d**, Morphology and eclosion rate of control and *CG14075*^{KO}

flies.

Statistics: Unpaired two-tailed Student's t-test (a) and Wilcoxon rank-sum test (d). NS, not significant ($P > 0.05$). Box plots, centre line (median), box limits (first and third quartiles), and whiskers (most extreme data points within 1.5× the interquartile range). Sample sizes (n) are indicated in each panel.

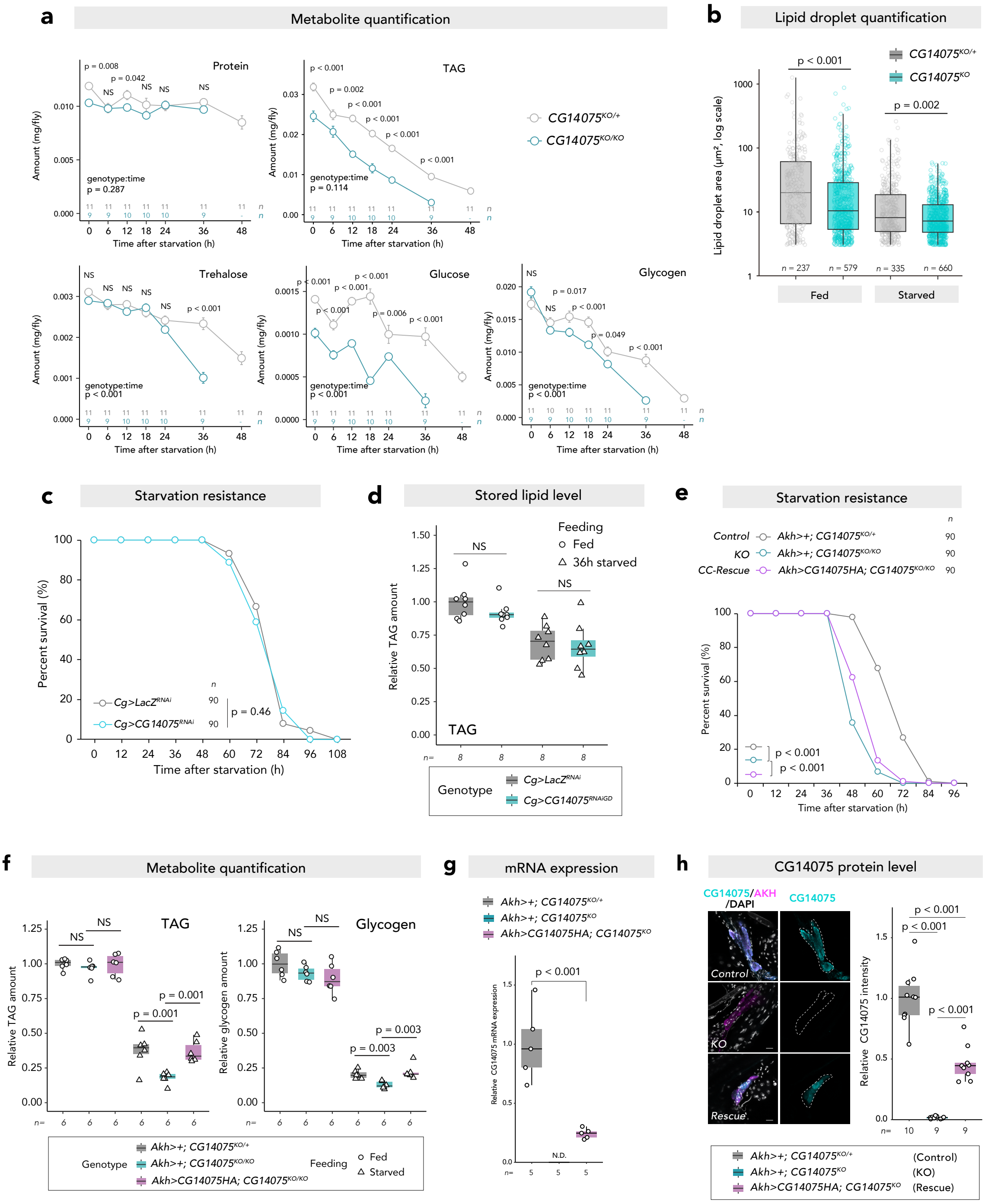

Supplementary Fig. 2 | *CG14075*^{KO} phenotypes are rescued by CG14075 reintroduction in the CC.

a, Whole-body protein, TAG, glycogen, trehalose and glucose levels in control and *CG14075*^{KO} female flies. Starvation-dependent time-course changes in each metabolite are shown. P values from two-way ANOVA (genotype, starvation time and their interaction) are indicated below each panel. **b**, Quantification of lipid droplet area in control and *CG14075*^{KO} flies, related to Fig. 2c. **c**, Starvation survival curves of control and adipose tissue-specific

CG14075 knockdown female flies. **d**, Whole-body TAG levels in flies under fed and starved conditions. **e**, Starvation survival curves of control, *CG14075*^{KO}, and CC-specific *CG14075* rescue flies. **f**, Whole-body TAG (left) and glycogen (right) levels in control, *CG14075*^{KO}, and CC-specific *CG14075* rescue flies under fed and starved conditions. **g**, *CG14075* mRNA expression levels in control, *CG14075*^{KO} and CC-specific *CG14075* rescue flies. **h**, Left, immunostaining of the CC labelled with anti-CG14075 (cyan) and anti-AKH (magenta) antibodies. Scale bars, 10 μm. Right, quantification of CG14075 signal intensity. Representative images from two independent experiments with similar results are shown.

Statistics: Unpaired two-tailed Student's t-test (a, d), two-way ANOVA for genotype and starvation time effects and their interaction (a), pairwise Wilcoxon rank-sum test (b, f; glycogen, h), log-rank test (c), pairwise log-rank test (e), and one-way ANOVA followed by Tukey's multiple comparisons test (f; TAG, g). NS, not significant ($P > 0.05$). Line graphs, mean ± SE. Box plots, centre line (median), box limits (first and third quartiles), and whiskers (most extreme data points within 1.5× the interquartile range). Sample sizes (n) are indicated in each panel.

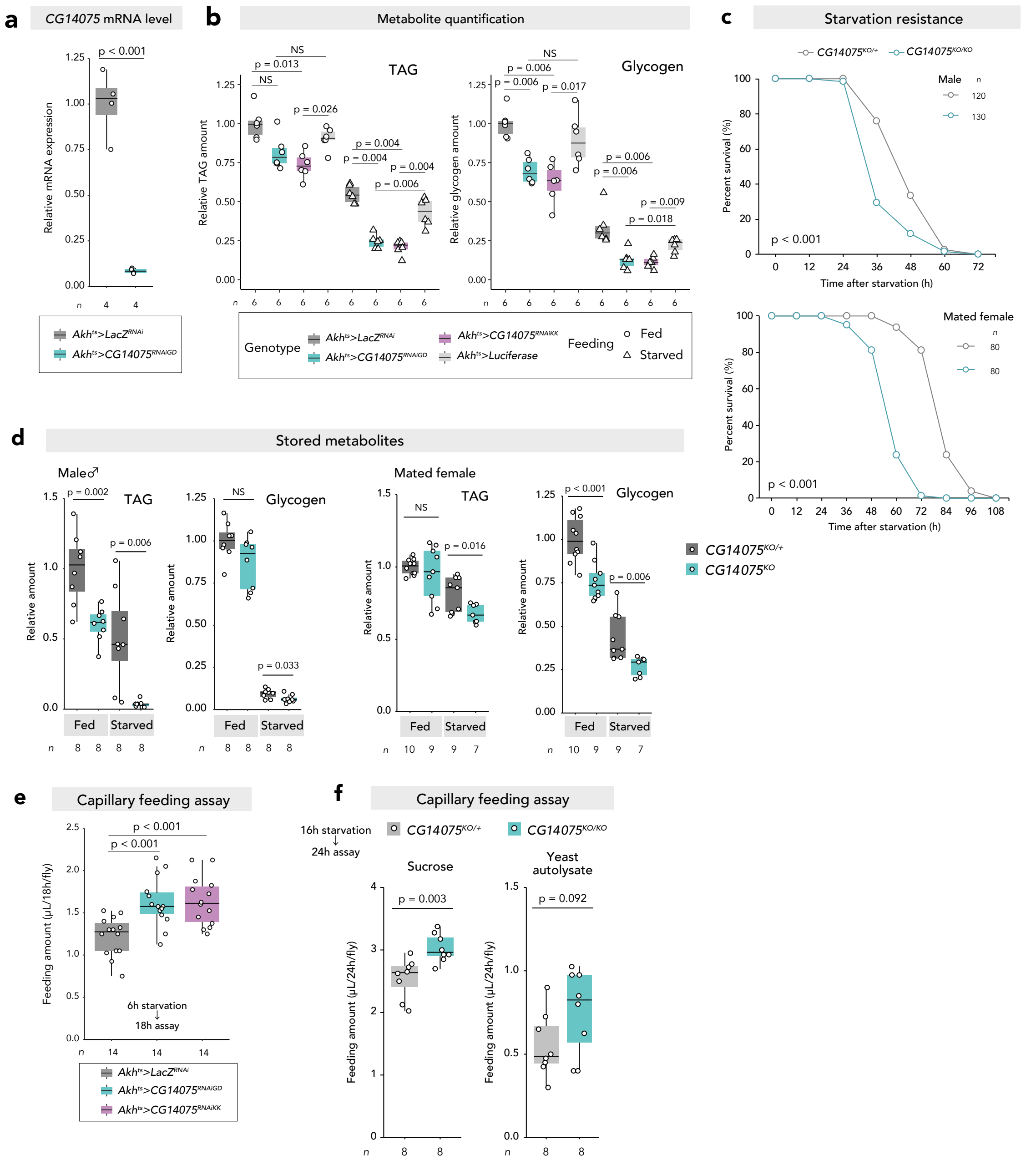

Supplementary Fig. 3 | Metabolic phenotypes of *CG14075* knockdown and knockout flies.

a, *CG14075* mRNA expression levels in adult-specific *CG14075* knockdown flies. **b**, Relative whole-body TAG (left) and glycogen (right) levels in control flies (*LacZ*^{RNAi} and *Luciferase*) and two independent *CG14075* RNAi lines under fed and starved conditions. **c**, Starvation survival curves of control and *CG14075*^{KO} male (top) and mated female (bottom) flies. **d**, Relative whole-body TAG (left) and glycogen (right) levels in 24 h-starved male and mated female control and *CG14075*^{KO} flies. **e**, Feeding measurements using an 18 h CAFÉ assay. Flies were starved for 6 h prior to the assay. Experiments were conducted at 29 °C. **f**, Feeding

measurements using the CAFÉ assay. Flies were starved for 16 h prior to the assay.
Statistics: Unpaired two-tailed Student's t-test (a, f), pairwise Wilcoxon rank-sum test (b), log-rank test (c), Welch's t-test (d), and one-way ANOVA followed by Tukey's multiple comparisons test (e). NS, not significant ($P > 0.05$). Box plots, centre line (median), box limits (first and third quartiles), and whiskers (most extreme data points within 1.5× the interquartile range). Sample sizes (n) are indicated in each panel.

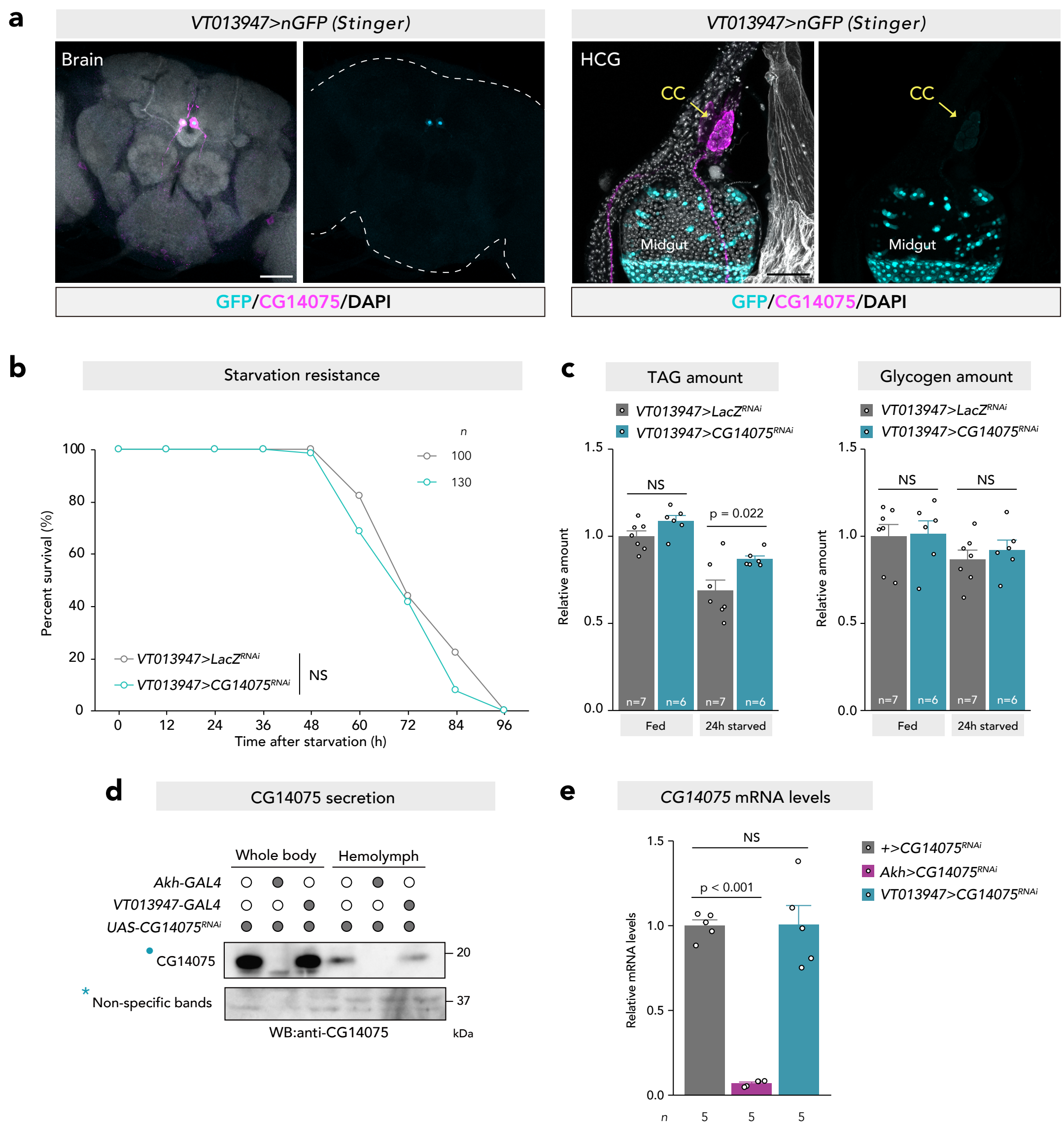

Supplementary Fig. 4 | Brain-derived CG14075 does not affect starvation resistance. **a**, Immunostaining of the brain (left) and hypocerebral ganglion (HCG) (right) labelled with anti-GFP (cyan) and anti-CG14075 (magenta) antibodies. DAPI (grey) labels DNA. Yellow arrows indicate the CC. Dashed lines indicate the outline of the brain. Scale bars, 20 µm. Representative images from two independent experiments with similar results are shown. **b**, Starvation survival curves of control and *VT013947>CG14075^RNAi* female flies. **c**, Relative whole-body TAG (left) and glycogen (right) levels in control and *VT013947>CG14075^RNAi* female flies. **d**, Western blot of CG14075 in control (*+>CG14075^RNAi*) and CC- or brain-specific *CG14075* knockdown flies. Blue dots and asterisks indicate CG14075-specific bands and non-specific bands (used as loading controls), respectively. **e**, Relative whole-body *CG14075* mRNA expression levels in control, CC-specific, and brain-specific knockdown flies. Statistics: Log-rank test (b), unpaired two-tailed Student's t-test (c), and one-way ANOVA

followed by Dunnett's multiple comparisons test (e). NS, not significant ($P > 0.05$). Bar plots, mean ± SE. Sample sizes (n) are indicated in each panel.

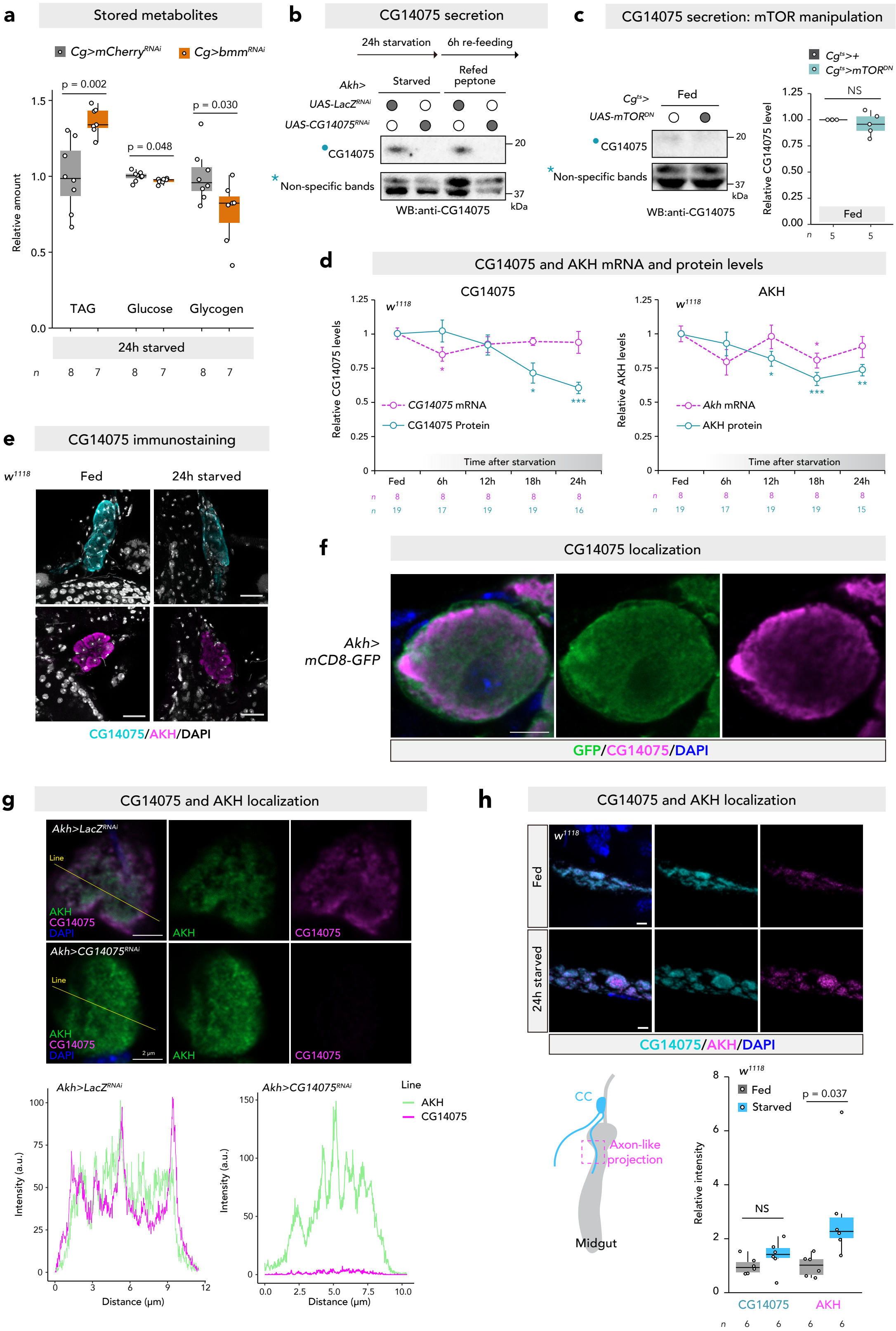

Supplementary Fig. 5 | CG14075 and AKH localization.

a, Relative whole-body TAG, glucose and glycogen levels in control and *Cg>bmm* ^{RNAi} female

flies under starvation. **b**, CG14075 secretion in response to refeeding. Flies were refed with each diet for 6 h following 24 h starvation. **c**, CG14075 secretion in control and dominant-negative mTOR–overexpressing flies. **d**, Time course of CG14075 and AKH mRNA and protein levels. **e**, Immunostaining of CC from fed or 24 h-starved *w*^{1118} flies labelled with anti-CG14075 antibody (cyan), anti-AKH antibody (magenta), and DAPI (grey). Scale bars, 20 μm. **f**, Immunostaining of CC from *Akh>mCD8-GFP* flies labelled with anti-GFP antibody (green), anti-CG14075 antibody (magenta), and DAPI (blue). Scale bars, 2 μm. **g**, Top, immunostaining of CC from control and CC-specific *CG14075* knockdown flies labelled with anti-CG14075 antibody (magenta), anti-AKH antibody (green), and DAPI (blue). Scale bars, 2 μm. Bottom, intensity profiles of CG14075 and AKH along the indicated lines. a.u.; arbitrary units. **h**, Top, immunostaining of axon-like projections from fed or 24 h-starved flies labelled with anti-CG14075 antibody (cyan), anti-AKH antibody (magenta), and DAPI (blue). Scale bars, 2 μm. Bottom, schematic of the CC and its axon-like projection to the midgut, and quantification of CG14075 and AKH signal intensity in these projections under fed or 24 h-starved conditions. Representative images from two independent experiments with similar results are shown in (e, f, g, h).

Statistics: Unpaired two-tailed Student's t-test (a, d, h) and Wilcoxon rank-sum test (c). *$P < 0.05$, **$P < 0.01$, and ***$P < 0.001$; NS, not significant ($P > 0.05$). Line graphs, mean ± SE. Box plots, centre line (median), box limits (first and third quartiles), and whiskers (most extreme data points within 1.5× the interquartile range). Sample sizes (n) are indicated in each panel.

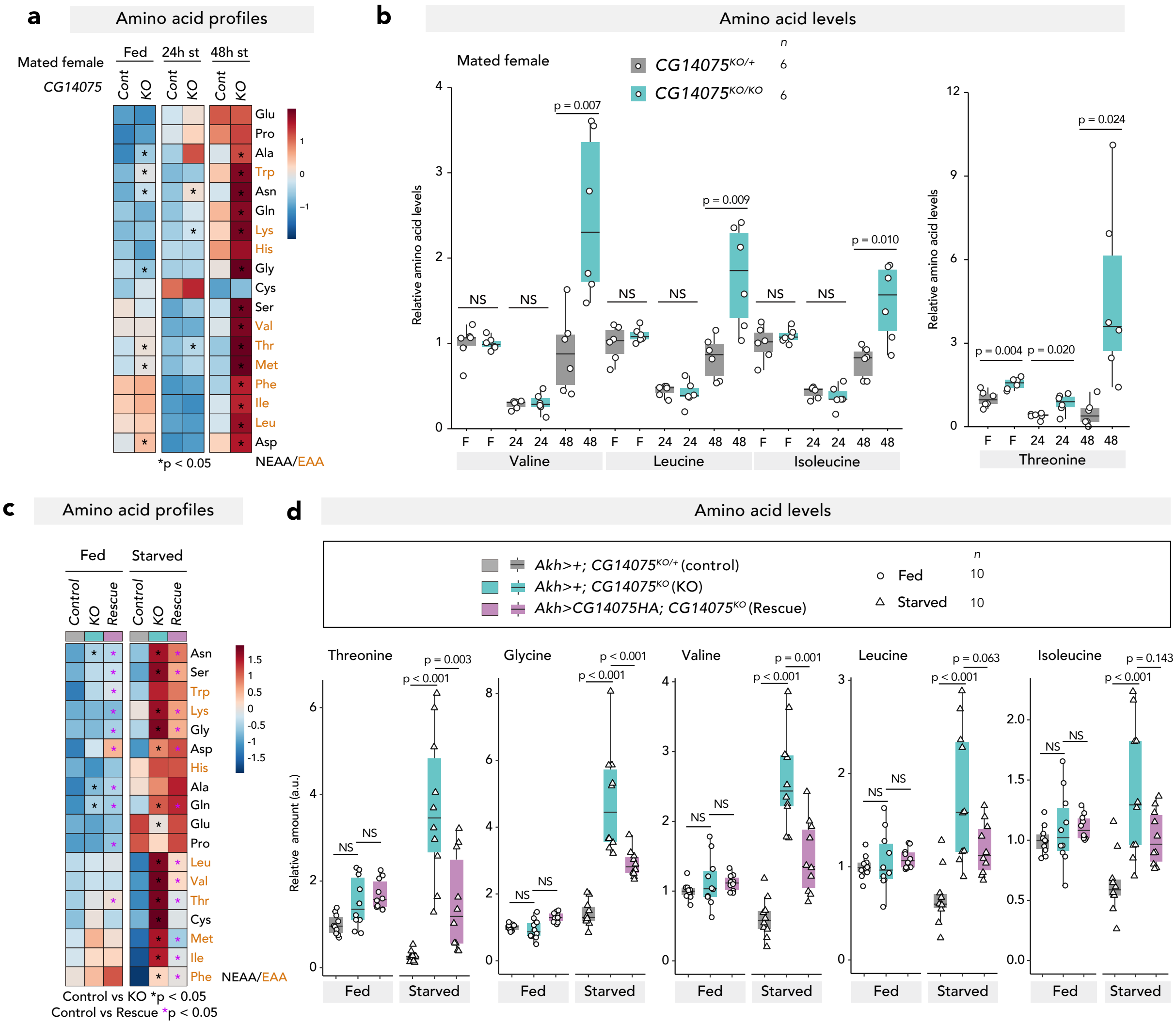

Supplementary Fig. 6 | Amino acid profiles of *CG14075*^{KO} and rescue flies.

a, Heatmap of amino acid profiles in control and *CG14075*^{KO} mated female flies. EAAs are highlighted in orange. Asterisks indicate statistical significance between control and *CG14075*^{KO} flies under fed, 24 h-starved and 48 h-starved conditions. **b**, Relative levels of BCAAs and threonine in flies as in (a). F, fed; 24, 24 h-starved; 48, 48 h-starved. **c**, Heatmap of amino acid profiles in control, *CG14075*^{KO}, and CC-specific *CG14075* rescue female flies. EAAs are highlighted in orange. Asterisks indicate statistically significant differences between control and *CG14075*^{KO} flies, and between control and rescue flies, under fed and starved conditions. **d**, Relative levels of threonine, glycine, and BCAAs in control, *CG14075*^{KO}, and CC-specific *CG14075* rescue female flies.

Statistics: Welch's t-test (a, b) and pairwise Wilcoxon rank-sum test (c, d). *P < 0.05; NS, not significant (P > 0.05). Box plots, centre line (median), box limits (first and third quartiles), and whiskers (most extreme data points within 1.5× the interquartile range). Sample sizes (n) are indicated in each panel.

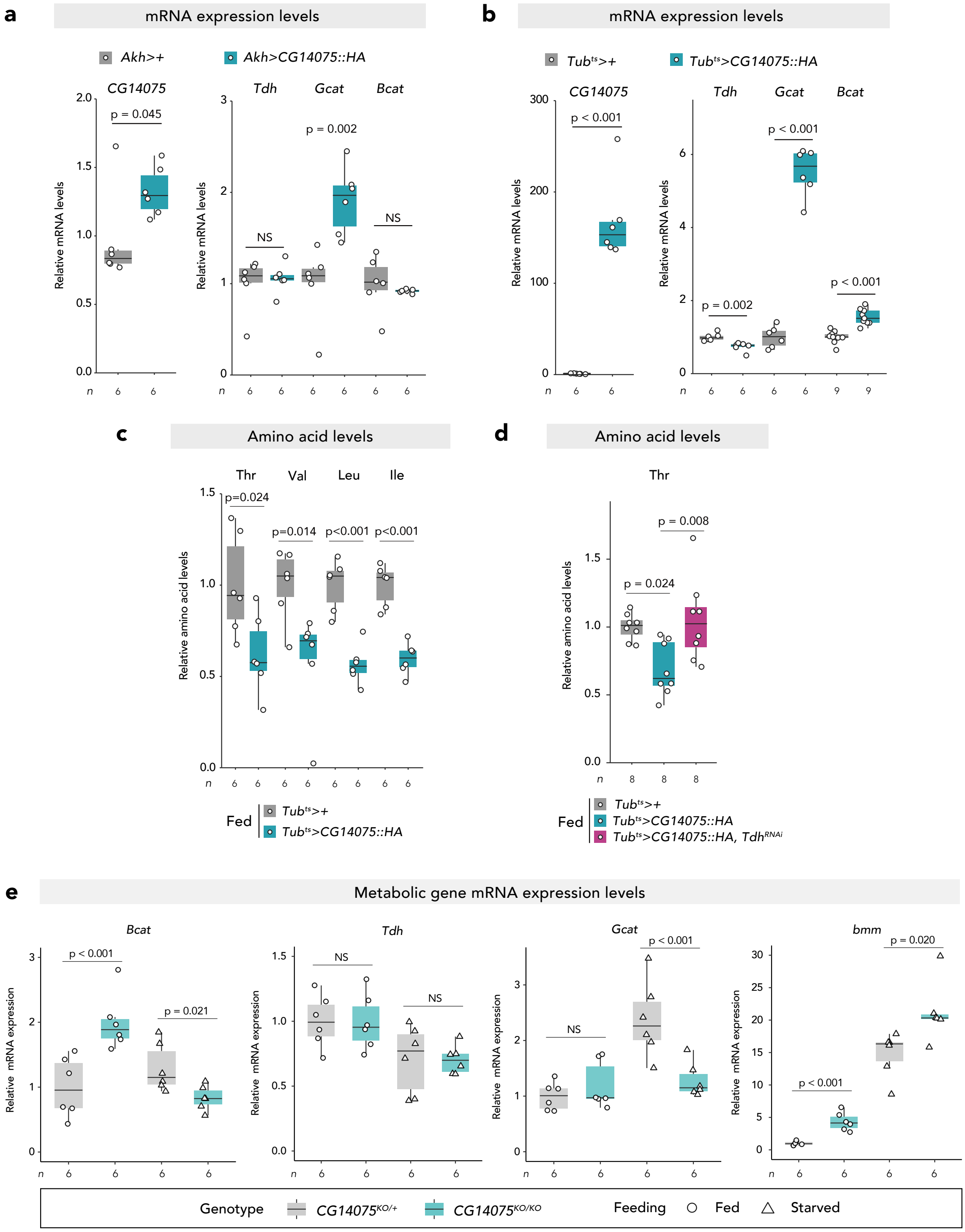

Supplementary Fig. 7 | CG14075 regulates amino acid catabolic gene expression.

a, Relative mRNA expression levels of *CG14075* in whole-body samples and of amino acid catabolic enzyme genes in abdominal carcasses from control and CC-specific *CG14075*-overexpressing flies. **b**, Relative mRNA expression levels of *CG14075* in whole-body samples and of amino acid catabolic enzyme genes in abdominal carcasses from control and

whole body *CG14075*-overexpressing flies. **c**, Relative levels of BCAAs and threonine in control and flies overexpressing *CG14075* for 24 h. **d**, Relative threonine levels in control, *CG14075*-overexpressing, and *CG14075*-overexpressing *Tdh* knockdown flies for 24 h. **e**, Relative mRNA expression levels of amino acid metabolic genes and the lipase gene *bmm* in the abdominal carcass of control and *CG14075*^{KO} flies.

Statistics: Unpaired two-tailed Student's t-test (a, b, c, e) and one-way ANOVA followed by Tukey's multiple comparisons test (d). NS, not significant ($P > 0.05$). Box plots, centre line (median), box limits (first and third quartiles), and whiskers (most extreme data points within 1.5× the interquartile range). Sample sizes (n) are indicated in each panel.

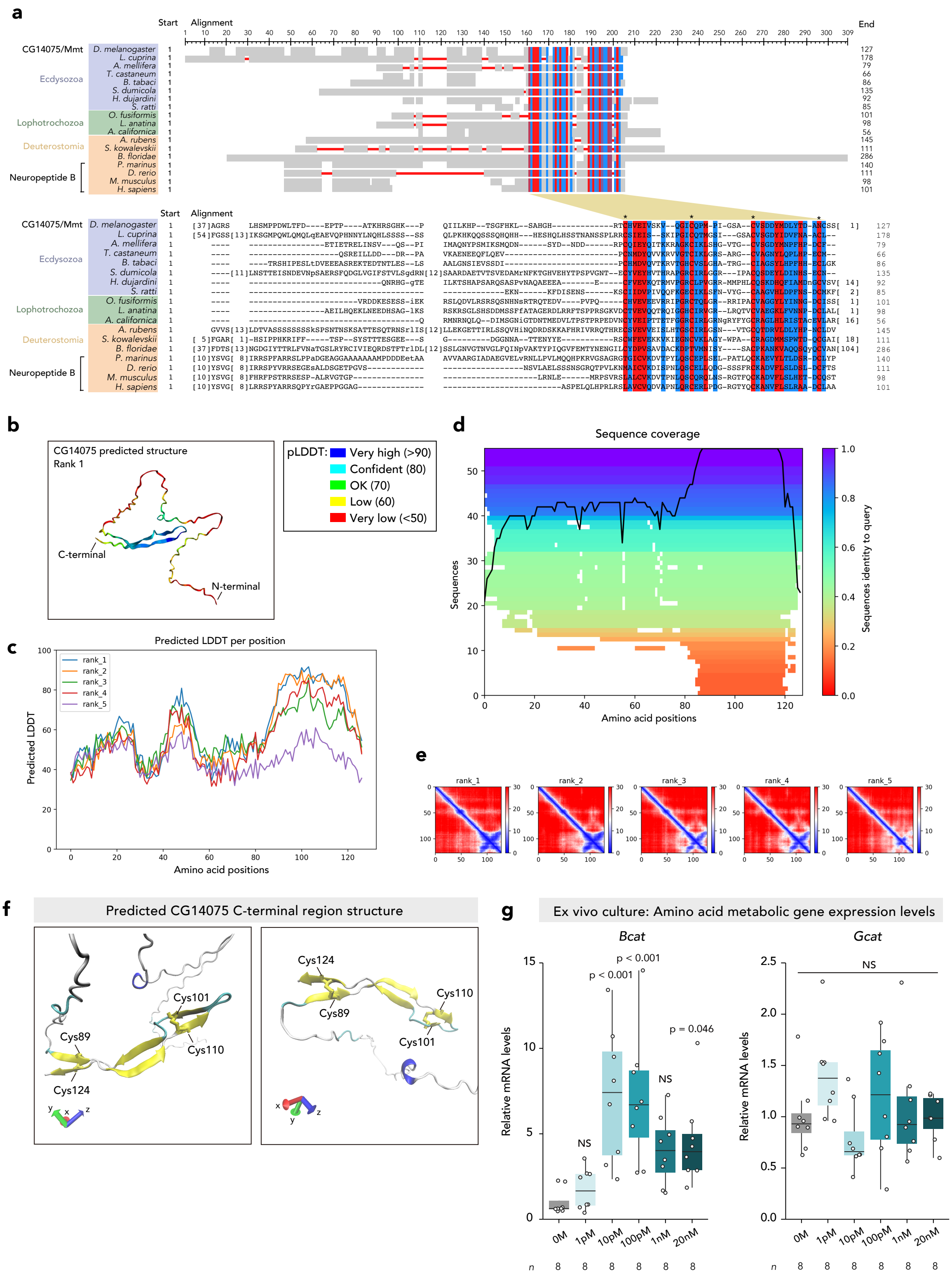

Supplementary Fig. 8 | Predicted structure of CG14075.

a, Multiple sequence alignment of CG14075-related proteins after removal of predicted signal sequences. Amino acid positions are coloured according to sequence conservation, with red indicating highly conserved positions and blue indicating lower conservation. Asterisks

indicate conserved cysteine residues. **b**, A Rank 1 predicted structure of CG14075 by AlphaFold2. The predicted local distance difference test (pLDDT) score indicates model confidence (out of 100) at each position using five color levels. **c**, Per-position pLDDT scores for each of the five predicted models. **d**, Sequence coverage plot illustrating the per-residue coverage, calculated as sequence identity to the query. Coverage represents the number of homologous sequences detected per residue and is indicated by the black line in the plot. **e**, The predicted aligned error (PAE) plots for the five models, showing computed error for each residue relative to all other residues. Note that low error scores are colored in blue, and high error scores are colored in red. **f**, The predicted CG14075 structures show the proximity of four cysteine residues. x, y, and z indicate axial directions; Left panel and right panel visualize the C-terminal region from different directions. **g**, mRNA expression profiles of *Bcat* and *Gcat* of dissected fly abdomens incubated with or without CG14075 synthetic peptide.

Statistics: One-way ANOVA followed by Dunnett's multiple comparisons test (g), NS, not significant ($P > 0.05$). Box plots, centre line (median), box limits (first and third quartiles), and whiskers (most extreme data points within 1.5× the interquartile range). Sample sizes (n) are indicated in each plot.

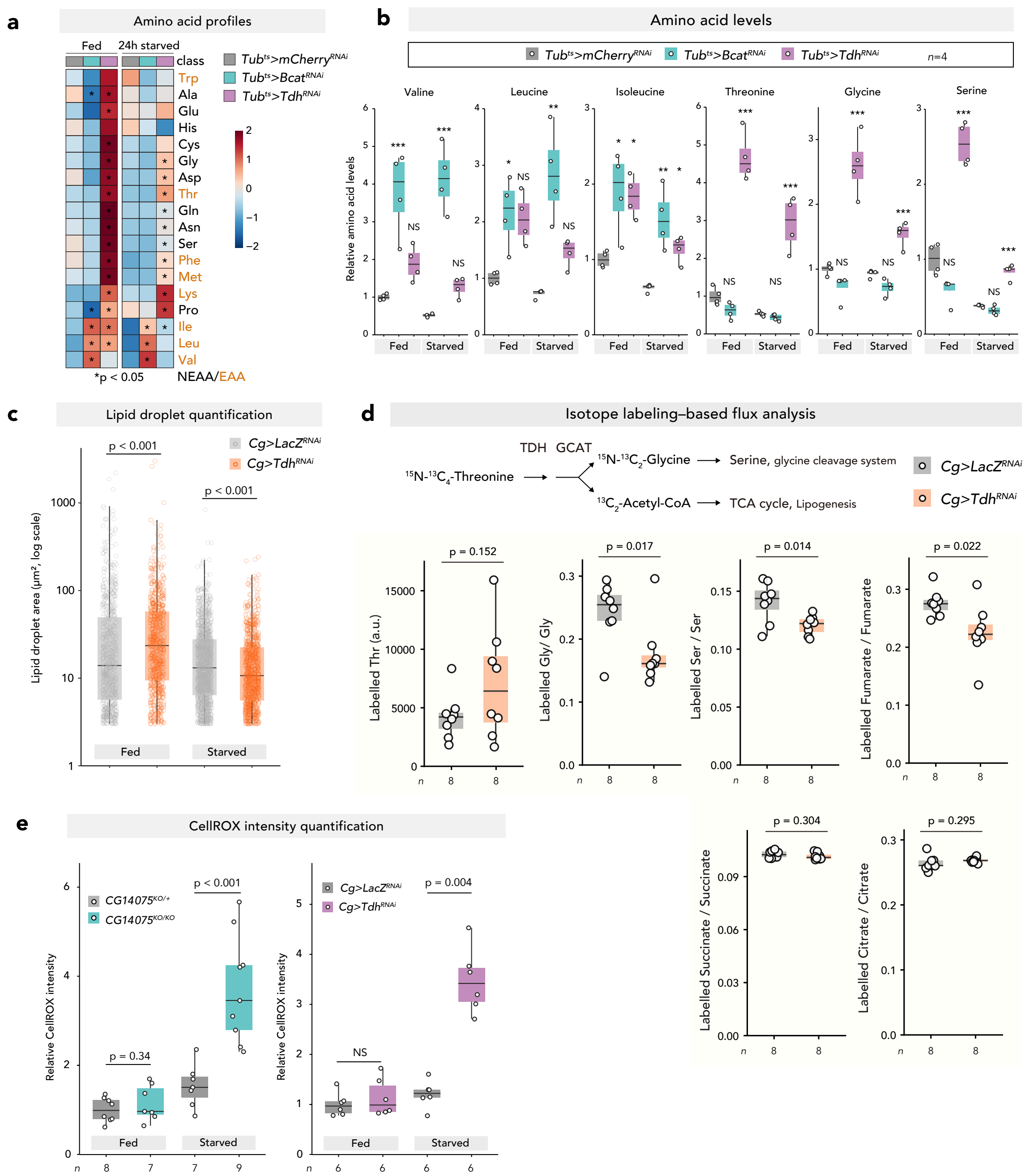

Supplementary Fig. 9 | Tdh regulates threonine catabolism.

a, Heatmap of amino acid profiles in control, *Bcat* knockdown, and *Tdh* knockdown female flies. EAAs are highlighted in orange. Asterisks indicate statistical significance between control and RNAi flies under fed and 24 h-starved conditions. **b**, Relative levels of BCAAs, threonine, glycine, and serine in control and RNAi flies. **c**, Quantification of lipid droplet area in control and *Tdh* knockdown flies, related to Fig. 5d. **d**, Isotope tracing experiments for *Tdh* knockdown flies. 48 h starved flies were fed with agar containing 3 mM ^{15}N-$^{13}C_4$-Thr for 6 h. **e**, Quantification of CellROX signal intensity, related to Fig. 5e.

Statistics: One-way ANOVA followed by Tukey's multiple comparisons test (a, b), Wilcoxon

rank-sum test (c, e), and Welch's t-test (d). *P < 0.05, **P < 0.01, and ***P < 0.001; NS, not significant (P > 0.05). Box plots, centre line (median), box limits (first and third quartiles), and whiskers (most extreme data points within 1.5× the interquartile range). Sample sizes (n) are indicated in each panel.

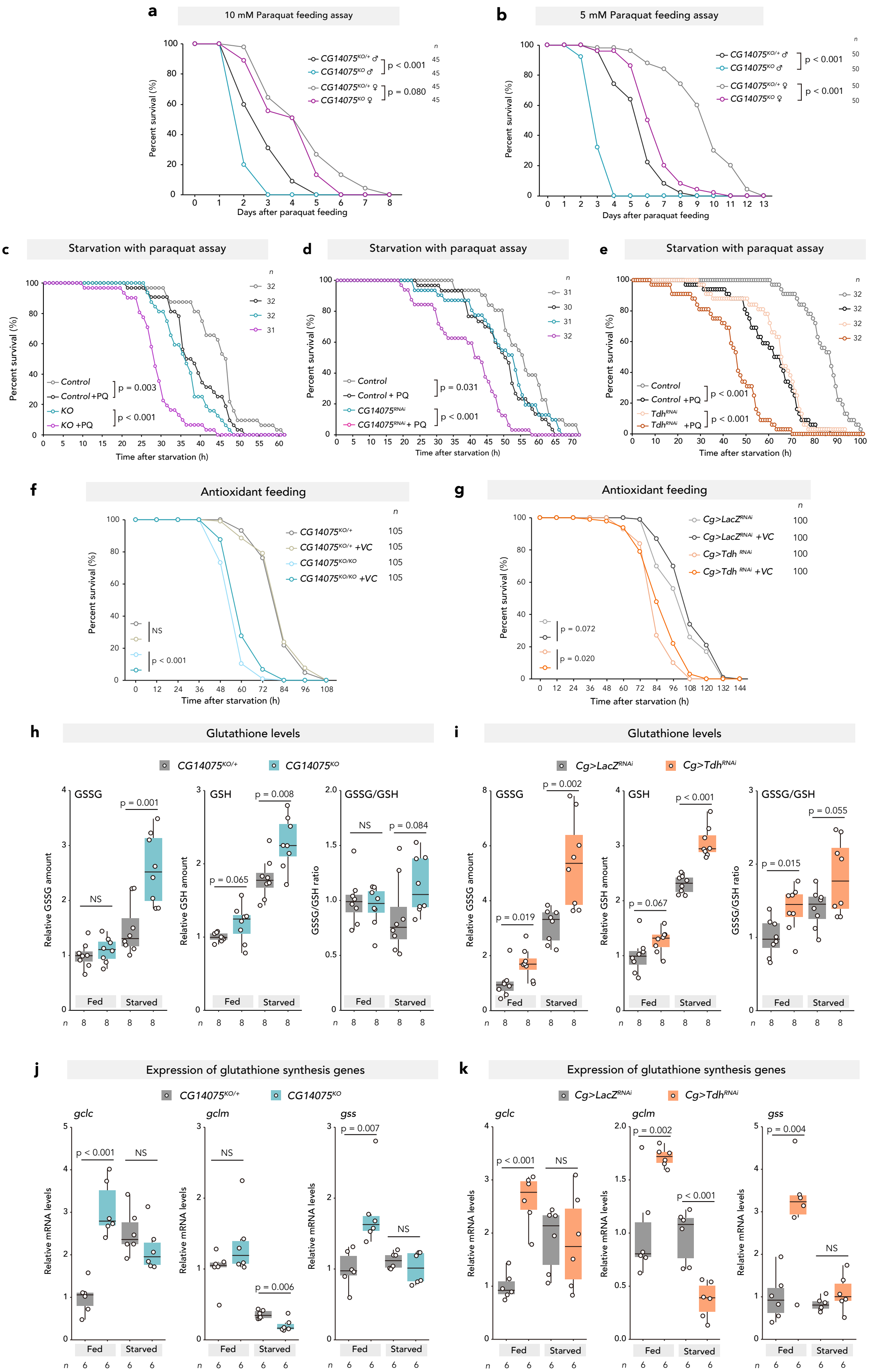

a
10 mM Paraquat feeding assay
Percent survival (%)
Days after paraquat feeding
CG14075KO/+ ♂
CG14075KO ♂
p < 0.001
CG14075KO/+ ♀
CG14075KO ♀
p = 0.080
n
45
b
5 mM Paraquat feeding assay
p < 0.001
50
c
Starvation with paraquat assay
Percent survival (%)
Time after starvation (h)
Control
Control +PQ
KO
KO +PQ
p = 0.003
p < 0.001
d
Starvation with paraquat assay
Control
Control + PQ
CG14075RNAi
CG14075RNAi + PQ
p = 0.031
p < 0.001
e
Starvation with paraquat assay
Control
Control +PQ
TdhRNAi
TdhRNAi +PQ
p < 0.001
f
Antioxidant feeding
CG14075KO/+
CG14075KO/+ +VC
CG14075KO/KO
CG14075KO/KO +VC
105
NS
p < 0.001
g
Antioxidant feeding
Cg>LacZRNAi
Cg>LacZRNAi +VC
Cg>Tdh RNAi
Cg>Tdh RNAi +VC
100
p = 0.072
p = 0.020
h
Glutathione levels
CG14075KO/+
CG14075KO
GSSG
GSH
GSSG/GSH
Relative GSSG amount
Relative GSH amount
GSSG/GSH ratio
p = 0.001
p = 0.008
p = 0.065
p = 0.084
NS
Fed
Starved
n
8
i
Glutathione levels
Cg>LacZRNAi
Cg>TdhRNAi
p = 0.002
p = 0.019
p < 0.001
p = 0.067
p = 0.055
p = 0.015
j
Expression of glutathione synthesis genes
CG14075KO/+
CG14075KO
gclc
gclm
gss
Relative mRNA levels
p < 0.001
NS
p = 0.006
p = 0.007
6
k
Expression of glutathione synthesis genes
Cg>LacZRNAi
Cg>TdhRNAi
p < 0.001
p = 0.002
p = 0.004

Supplementary Fig. 10 | Oxidative stress and antioxidant-related phenotypes in *CG14075*^{KO} and *Tdh* knockdown flies.

a, Survival curves of control and *CG14075*^{KO} flies on a 10 mM paraquat-supplemented diet. **b**, Survival curves of control and *CG14075*^{KO} flies on a 5 mM paraquat-supplemented diet. **c**, Starvation survival curves of control (*CG14075*$^{KO/+}$) and *CG14075*^{KO} female flies with or without 5 mM paraquat (PQ). **d**, Starvation survival curves of control (*Akh>LacZ*^{RNAi}) and CC-specific *CG14075* knockdown female flies (*Akh>CG14075*^{RNAi}) with or without 5 mM paraquat. **e**, Starvation survival curves of control (*Cg>LacZ*^{RNAi}) and adipose tissue-specific *Tdh* knockdown female flies (*Cg>Tdh*^{RNAi}) with or without 5 mM paraquat. **f**, Starvation survival curves of control and *CG14075*^{KO} flies fed a standard diet with or without vitamin C prior to starvation. **g**, Starvation survival curves of control and *Tdh* knockdown flies fed a standard diet with or without vitamin C prior to starvation. **h**, Glutathione levels in control and *CG14075*^{KO} flies. **i**, Glutathione levels in control and *Tdh* knockdown flies. **j, k**, mRNA expression levels of glutathione synthesis genes in the abdominal carcass of flies as in h and i.

Statistics: Log-rank test (a-e), Pairwise log-rank test (f, g), unpaired two-tailed Student’s t-test (h, i, j, k). NS, not significant ($P > 0.05$). Box plots, centre line (median), box limits (first and third quartiles), and whiskers (most extreme data points within 1.5× the interquartile range). Sample sizes (n) are indicated in each panel.

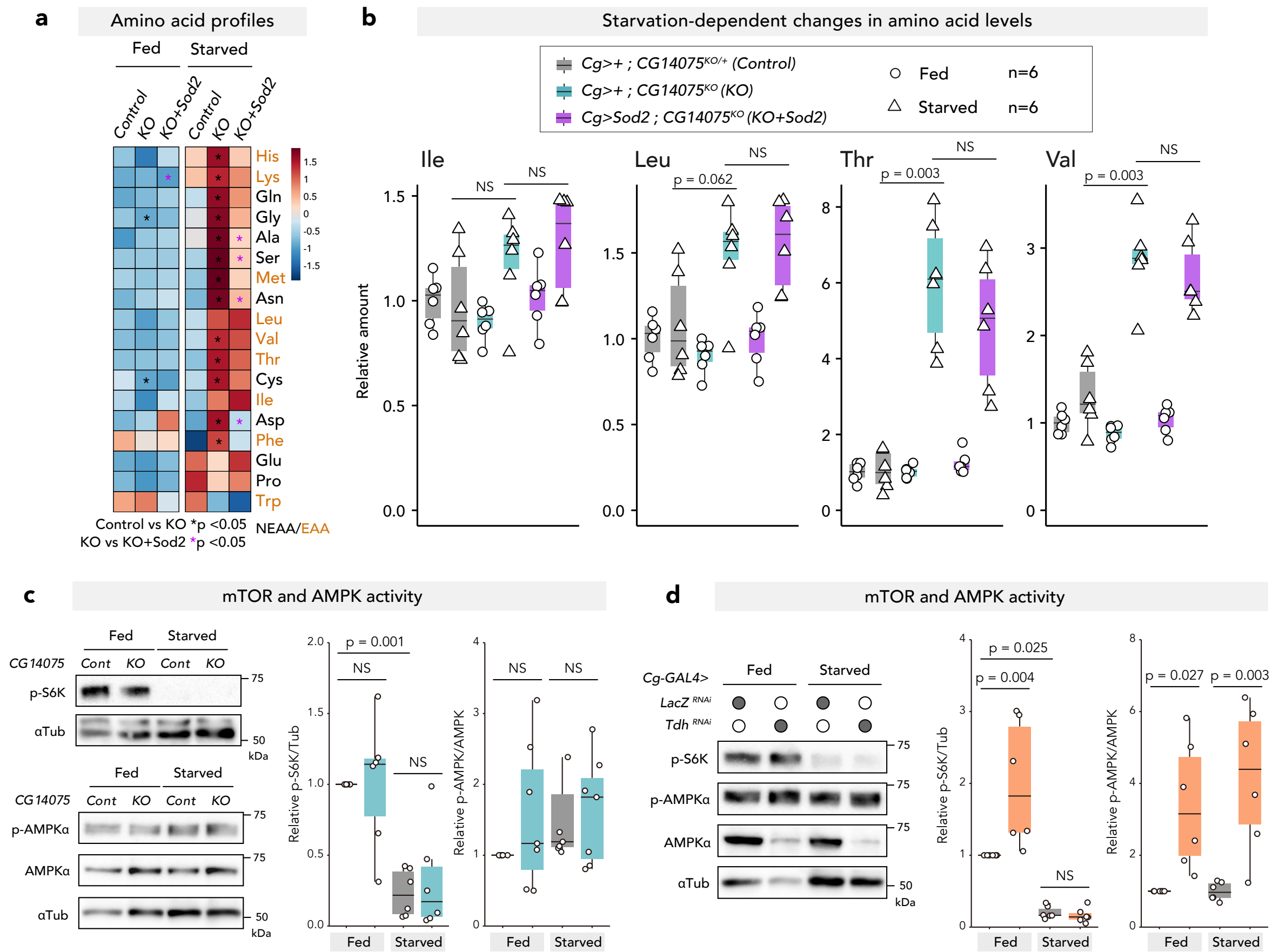

Supplementary Fig. 11 | *Sod2* overexpression does not suppress amino acid accumulation.

a, Heatmap of amino acid profiles in control, *CG14075*^{KO}, and fat body-specific *Sod2* overexpressing-female flies. EAAs are highlighted in orange. Asterisks indicate statistical significance between control, *CG14075*^{KO} and *Sod2* overexpressed flies under fed and starved conditions. **b**, Relative levels of BCAAs and threonine as in (a). **c, d**, Phosphorylation levels of AMPKα and the mTOR target protein S6K. n=6, each. Representative western blots from two independent experiments with similar results are shown.

Statistics: pairwise Wilcoxon rank-sum test (a, b), and one-way ANOVA followed by Tukey's multiple comparisons test (c, d). NS, not significant (P > 0.05). Box plots, centre line (median), box limits (first and third quartiles), and whiskers (most extreme data points within 1.5× the interquartile range). Sample sizes (n) are indicated in each panel or legends.

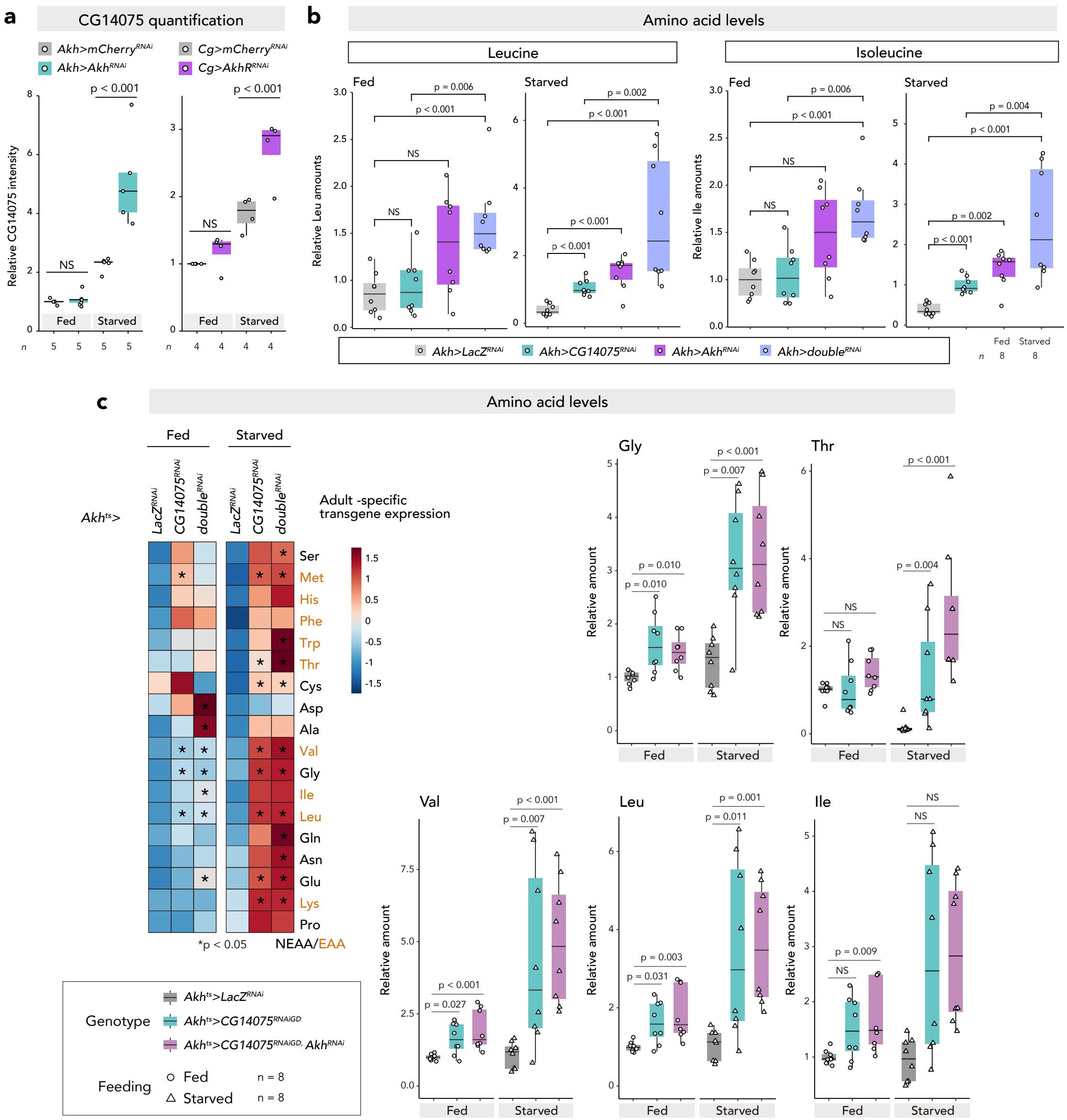

Supplementary Fig. 12 | CG14075 and AKH regulate amino acid levels.

a, Quantification of CG14075 secretion in control, CC-specific *Akh* knockdown and adipose tissue-specific *AkhR* knockdown flies, related to Fig. 6b. **b**, Relative levels of leucine and isoleucine, related to Fig. 6f. **c**, Left, heatmap of amino acid profiles in control, adult-specific *CG14075* knockdown, and simultaneous *CG14075* and *Akh* knockdown (*double*^{RNAi}) flies. EAAs are highlighted in orange. Asterisks indicate statistical significance between control and each RNAi genotype under fed and starved conditions. Right, relative levels of glycine, threonine, and BCAAs.

Statistics: One-way ANOVA followed by Tukey's multiple comparisons test (a), and pairwise Wilcoxon rank-sum test (b, c). *P < 0.05; NS, not significant (P > 0.05). Box plots, centre line (median), box limits (first and third quartiles), and whiskers (most extreme data points within 1.5× the interquartile range). Sample sizes (n) are indicated in each panel.
