# Supplementary material for: CG14075/Mmt promotes amino acid catabolism and cooperates with AKH during starvation in Drosophila
Source: Nat Commun. 2026 Sep 12;17:10766. doi: 10.1038/s41467-026-77687-7 (PMC13661902; doi:10.1038/s41467-026-77687-7)
Supplement: Supplementary file 2 [file 41467_2026_77687_MOESM2_ESM.pdf]

Description of Additional Supplementary Files

Supplementary Data 1
Description: Primer and oligonucleotide sequences used in this study.

Supplementary Data 2
Description: qPCR primer sequences used for validation of the RNA-seq results shown in Fig. 1.

Supplementary Data 3
Description: RNA-seq read counts and related expression data used to identify genes enriched in CC-containing neuronal ganglia.

Supplementary Data 4
Description: Protein/gene names, species, accession numbers or database IDs, and amino acid sequences used for the multiple sequence alignment of CG14075/Mmt-related proteins shown in Supplementary Fig. 8a.
