# Supplementary material for: CG14075/Mmt promotes amino acid catabolism and cooperates with AKH during starvation in Drosophila
Source: Nat Commun. 2026 Sep 12;17:10766. doi: 10.1038/s41467-026-77687-7 (PMC13661902; doi:10.1038/s41467-026-77687-7)
Supplement: Supplementary file 3 [file 41467_2026_77687_MOESM4_ESM.pdf]

nature portfolio

Corresponding author(s): Yuto Yoshinari, Ryusuke Niwa, and Takashi Nishimura

Last updated by author(s): Aug 17, 2026

Reporting Summary

Nature Portfolio wishes to improve the reproducibility of the work that we publish. This form provides structure for consistency and transparency in reporting. For further information on Nature Portfolio policies, see our Editorial Policies and the Editorial Policy Checklist.

Statistics

For all statistical analyses, confirm that the following items are present in the figure legend, table legend, main text, or Methods section.

n/a	Confirmed	
☐	☒	The exact sample size (*n*) for each experimental group/condition, given as a discrete number and unit of measurement
☐	☒	A statement on whether measurements were taken from distinct samples or whether the same sample was measured repeatedly
☐	☒	The statistical test(s) used AND whether they are one- or two-sided<br>*Only common tests should be described solely by name; describe more complex techniques in the Methods section.*
☒	☐	A description of all covariates tested
☐	☒	A description of any assumptions or corrections, such as tests of normality and adjustment for multiple comparisons
☐	☒	A full description of the statistical parameters including central tendency (e.g. means) or other basic estimates (e.g. regression coefficient) AND variation (e.g. standard deviation) or associated estimates of uncertainty (e.g. confidence intervals)
☐	☒	For null hypothesis testing, the test statistic (e.g. *F*, *t*, *r*) with confidence intervals, effect sizes, degrees of freedom and *P* value noted<br>*Give P values as exact values whenever suitable.*
☒	☐	For Bayesian analysis, information on the choice of priors and Markov chain Monte Carlo settings
☒	☐	For hierarchical and complex designs, identification of the appropriate level for tests and full reporting of outcomes
☒	☐	Estimates of effect sizes (e.g. Cohen's *d*, Pearson's *r*), indicating how they were calculated

Our web collection on statistics for biologists contains articles on many of the points above.

Software and code

Policy information about availability of computer code

Data collection	ZEN 2.3 (Zeiss) was used for confocal image acquisition.MassHunter GC/MS Acquisition B.07.04.2260 (Agilent Technologies) was used for GC–MS data acquisition. MassLynx 4.1 (Waters) was used for LC-MS data acquisition.
Data analysis	MassHunter Quantitative Analysis version B.07.01 (Agilent Technologies) was used for GC–MS peak area analysis.MassLynx 4.1 (Waters) were used for LC-MS data analysis. Fiji 2.16.0/ImageJ was used for image analysis. Microsoft Excel version 16.101, R version 4.4.2 and RStudio version 2024.12.0 were used for data processing, statistical analyses and graphing.

For manuscripts utilizing custom algorithms or software that are central to the research but not yet described in published literature, software must be made available to editors and reviewers. We strongly encourage code deposition in a community repository (e.g. GitHub). See the Nature Portfolio guidelines for submitting code & software for further information.

Data

Policy information about availability of data

All manuscripts must include a data availability statement. This statement should provide the following information, where applicable:

- Accession codes, unique identifiers, or web links for publicly available datasets
- A description of any restrictions on data availability
- For clinical datasets or third party data, please ensure that the statement adheres to our policy

Source data are provided with this paper. The RNA-seq data generated in this study have been deposited in the DNA Data Bank of Japan Sequence Read Archive

under BioProject accession code PRJDB35995 [https://ddbj.nig.ac.jp/search/entry/bioproject/PRJDB35995]. The RNA-seq read counts and related data are provided in Supplementary Data 3. The Drosophila melanogaster BDGP6 reference genome used for RNA-seq read mapping is available under accession code GCA_000001215.4 [https://www.ncbi.nlm.nih.gov/datasets/genome/GCA_000001215.4/]. The previously published Fly Cell Atlas datasets used for single-cell expression visualization are available under accession codes E-MTAB-10519 [https://www.ebi.ac.uk/gxa/sc/experiments/E-MTAB-10519] and E-MTAB-10628 [https://www.ebi.ac.uk/gxa/sc/experiments/E-MTAB-10628]. Primer information is provided in Supplementary Data 1 and 2. No custom code was generated for this study. Protein/gene names, species, accession numbers or database IDs, and amino acid sequences used for the CG14075/Mmt-related protein alignment are provided in Supplementary Data 4. No custom code was generated for this study.

Research involving human participants, their data, or biological material

Policy information about studies with human participants or human data. See also policy information about sex, gender (identity/presentation), and sexual orientation and race, ethnicity and racism.

Reporting on sex and gender	N/A
Reporting on race, ethnicity, or other socially relevant groupings	N/A
Population characteristics	N/A
Recruitment	N/A
Ethics oversight	N/A

Note that full information on the approval of the study protocol must also be provided in the manuscript.

Field-specific reporting

Please select the one below that is the best fit for your research. If you are not sure, read the appropriate sections before making your selection.

☒ Life sciences ☐ Behavioural & social sciences ☐ Ecological, evolutionary & environmental sciences

For a reference copy of the document with all sections, see nature.com/documents/nr-reporting-summary-flat.pdf

Life sciences study design

All studies must disclose on these points even when the disclosure is negative.

Sample size	No statistical methods were used to predetermine sample size. Sample sizes were chosen based on prior experience and common practice in the field and were sufficient to detect reproducible effects.
Data exclusions	No data were excluded from the analysis.
Replication	Experiments were independently repeated at least twice using separately reared populations, with similar results. Unless otherwise stated, data shown are pooled from independent biological replicates.
Randomization	Within each genotype, staged animals were randomly assigned to dietary conditions. Sample processing and measurements were performed in a randomized order when feasible.
Blinding	Blinding was not performed. The investigator conducting the experiments also performed sample collection and analysis. Samples were collected and processed in parallel under identical conditions with appropriate controls.

Reporting for specific materials, systems and methods

We require information from authors about some types of materials, experimental systems and methods used in many studies. Here, indicate whether each material, system or method listed is relevant to your study. If you are not sure if a list item applies to your research, read the appropriate section before selecting a response.

Materials & experimental systems

n/a	Involved in the study	
☐	☒	Antibodies
☐	☒	Eukaryotic cell lines
☒	☐	Palaeontology and archaeology
☐	☒	Animals and other organisms
☒	☐	Clinical data
☒	☐	Dual use research of concern
☒	☐	Plants

Methods

n/a	Involved in the study	
☒	☐	ChIP-seq
☒	☐	Flow cytometry
☒	☐	MRI-based neuroimaging

Antibodies

Antibodies used

mouse anti-Akh antibody (Yoshinari et al., 2024)
guniea pig anti-CG14075 antibody (This study)
chicken anti-GFP antibody (Abcam, ab13970)
rabbit anti-phospho-S6K (Cell Signaling Technology, #9209)
rabbit anti-phospho-AMPKα (Cell Signaling Technology, #2535)
rabbit anti-total AMPKα (Cell Signaling Technology, #2532)
rat anti-αTubulin (Thermo Fisher, #MA1-80017)
HRP-linked anti-guinea pig IgG (Millipore, #AP193P)
HRP-linked anti-rabbit IgG (Cell Signaling Technology, #7074)
HRP-linked anti-rat IgG (Cell Signaling Technology, #7077)
Alexa Fluor 488-conjugated goat anti-chicken IgY (A-11039, Thermo Fisher Scientific)
Alexa Fluor 555-conjugated goat anti-mouse IgG (A-21424, Thermo Fisher Scientific)
Alexa Fluor 488–conjugated goat anti-guinea pig IgG (A-11073, Thermo Fisher Scientific)
Alexa Fluor 555–conjugated goat anti-guinea pig IgG (A-21435, Thermo Fisher Scientific)

Validation

For all commercially sourced primary antibodies, specificity and application information were based on the manufacturers' datasheets/websites prior publications. The mouse anti-AKH antibody was used as previously described(PMID: 39737959). The custom guinea pig anti-CG14075/Mmt antibody was generated against a KLH-conjugated CG14075/Mmt peptide and validated in this study by loss of signal in CG14075/Mmt mutant or knockdown samples in immunostaining and western blot analyses.

Eukaryotic cell lines

Policy information about cell lines and Sex and Gender in Research

Cell line source(s)

HEK293T cells were obtained from the American Type Culture Collection.

Authentication

The HEK293T cells used in this study were not authenticated after receipt.

Mycoplasma contamination

HEK293T cells were treated with 0.5 μg/ml MC-210 (DS Pharma Biomedical) as a precaution before cryopreservation. Cells were used from these frozen stocks and were not tested for mycoplasma contamination after thawing.

Commonly misidentified lines
(See ICLAC register)

No commonly misidentified cell lines were used in this study.

Animals and other research organisms

Policy information about studies involving animals; ARRIVE guidelines recommended for reporting animal research, and Sex and Gender in Research

Laboratory animals

The following Drosophila melanogaster strains were used in this study. UAS-LacZRNAi was a gift from Masayuki Miura, the University of Tokyo, and UAS-grim was a gift from Dr. Michael B. O'Connor.

The following Drosophila melanogaster strains were obtained from the Bloomington Drosophila Stock Center (BDSC): Akh-Gal4 (#25684), Akh-Gal4 (#25683), UAS-mCherryRNAi (#35785), UAS-Luciferase (#35788), UAS-mCD8-GFP (#32186), Tub-Gal4 (#5138), Tub-Gal80ts (#7017), Cg-Gal4 (#7011), UAS-bmmRNAi (#25926), UAS-BcatRNAi (#38363), UAS-stinger (#84277) and UAS-kir2.1 (#6596).

The following Drosophila melanogaster strains were obtained from the Vienna Drosophila Resource Center (VDRC): VT013947-Gal4 (#205650), UAS-CG14075RNAiGD (#14238), UAS-CG14075RNAiKK (#102202), UAS-AkhRNAi (#105063) and UAS-LstRNAi (#106861).

The following Drosophila melanogaster strains were obtained from the NIG-FLY Stock Center: UAS-Tps1RNAi (#4104R-6), UAS-TrehRNAi (#9364R-1), UAS-GcatRNAi (#HMC02902) and UAS-TdhRNAi (#5955R-3).

The following Drosophila melanogaster strains were generated in this study: UAS-CG14075::HA, UAS-CG14075-N, UAS-CG14075-C

and CG14075KO.

Wild animals	No wild animals were used in the study.
Reporting on sex	Unless otherwise indicated, experiments were performed using adult virgin females. When adult male or mated females were used, they were collected from mixed-sex vials, and this is specified in the corresponding figure legends.
Field-collected samples	No field collected samples were used in the study.
Ethics oversight	This study did not require an ethical approval.

Note that full information on the approval of the study protocol must also be provided in the manuscript.

Plants

Seed stocks	N/A
Novel plant genotypes	N/A
Authentication	N/A
