# Supplementary material for: CG14075/Mmt promotes amino acid catabolism and cooperates with AKH during starvation in Drosophila
Source: Nat Commun. 2026 Sep 12;17:10766. doi: 10.1038/s41467-026-77687-7 (PMC13661902; doi:10.1038/s41467-026-77687-7)
Supplement: Supplementary file 4 [file 41467_2026_77687_MOESM5_ESM.pdf]

nature portfolio

Peer Review File

CG14075/Mmt promotes amino acid catabolism and cooperates with AKH during starvation in Drosophila

Corresponding Author: Dr Yuto Yoshinari

This file contains all reviewer reports in order by version, followed by all author rebuttals in order by version.

Version 0:

Reviewer comments:

Reviewer #1

(Remarks to the Author)
The manuscript explores an interesting and potentially important role for CG14075/Mmt in starvation physiology and feeding regulation. The study combines genetic, metabolic, and imaging approaches to dissect the function of this peptide, which the authors propose acts as a novel CC-derived hormone that modulates amino acid utilization and feeding behavior under nutrient stress. While the topic is timely and of broad interest, several of the central claims are not yet fully supported by the presented data. In particular, the evidence that CG14075 is produced and secreted from the CC, functions independently of AKH, and directly regulates metabolism requires stronger experimental support. Adult-specific CC manipulations with independent RNAi lines and appropriate UAS/background controls are needed to exclude developmental effects and genetic-background artifacts. Quantitative, absolute metabolite measurements and controlled feeding/secretion assays are also essential to resolve current inconsistencies and to substantiate the central claims.

Major points
1. No genetic rescue.
There is no demonstration that CC-specific re-expression of CG14075/Mmt in the knockout background restores survival or metabolic phenotypes. The authors possess UAS-CG14075, but this is used for overexpression (Tub-Gal4ts) rather than add-back in the KO background for CC-specific rescue. Demonstrating rescue is a major standard for establishing causality and would strongly validate the model.

2. CC ablation versus AKH knockdown.
Initially, the authors compare Akh knockdown and CC ablation, using the stronger CC-ablation phenotype to argue that the CC contributes to starvation resistance through mechanisms beyond AKH. A more parsimonious explanation is that the knockdown does not produce a complete loss of function.

3. CG14075 expression pattern and CC specificity.
It is unclear why CG14075 is not enriched in the CC in Fig. 1e, and the manuscript does not present clear data supporting is predominantly expressed in the CC. If the RNA-seq data are correct, this suggests that CG14075 is not CC-specific. Public expression datasets also indicate strong neuronal expression of CG14075.

4. CG14075 localization (Fig. 1f).
Overlap between CG14075 and AKH signals appears limited. Although CG14075 staining disappears in the null mutant, confirming the CC as the source requires repeating the experiment with CC-specific CG14075 knockdown, staining for both AKH and CG14075 to verify loss specifically in CC cells.

5. Inconsistent phenotypes and interpretation (Fig. 2a,b).
Global or CC loss of CG14075 increases starvation sensitivity, yet CC ablation increased starvation resistance and was previously used to argue for other CC-derived factors. If CC ablation removes both AKH and CG14075, the phenotype should be weaker than AKH knockdown alone. The current logic and interpretation need clarification and CG14075 cannot be that factor(s) beyond Akh.

6. Metabolite measurements and normalization (Fig. 2c).
To claim that the CC is the source of metabolism-regulating CG14075, experiments in Fig. 2c should be repeated with CC-

specific, adult-restricted knockdown, using independent RNAi lines and UAS controls. The current metabolite data show large variance. TAG levels appear lower at baseline in animals lacking CG14075 but decline at a similar rate during starvation. Glycogen is higher at baseline and is not depleted before TAG during starvation, which is expected. If CG14075 affects protein, normalizing metabolites to protein may be inappropriate. The authors should report absolute metabolite values and analyze depletion trajectories using two-way ANOVA (time x genotype) rather than separate t-tests. Because CG14075 likely has developmental roles, adult-specific CC knockdown is necessary to exclude developmental effects.

7. Lipid droplet and feeding assays (Fig. 2d–g).
Lipid droplets in Fig. 2d should be quantified. For Fig. 2e–g, CAFE assays with either sugar or yeast should be added to corroborate FlyPAD data, and a dye-based intake assay with sugar or yeast would directly measure consumption. Discrepancies between FlyPAD (effect) and CAFE (no effect) must be resolved with better-controlled feeding experiments. As some "neuronal" drivers may also target the CC, CC-specific manipulations, a second RNAi line, and adult-specific designs are required.

8. CG14075 regulation by nutrients (Fig. 3g,i).
Refeeding with peptone is of limited value because flies consume little of it (they do not like it). Testing yeast refeeding would clarify whether sugar specifically regulates CG14075 secretion. These experiments should also be repeated in CC-specific CG14075 knockdown flies to confirm the CC as the source. In Fig. 3i, showing loss of the CG14075 signal with CC-specific knockdown would strengthen the conclusion, especially since the staining pattern is atypical for a secreted peptide and lacks clear granularity. The finding that secretion occurs only after 36–48 h of starvation raises concerns about physiological relevance, as flies are close to death at this time.

9. Amino-acid metabolism and overexpression (Fig. 4).
CG14075 loss impairs amino-acid consumption during starvation, but this must be tested with adult-specific CC knockdown and in AKH-deficient backgrounds to determine whether effects are dependent or independent of AKH. Using Tub> for overexpression does not isolate CC-derived effects AKH> would be more appropriate. The reported 24-hour requirement for CG14075-induced amino-acid breakdown, combined with its late secretion (36-48 hours), challenges physiological plausibility. If CG14075 acts directly, shorter-term effects should be detectable. To establish mechanistic linkage to amino-acid catabolism, overexpress CG14075 while knocking down Tdh in the fat body to test whether the effect is abolished. This requires perhaps the LexA system.

10. Relationship to AKH signaling, metabolic inconsistencies and quantifications (Figs. 5-6).
Provide quantification for Figs. 5f,g and 6b (and why does the non-specific band increase in 6b?). CG14075 loss increases AKH secretion during starvation, suggesting that CG14075 effects might be mediated via AKH. This should be tested explicitly in AKH mutants or double knockdowns. It is puzzling that the double knockdown yields greater starvation resistance than AKH knockdown alone. If CG14075 opposes AKH, an intermediate phenotype would be expected. Moreover, some metabolic results contradict established literature: AKH-loss flies typically show higher TAG and higher glycogen, whereas here they show lower glycogen and no TAG elevation. These inconsistencies suggest potential measurement or background issues. Proper UAS controls (reconsidering whether UAS-LacZ-RNAi is the appropriate background) and background verification are essential. When basic metabolic data diverge from consensus, confidence in amino-acid measurements (which lack external benchmarks) is weakened.

11. Statistics and distributional assumptions (e.g., Fig. 6e).
Datasets such as activity in Fig. 6e appear non-normally distributed, yet were analyzed with Dunnett's test, which assumes normality. As also mentioned above two-way ANOVA should be used to analyze interactions rather than multiple t-tests per time point.

In summary, the manuscript provides a valuable dataset and a potentially important new angle on CC signaling and starvation physiology. However, the current evidence does not yet convincingly demonstrate that CG14075 is a new CC hormone acting independently of AKH to regulate metabolism and feeding. Addressing the key issues outlined above would substantially strengthen the conclusions and elevate the work to the level required for publication in a high-impact journal.

Reviewer #2

(Remarks to the Author)
The study identifies CG14075/Mmt as a novel peptide secreted from the Drosophila corpora cardiaca (CC) that promotes starvation resistance. While Akh knockdown improved survival, complete CC ablation conferred even greater protection, suggesting additional factors. RNA-seq and RT-qPCR validation pinpointed CG14075/Mmt as a CC specific peptide, whose loss led to starvation sensitivity, excessive depletion of lipid and carbohydrate stores, and elevated feeding. CG14075/Mmt is secreted in the hemolymph in response to a prolonged starvation and suppressed by sugar refeeding. The authors propose that CG14075/Mmt and Akh operate in a reciprocal feedback loop, balancing energy metabolism during starvation. Mechanistically, CG14075/Mmt promotes threonine and branched-chain amino acid catabolism by inducing Bcat and Gcat expression. Its cysteine-rich C-terminal domain is essential for function, and ex-vivo peptide treatment stimulates peripheral tissue metabolism. Loss of CG14075/Mmt or fat body threonine dehydrogenase (Tdh) impaired redox homeostasis and enhanced oxidative stress. Thus, CG14075/Mmt acts as a CC-derived hormone-like factor linking amino acid metabolism to starvation resilience. Together, these findings identify CG14075/Mmt as a novel CC-derived, hormone-like peptide that coordinates the metabolic transition from lipid/carbohydrate to amino acid utilization during prolonged starvation. The study proposes that CG14075/Mmt driven threonine catabolism in the fat body maintains redox balance and energy homeostasis, thereby promoting starvation resistance.

While the authors present compelling evidence supporting the role of CG14075/Mmt as a hormone-like peptide that mediates the starvation response through amino acid catabolism, several aspects require clarification and strengthening. The study lacks a defined molecular mechanism and does not identify a receptor or signaling pathway for CG14075/Mmt. Additionally, there is an inconsistency between the corpora cardiaca (CC) ablation phenotype and the CG14075/Mmt loss-of-function phenotype, which needs to be reconciled. Most major claims including interactions with Akh, regulation of amino acid metabolism, and redox control are based primarily on indirect or correlative evidence rather than direct functional assays.

A major shortcoming is the absence of a known receptor and undefined target tissues, leaving the hormone-like classification mechanistically incomplete. Because the authors have not identified a receptor, which we realize is non-trivial, the data presented in the manuscript need to be carefully presented/discussed considering indirect mechanisms.

Overall, this is an exciting report with a number of very interesting findings. However, the study has a number of shortcomings that requires major revisions. The following points merit further investigation:

1. “CG14075/Mmt is a novel secreted hormone-like peptide involved in starvation resistance”

- While CG14075/Mmt is detectable in the hemolymph, direct evidence for active secretion from the CC is limited. This could be addressed by ex vivo CC culture and measurement of Mmt release into the media, or by blocking CC-specific secretion and showing a corresponding loss of Mmt from the hemolymph. Immunostaining of secretory vesicles and their colocalization with Mmt could further support regulated secretion. Although not strictly required, CC-specific reintroduction of Mmt to rescue starvation sensitivity would strengthen the causal link. Also, have the authors considered that CG14075/Mmt is a distinct isoform from the one from the brain and plays distinct role in starvation resistance?

2. “CG14075/Mmt indirectly regulates lipid and carbohydrate depletion via Akh”

- While there is a clear reduction in lipid and carbohydrate levels in Mmt mutants during starvation, the tissue specificity of Mmt remains unclear. The authors have shown a reduction in TAG in the fat body in MmtKO animals, however, experiments for fat body specific Mmt knockdown or rescue and changes in energy metabolism would be useful. The observation that Mmt mutants feed more but still lose energy reserves suggests other systemic defects not explained by Akh modulation alone. Authors have mentioned a feedback loop between Akh and Mmt and provided data for changes in systemic metabolites driving this loop. However, the feedback claim lacks functional tests. For example, whether Mmt acts directly on CC cells to alter Akh secretion or synthesis is untested.

3. “CG14075/Mmt regulates amino acid metabolism”

- Changes in amino acid metabolism in CG14075/Mmt mutant flies could potentially result from a secondary transcriptional response to altered energy or redox state. A rescue experiment (CC specific reintroduction of Mmt in Mmt mutant background) would be appropriate. Low level of Mmt could be detected from the onset of starvation, so it would be appropriate to test if there is a low level of amino acid catabolism by Mmt at this stage. This might explain the lower level of TAG and glycogen in MmtKO animals from the very early stage of starvation.

4. “CG14075/Mmt-mediated threonine metabolism is critical for starvation resistance and oxidative stress tolerance”

- A direct relationship between CC derived CG14075/Mmt and threonine metabolism in the fat body is not well established. How does Tdh expression look like in Akh>CG14075RNAi animals? The causal link between threonine catabolism and redox balance is speculative. Only marker genes (GstD1, Sod2) were used, no direct ROS measurements or redox assays (e.g., ROS imaging, $NADPH/NADP^+$ ratio). The hypothesis that threonine catabolism maintains oxidative homeostasis would be strengthened by metabolite profiling ($NADH/NAD^+$, GSH/GSSG) or rescue with antioxidant treatments. The authors should also clarify how threonine catabolism contributes to starvation resistance. Is this effect primarily due to increased energy substrate availability (e.g., acetyl-CoA or TCA intermediates), modulation of oxidative stress, altered lipid metabolism, or cross-talk with signaling pathways such as mTOR or AMPK? In lines 304–305: “Tdh knockdown in the fat body led to a reduction of TAG levels under both fed and starved conditions.” This observation resembles the phenotype seen in Mmt mutants, which show reduced TAG and glycogen even in the fed state. To mechanistically link these findings, the authors could perform isotope tracing (e.g., using ^{15}N-$^{13}C_4$-threonine) in control, MmtKO, and cg>Tdh-RNAi flies to quantify the proportion of TAG derived from threonine catabolism under fed conditions. This would help clarify whether Mmt also contributes to energy storage during the fed state, in addition to its proposed role in suppressing Akh secretion during starvation.

5. Need to clarify the CC ablation procedure. Exact time of UAS-grim induction and how long did it take to achieve ablation. Also, did you try any other procedure for ablation. Was it a complete or partial ablation? Please include ablation data in the supplement section.

6. Have the authors tried inducing starvation like condition by genetic manipulations of either insulin and TOR signaling pathway and measuring hemolymph CG14075/Mmt levels? This would help further establish the point that the lack of systemic energy level induces the secretion of CG14075/Mmt.

7. In order to establish the point made at line 208-209 “CG14075/Mmt secretion is sensitive to carbohydrate availability, not directly to TAG levels.”, have you tried feeding starved animals a high fat diet and measuring changes in of CG14075/Mmt level?

8. Although the authors have provided evidence for lipid loss in CG14075/Mmt mutant animals both in fed and starved condition, more experiments are needed to substantiate the claim that CG14075/Mmt suppresses lipolysis (line 336).

9. Need better characterization of the Akh and Mmt role in amino acid catabolism to substantiate the claim in line 360-361 “CG14075/Mmt and AKH act in concert to regulate starvation dependent amino acid metabolism”

Minor comments:

- Is CG14075/Mmt expressed during the larval stage as well? Do MmtKO mutants develop into adults with normal morphology and viability comparable to wild-type animals?
- Figure 1g, add an arrow to point the band in the WB that corresponds to CG14075/Mmt

- Figure 3i- need to add better quality image. Punctate structures are not clearly visible, and they do not match their localization profile described in the text.
- Extended data fig 3h, i is missing- please add them during next submission
- Figure 6b, need quantification of CG14075 in Akh-RNAi and AkhR-RNAi animals.
- Figure 6g, need to adjust the model figure as there is not enough evidence for CG14075 to suppress lipolysis and glycogenolysis.
- Line 231-232, it would be appropriate to mention that there is a lower level of CG14075 presence during very early stages of starvation and it goes up with energy storage depletion.
- Line 234, need citation here- "During prolonged starvation, organisms increasingly mobilize amino acids to support gluconeogenesis and ketogenesis"
- Figure 4a, a vertical line on the x-axis at value 1 to show the changes in amino acid levels more clearly.

Version 1:

Reviewer comments:

Reviewer #1

(Remarks to the Author)
The authors have adequately addressed my concerns, and the revised manuscript is significantly improved and suitable for publication.

Reviewer #2

(Remarks to the Author)
The authors have revised the manuscript and performed additional experiments that strengthen the evidence supporting CG14075/Mmt as a CC-derived secreted factor involved in starvation resistance and systemic metabolic regulation. In particular, the CC-specific rescue experiments and functional inhibition of CC activity provide stronger support for a CC origin of Mmt. However, several important conceptual and mechanistic issues remain unresolved, including the lack of identification of the Mmt receptor and target tissue, and the incomplete understanding of how Mmt regulates amino acid metabolism and interacts with AKH signaling, and how systemic energy depletion regulates Mmt secretion.

1. Secretion of CG14075/Mmt from CC
Assessment: Partially addressed
The authors provide improved evidence for CC-dependent secretion by functionally inhibiting CC activity (Kir2.1), which reduces starvation-induced Mmt levels in the hemolymph. In addition, CC-specific rescue of Mmt in mutant animals restores starvation resistance and metabolic phenotypes, supporting a CC-derived systemic role.
However, the subcellular localization of Mmt remains unclear (Fig. 3i). While line plot analyses suggest punctate structures, the imaging data lack sufficient resolution to convincingly demonstrate distinct localization or secretion dynamics. Higher-resolution images and co-localization with established secretory vesicle markers would substantially strengthen this claim.
Additionally, the possibility of distinct Mmt isoforms (e.g., CC vs brain-derived) should be acknowledged and discussed.
2. Role of Mmt in systemic energy metabolism and interaction with AKH
Assessment: Partially addressed
The absence of phenotypic effects upon fat body–specific knockdown supports the conclusion that Mmt is unlikely to originate from the fat body and is instead secreted from the CC.
However, the downstream target tissue and mechanism of action remain unclear. Additional experiments—such as tissue-specific rescue (e.g., fat body reintroduction of Mmt in mutant background)—would strengthen the model of systemic action.
The proposed feedback interaction between Mmt and AKH remains insufficiently defined. The authors have conducted ex vivo live calcium imaging experiments indicating that Mmt does not directly regulate its own secretion from the CC.
Functional evidence demonstrating whether Mmt directly regulates AKH secretion or synthesis is still lacking
3. Regulation of amino acid metabolism by Mmt
Assessment: Partially addressed
CC-specific rescue experiments strengthen the conclusion that Mmt contributes to starvation-associated metabolic regulation. The authors also show that Mmt is present at basal levels and may function under fed conditions.
However, the tissue specificity of Mmt function remains unclear, and it is still difficult to distinguish whether the observed metabolic changes are direct effects of Mmt or secondary systemic responses.
4. Link between Mmt and threonine metabolism / oxidative stress
Assessment: Not fully addressed
A direct mechanistic link between CC-derived Mmt and fat body–specific threonine catabolism remains lacking. While the authors show altered amino acid metabolism in Mmt mutants and Tdh knockdown animals, these datasets are not directly connected mechanistically.

The rescue of starvation sensitivity and TAG levels by antioxidant interventions (e.g., Sod2 overexpression, vitamin C) suggests that oxidative stress contributes to the phenotype. However, fat body–specific rescue experiments—such as reintroduction of Mmt or Tdh—to directly test their role in mitigating oxidative stress are still lacking.

Furthermore, experiments directly testing how threonine catabolism contributes to starvation resistance (e.g., metabolic flux or energy production) are still missing, although the authors appropriately acknowledge this limitation.

5. CC ablation procedure
Assessment: Addressed
The authors have clarified the experimental details.

6. Induction of starvation-like states
Assessment: Partially addressed
The authors attempted to induce a starvation-like condition using fat body–specific DN-mTOR expression; however, this did not trigger Mmt secretion. It remains unclear whether this manipulation effectively induces a systemic starvation state. Alternative approaches should be considered to more robustly test this hypothesis.

7. Carbohydrate vs TAG regulation of Mmt secretion
Assessment: Addressed
This point has been addressed (Fig. 3g).

8. Role in lipolysis
Assessment: Addressed
The authors have appropriately toned down their claims and now describe Mmt as contributing to lipid homeostasis rather than directly suppressing lipolysis. However, the lack of a clear mechanistic link between Mmt, AKH signaling, and amino acid metabolism should be explicitly acknowledged.

9. Interaction between Mmt and AKH in amino acid metabolism
Assessment: Partially addressed
Additional data suggest an additive effect of Mmt and AKH on amino acid catabolism during starvation. However, the interaction between these pathways remains incompletely characterized.
The authors may consider moderating their conclusions to reflect this limitation. For example, replacing: "...restraining excessive AKH-dependent responses and promoting amino acid catabolism"
with: "...promoting amino acid catabolism"

Additional comments:

Lines 494–495: The authors state that "Our data indicate that CG14075/Mmt primarily promotes the degradation of threonine and BCAAs." However, based on Figures 4a and 6f, while threonine catabolism appears to be most strongly affected in Mmt mutants, the degradation of several other amino acids is also significantly altered. Therefore, it would be more accurate to conclude that Mmt broadly influences amino acid catabolism, with threonine metabolism being the most prominently affected.
Line 532: The authors claim that "We further found that CG14075/Mmt and AKH negatively regulate each other's secretion." However, no conclusive evidence is presented to support this statement, and this claim requires further experimental validation or clarification.
Lines 534–535: The authors state that "this AKH-dependent suppression of CG14075/Mmt secretion appears to be mediated via fat body AkhR." Again, there is insufficient evidence provided to substantiate this conclusion, and this point should be addressed more rigorously.
Lines 548–550: The authors claim that "CG14075/Mmt is localized near the plasma membrane in CC cells, whereas AKH is more uniformly distributed throughout the CC cytoplasm." This distinction is not clear from the images shown in Figure 3i, and higher-resolution or more definitive localization data are needed to support this statement.

Minor comments:

- Is CG14075/Mmt expressed during the larval stage as well? Do MmtKO mutants develop into adults with normal morphology and viability comparable to wild-type animals?

The authors have mentioned that the loss of CG14075/Mmt does not cause major developmental defects under standard laboratory conditions.

- Figure 1g, add an arrow to point the band in the WB that corresponds to CG14075/Mmt

An arrow now marks the band corresponding to the expected molecular weight of Mmt based on its size.

- Figure 3i- need to add better quality image. Punctate structures are not clearly visible, and they do not match their localization profile described in the text.

Line intensity plot is useful here. However, the images provided are still vague and does not clearly show punctate structures for Mmt and Akh; need to be replaced with clearer and more zoomed in image.

- Extended data fig 3h, i is missing- please add them during next submission

Missing image panels added

- Figure 6b, need quantification of CG14075 in Akh-RNAi and AkhR-RNAi animals.

Quantification of CG14075/Mmt levels in Akh-RNAi and AkhR-RNAi animals added in extended data fig 11a

- Figure 6g, need to adjust the model figure as there is not enough evidence for CG14075 to suppress lipolysis and glycogenolysis.

Adjustment has been made to the model figure

- Line 231-232, it would be appropriate to mention that there is a lower level of CG14075 presence during very early stages of starvation and it goes up with energy storage depletion.

Revised accordingly

- Line 234, need citation here- “During prolonged starvation, organisms increasingly mobilize amino acids to support gluconeogenesis and ketogenesis”

Citation added

- Figure 4a, a vertical line on the x-axis at value 1 to show the changes in amino acid levels more clearly.

New line plots added which clearly shows changes in amino acid levels

Version 2:

Reviewer comments:

Reviewer #2

(Remarks to the Author)
The authors have addressed my concerns. The revised manuscript is much improved and appropriate for publication as far as I am concerned.

Open Access This Peer Review File is licensed under a Creative Commons Attribution 4.0 International License, which permits use, sharing, adaptation, distribution and reproduction in any medium or format, as long as you give appropriate credit to the original author(s) and the source, provide a link to the Creative Commons license, and indicate if changes were made.
In cases where reviewers are anonymous, credit should be given to 'Anonymous Referee' and the source.
The images or other third party material in this Peer Review File are included in the article’s Creative Commons license, unless indicated otherwise in a credit line to the material. If material is not included in the article’s Creative Commons license and your intended use is not permitted by statutory regulation or exceeds the permitted use, you will need to obtain

permission directly from the copyright holder.
To view a copy of this license, visit https://creativecommons.org/licenses/by/4.0/

We thank all reviewers for their careful evaluation of our manuscript and for their constructive comments. We have carefully considered all points raised and have revised the manuscript accordingly. Our point-by-point responses are provided below (in blue text). We hope that the revised manuscript is now suitable for publication in Nature Communications.

Reviewer #1 (Remarks to the Author):

The manuscript explores an interesting and potentially important role for CG14075/Mmt in starvation physiology and feeding regulation. The study combines genetic, metabolic, and imaging approaches to dissect the function of this peptide, which the authors propose acts as a novel CC-derived hormone that modulates amino acid utilization and feeding behavior under nutrient stress. While the topic is timely and of broad interest, several of the central claims are not yet fully supported by the presented data. In particular, the evidence that CG14075 is produced and secreted from the CC, functions independently of AKH, and directly regulates metabolism requires stronger experimental support. Adult-specific CC manipulations with independent RNAi lines and appropriate UAS/background controls are needed to exclude developmental effects and genetic-background artifacts. Quantitative, absolute metabolite measurements and controlled feeding/secretion assays are also essential to resolve current inconsistencies and to substantiate the central claims.

Response: We thank the reviewer for this thoughtful and constructive overall assessment. We appreciate the recognition that the study addresses an interesting and potentially important role for CG14075/Mmt in starvation physiology and feeding regulation. We also agree with the reviewer that several central claims required stronger experimental support, particularly with respect to CC origin, secretion, AKH dependence, and tissue specificity.

In response, we performed substantial additional experiments and analyses in the revised manuscript, including CC-specific rescue in the knockout background, adult-specific knockdown using independent RNAi lines, additional UAS control comparisons, improved metabolite analyses with absolute values and interaction-based statistics, explicit testing of AKH co-suppression, refined feeding and secretion assays, and expanded imaging. We believe that these additions considerably strengthen the evidence that CG14075/Mmt functions as a CC-derived endocrine factor that contributes to starvation adaptation, while also clarifying the limits of our mechanistic conclusions where direct evidence remains incomplete.

We are grateful for the reviewer's comments, which have substantially improved the rigor, clarity, and interpretability of the manuscript.

Reviewer1

1. No genetic rescue.

There is no demonstration that CC-specific re-expression of CG14075/Mmt in the knockout background restores survival or metabolic phenotypes. The authors possess UAS-CG14075, but this is used for overexpression (Tub-Gal4ts) rather than add-back in the KO background for CC-specific rescue. Demonstrating rescue is a major standard for establishing causality and would strongly validate the model.

Response: We thank the reviewer for this important comment. Genetic rescue is indeed an important standard for establishing causality.

To directly test whether CG14075/Mmt function in the corpora cardiaca (CC) contributes to the observed phenotypes, we performed CC-specific re-expression of CG14075/Mmt in the *CG14075/Mmt* knockout background using *UAS-CG14075::HA* driven by *Akh-GAL4*. We found that CC-specific re-expression partially improved starvation survival and significantly restored the accelerated TAG and glycogen depletion phenotype observed in *CG14075/Mmt* knockout animals (Exdended Data Fig. 2e, f). These results indicate that CG14075/Mmt expression in CC cells is sufficient to rescue key aspects of the mutant phenotype, supporting a causal role of CC-derived CG14075/Mmt in systemic metabolic regulation.

Although the rescue was partial, we note that the level of CG14075/Mmt restoration achieved by *UAS-CG14075::HA* in CC cells did not fully recapitulate endogenous expression levels, as evidenced by both protein and mRNA measurements (Extended Data Fig. 2g, h). In addition, the use of an HA-tagged CG14075/Mmt construct may potentially affect protein processing, stability, or secretion efficiency. Therefore, we interpret the partial rescue as likely reflecting incomplete restoration of CG14075/Mmt protein in CC cells rather than the involvement of additional tissues. We have incorporated these rescue data into the revised manuscript.

2. CC ablation versus AKH knockdown.
Initially, the authors compare Akh knockdown and CC ablation, using the stronger CC-ablation phenotype to argue that the CC contributes to starvation resistance through mechanisms beyond AKH. A more parsimonious explanation is that the knockdown does not produce a complete loss of function.

Response: We appreciate the reviewer’s point that a more parsimonious explanation for the stronger phenotype of CC ablation is incomplete loss of function in the RNAi condition. To address this directly, we quantified *Akh* mRNA levels in both conditions. We found that *Akh* RNAi reduced *Akh* mRNA by approximately 98%, while CC ablation reduced *Akh* mRNA by approximately 99% (Extended data Fig. 1a). Thus, at the transcript level, *Akh* knockdown is highly effective and comparable to CC ablation, arguing against incomplete depletion as the primary explanation.

3. CG14075 expression pattern and CC specificity.

It is unclear why CG14075 is not enriched in the CC in Fig. 1e, and the manuscript does not present clear data supporting is predominantly expressed in the CC. If the RNA-seq data are correct, this suggests that CG14075 is not CC-specific. Public expression datasets also indicate strong neuronal expression of CG14075.

Response:
We thank the reviewer for this important comment regarding *CG14075/Mmt* expression specificity.

First, in Fig. 1e we show genes whose expression is significantly reduced upon CC ablation, representing genes enriched in the CC. *CG14075/Mmt* is included among these genes. Thus, our RNA-seq and qPCR data suggest that *CG14075/Mmt* expression is largely dependent on the presence of CC cells. To clarify this point and avoid misinterpretation, we have added an annotation ("enriched in the CC") to the right side of the graph in Fig. 1e in the revised figure.

Second, to further validate tissue specificity, we performed CC-specific knockdown of *CG14075/Mmt* and measured whole-body mRNA levels. We found that systemic *CG14075/Mmt* transcript levels were reduced to approximately 3% of control levels under CC-specific RNAi (Extended data Fig. 1a). This strong reduction suggests that *CG14075/Mmt* is predominantly expressed in CC cells, as removal of CC expression nearly eliminates total transcript abundance.

Regarding public expression datasets, we note that FlyAtlas2 reports *CG14075/Mmt* expression in the "Head" or "brain" (Revision Fig.1). However, it is important to recognize that the corpora cardiaca are anatomically associated with the brain and may be included in brain dissections. Indeed, *Akh* itself—well established to be produced in the CC specific manner—is also reported as expressed in the "Head" or "brain" in FlyAtlas2 datasets (Revision Fig.2). Therefore, the apparent neuronal signal in public datasets may reflect inclusion of CC tissue rather than widespread neuronal expression.

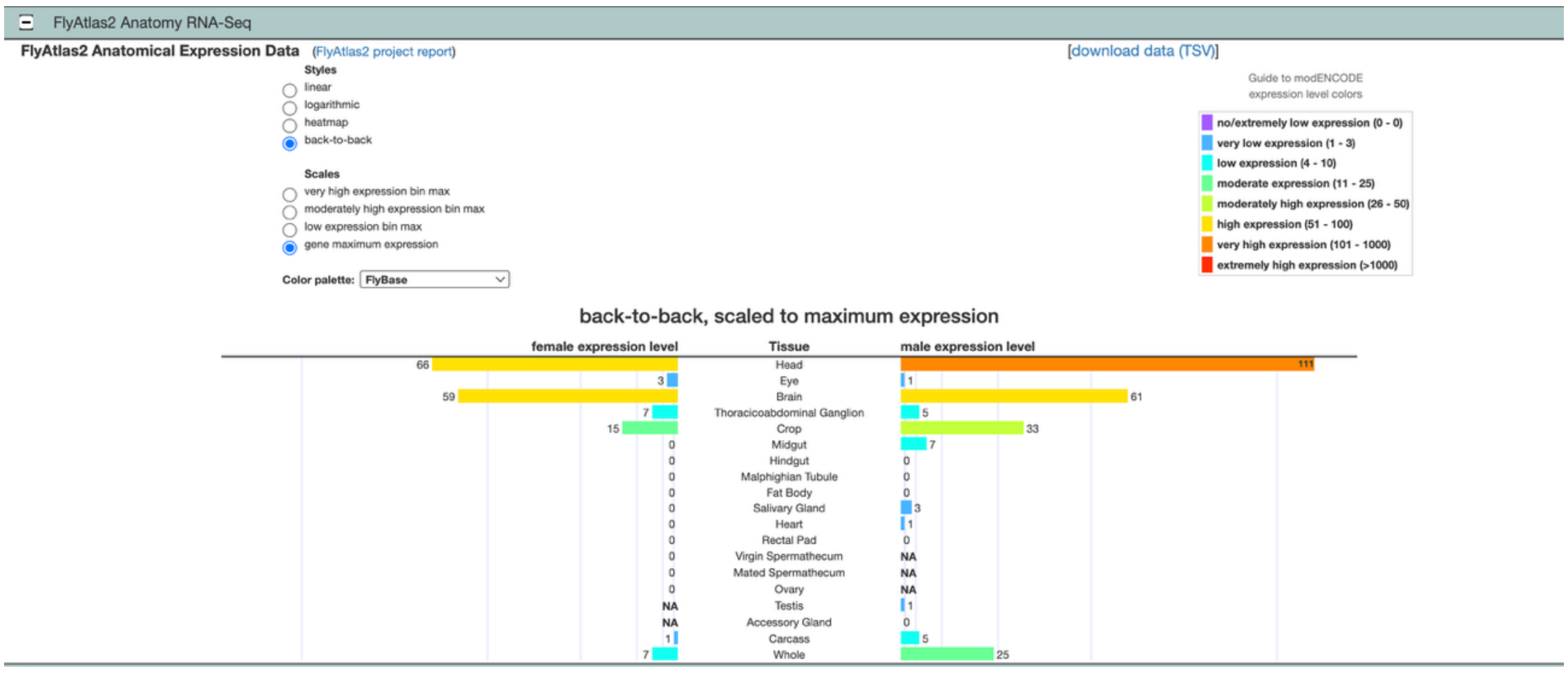

Revision Fig.1 *CG14075* expression data in FlyAtlas2
Tissue expression profile of *CG14075* from the FlyAtlas2 anatomy RNA-seq dataset (FlyBase).

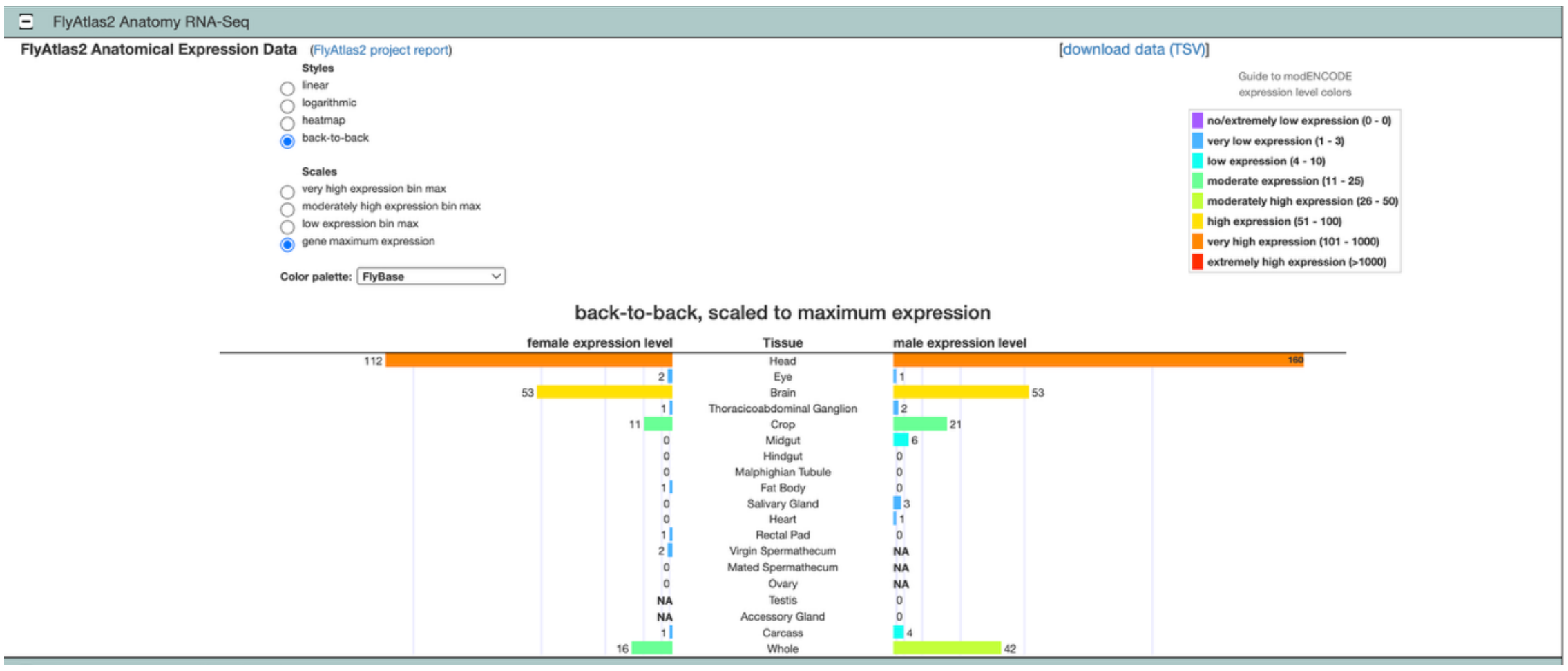

Revision Fig.2 *Akh* expression data in FlyAtlas2
Tissue expression profile of *Akh* from the FlyAtlas2 anatomy RNA-seq dataset (FlyBase).

4. CG14075 localization (Fig. 1f).
Overlap between CG14075 and AKH signals appears limited. Although CG14075 staining disappears in the null mutant, confirming the CC as the source requires repeating the experiment with CC-specific CG14075 knockdown, staining for both AKH and CG14075 to verify loss specifically in CC cells.

Response: We thank the reviewer for this helpful comment. We agree that the original image in Fig. 1f was acquired with relatively high gain, which may have obscured the localized signal in the CC and reduced the apparent overlap with AKH staining. We have

therefore replaced the original image with newly acquired images obtained under optimized gain settings, which more clearly resolve CG14075/Mmt localization in CC cells (Fig. 1f).

In addition, we have directly addressed the reviewer's suggestion by examining CC-specific *CG14075/Mmt* knockdown. As shown in Extended data Fig. 1b, CC-specific RNAi targeting *CG14075/Mmt* results in a clear loss of CG14075/Mmt immunostaining in CC cells, while AKH staining remains detectable. Conversely, CC-specific *Akh* knockdown selectively eliminates AKH signal. These results support that the CG14075/Mmt signal originates from CC cells and validate the specificity of the staining.

5. Inconsistent phenotypes and interpretation (Fig. 2a, b).
Global or CC loss of CG14075 increases starvation sensitivity, yet CC ablation increased starvation resistance and was previously used to argue for other CC-derived factors. If CC ablation removes both AKH and CG14075, the phenotype should be weaker than AKH knockdown alone. The current logic and interpretation need clarification and CG14075 cannot be that factor(s) beyond Akh.

Response: We thank the reviewer for raising this important point. We agree that the phenotypes of CG14075/Mmt loss and CC ablation appear counterintuitive, and our interpretation required clarification.

Initially, we hypothesized that CG14075/Mmt loss might attenuate the starvation-resistance phenotype caused by *Akh* knockdown, resulting in an intermediate phenotype. However, when we simultaneously knocked down *CG14075/Mmt* and *Akh* in CC cells, we instead observed that the double knockdown exhibited further increased starvation resistance, accompanied by slower depletion of TAG and more pronounced amino-acid accumulation during starvation (Fig. 6c-f). Importantly, these phenotypes resemble the metabolic profile of CC ablation, which also delays nutrient consumption and increases starvation survival (Fig.1b-d).

Based on our genetic and endocrine data, we interpret the starvation sensitivity caused by CG14075/Mmt loss as largely AKH-dependent. *CG14075/Mmt* knockdown increases AKH secretion during starvation, and the reduced starvation survival of *CG14075/Mmt* knockdown animals is suppressed by *Akh* co-knockdown (Fig. 6b, c). These results suggest that CG14075/Mmt normally acts to restrain starvation-induced AKH output, and loss of CG14075/Mmt leads to AKH hypersecretion and increased starvation sensitivity.

In contrast, our data indicate that CG14075/Mmt also contributes to amino-acid metabolism through an additional component that is not fully explained by AKH. For example, *CG14075/Mmt* knockdown causes a characteristic accumulation of threonine, which is not observed upon *Akh* knockdown alone, and the amino-acid accumulation phenotype becomes markedly stronger in the double knockdown (Fig. 6f). These observations suggest that CG14075 and AKH can influence amino-acid homeostasis through partially

independent mechanisms. We have revised the manuscript to clarify this logic and have expanded the Discussion accordingly.

Importantly, we do not consider that all aspects of the starvation-resistance phenotype observed upon CC ablation can be explained solely by CG14075/Mmt and AKH. Instead, our data support a model in which CC ablation produces a composite phenotype reflecting the simultaneous loss of multiple CC-derived endocrine outputs, including both AKH and CG14075/Mmt, and potentially additional factors. Consistent with this view, the pronounced reduction in glycogen observed in CC-ablated flies was not recapitulated by the combined knockdown of *CG14075/Mmt* and *Akh*, suggesting that the CC-ablation phenotype cannot be fully explained by the loss of these two hormones alone.

6. Metabolite measurements and normalization (Fig. 2c).
To claim that the CC is the source of metabolism-regulating CG14075, experiments in Fig. 2c should be repeated with CC-specific, adult-restricted knockdown, using independent RNAi lines and UAS controls. The current metabolite data show large variance. TAG levels appear lower at baseline in animals lacking CG14075 but decline at a similar rate during starvation. Glycogen is higher at baseline and is not depleted before TAG during starvation, which is expected. If CG14075 affects protein, normalizing metabolites to protein may be inappropriate. The authors should report absolute metabolite values and analyze depletion trajectories using two-way ANOVA (time x genotype) rather than separate t-tests. Because CG14075 likely has developmental roles, adult-specific CC knockdown is necessary to exclude developmental effects.

Response: We appreciate the reviewer's thoughtful comments on metabolite quantification, normalization, statistical analysis, and potential developmental effects.

First, to exclude developmental contributions and to test whether the metabolic phenotypes arise from CG14075/Mmt function in adult CC cells, we performed adult-restricted, CC-specific *CG14075/Mmt* knockdown. Using two independent RNAi lines, we reproduced the starvation-associated reductions in TAG and glycogen observed in the *CG14075/Mmt* mutant animals (Extended data Fig. 2g).

Second, we agree that normalization to protein may be problematic if CG14075/Mmt affects protein abundance. In our original analysis, metabolite levels were normalized to total protein. To address this concern, we now provide unnormalized (absolute) metabolite values (Extended data Fig. 2a). Importantly, total protein levels did not differ substantially between control and *CG14075/Mmt* knockout animals, and the interpretation of TAG and glycogen phenotypes was consistent with or without protein normalization.

Third, we agree that depletion trajectories should be evaluated using an interaction framework. We therefore added the metabolite time-course data using two-way ANOVA (time × genotype). This analysis revealed significant time × genotype effects for glycogen,

trehalose, and glucose ($p < 0.05$), indicating genotype-dependent differences in starvation-induced depletion. For TAG, although the trend was similar, the time-dependent depletion difference did not reach statistical significance ($p = 0.104$). We have revised the text to avoid over-interpretation of TAG depletion rate differences and to reflect the updated statistical outcomes.

7. Lipid droplet and feeding assays (Fig. 2d–g).
Lipid droplets in Fig. 2d should be quantified. For Fig. 2e–g, CAFE assays with either sugar or yeast should be added to corroborate FlyPAD data, and a dye-based intake assay with sugar or yeast would directly measure consumption. Discrepancies between FlyPAD (effect) and CAFE (no effect) must be resolved with better-controlled feeding experiments. As some "neuronal" drivers may also target the CC, CC-specific manipulations, a second RNAi line, and adult-specific designs are required.

Response: We thank the reviewer for this detailed and constructive comment. We addressed each point as follows.

First, we quantified the lipid droplet size in Fig. 2d and confirmed that lipid droplets are significantly smaller in *CG14075/Mmt* knockout flies (Fig. 2c; new quantification in Extended data Fig. 2b), supporting the conclusion that CG14075/Mmt influences lipid droplet morphology during starvation.

Second, regarding the concern about potential discrepancies between flyPAD and CAFÉ assays, we would like to clarify that our datasets do not show a major inconsistency in the overall conclusion. The apparent lack of effect in the CAFÉ assay was observed under fed conditions, whereas under starvation conditions *CG14075/Mmt* knockout animals exhibited increased food intake in both flyPAD and CAFÉ assays. Therefore, the conclusion that *CG14075/Mmt* mutants increase feeding specifically in the post-starvation context is supported by both methods.

We note that, in the flyPAD analysis, the effect on yeast consumption was not identical across genotypes: depending on the genetic background and manipulation (CC-specific RNAi knockdown versus whole-body knockout), yeast intake showed either a statistically significant difference or a similar trend of increase (Fig. 2g). This variability may reflect the contribution of CG14075/Mmt expressed outside the CC (e.g., in the nervous system, as reported in a preprint paper (Francisco et al., 2022)), which may influence yeast-related feeding decisions and lead to a stronger phenotype in the knockout than in CC-restricted knockdown. Overall, despite this quantitative variation in yeast intake, the starvation-induced increase in total feeding (and particularly sucrose intake, see below) was consistently observed in CG14075/Mmt loss-of-function animals.

Third, to determine whether the increased post-starvation feeding is driven primarily by carbohydrate versus yeast consumption, we performed separate CAFÉ assays using

sucrose-only or yeast-only solutions. We found that post-starvation intake was significantly increased for sucrose in *CG14075/Mmt* knockout flies, whereas yeast consumption showed only a upward trend that did not reach statistical significance (Extended data Fig. 3f). These results suggest that *CG14075/Mmt* knockout flies preferentially increase sugar intake after starvation, potentially to replenish depleted energy reserves. This sugar preference is also consistent with our observation that sucrose feeding strongly suppresses starvation-induced CG14075/Mmt secretion (Fig. 3g).

Finally, we agree that tissue specificity and developmental confounds must be carefully controlled. We note that *Akh-GAL4* is a CC-specific driver (Lee & Park, 2004); therefore, the manipulations reflect CC-specific effects. To further exclude potential developmental contributions, we performed adult-specific CC knockdown of *CG14075/Mmt* and tested two independent RNAi lines. Both adult-specific RNAi lines reproduced the increased post-starvation feeding phenotype (Extended data Fig. 3e), supporting the conclusion that CG14075/Mmt acts in adult CC cells to regulate feeding amount after starvation.

8. CG14075 regulation by nutrients (Fig. 3g,i).
Refeeding with peptone is of limited value because flies consume little of it (they do not like it). Testing yeast refeeding would clarify whether sugar specifically regulates CG14075 secretion. These experiments should also be repeated in CC-specific CG14075 knockdown flies to confirm the CC as the source. In Fig. 3i, showing loss of the CG14075 signal with CC-specific knockdown would strengthen the conclusion, especially since the staining pattern is atypical for a secreted peptide and lacks clear granularity. The finding that secretion occurs only after 36–48 h of starvation raises concerns about physiological relevance, as flies are close to death at this time.

Response: We thank the reviewer for this important comment regarding nutrient regulation of CG14075 secretion and the interpretation of Fig. 3g, i in the initial submission.

First, we agree that peptone refeeding can be difficult to interpret if consumption is low. To address this directly, we performed a dye-based feeding verification and confirmed that starved flies indeed consume the peptone-only medium during the refeeding window (Revision Fig.3). This indicates that the peptone refeeding condition was effective in our experimental context.

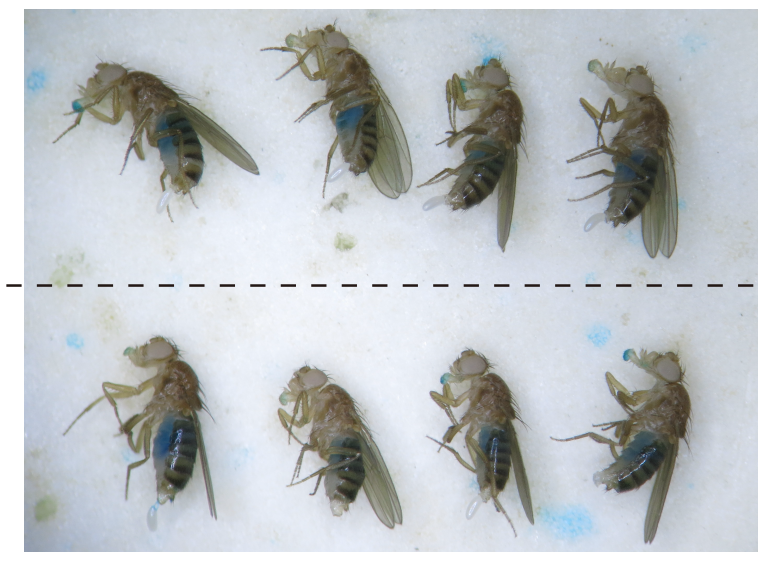

	Total # of flies	# of flies fed	% of flies fed
Sucrose	32	32	100
Peptone	28	28	100

Revision Fig.3 Refeeding with sucrose or peptone solution
Flies were starved for 24 hours and subsequently allowed to feed on either sucrose or peptone solution for 1 hour.

Second, to more systematically test which nutrients suppress CG14075/Mmt secretion, we performed additional refeeding experiments using yeast-only and coconut oil–only media (in addition to sucrose and peptone). We found that sucrose robustly suppressed CG14075/Mmt secretion, whereas yeast and coconut oil showed only a modest suppression trend that did not reach statistical significance (Fig. 3g). These results suggest that sugar is a dominant suppressive input for CG14075/Mmt secretion under our assay conditions, while other nutrient sources may have weaker or more variable effects.

Third, as requested, we repeated the starvation/refeeding secretion assays in CC-specific *CG14075/Mmt* knockdown flies. Under these conditions, CG14075/Mmt was no longer detectable in the hemolymph during starvation or after peptone refeeding (Extended data Fig. 5b). These data support the conclusion that the CC is the major source of circulating CG14075/Mmt in these experiments.

Fourth, we agree that the original CC immunostaining pattern in Fig. 3i appeared atypical for a secreted peptide and lacked clear granularity. We therefore re-imaged the CC at higher resolution and optimized gain settings. In the revised images, CG14075/Mmt is detected in discrete puncta (Fig. 3i, j), and importantly, these puncta are eliminated by CC-specific *CG14075/Mmt* knockdown (Extended data Fig. 5g), confirming that the punctate signals are CG14075/Mmt-derived. Interestingly, CG14075/Mmt puncta do not strongly overlap with AKH puncta, suggesting distinct subcellular localization within CC cells (Fig. 3j). We have incorporated these improved images and quantification into the revised figures.

Finally, we agree that emphasizing only the 36–48 h time point may give the misleading impression that CG14075/Mmt secretion occurs only at a terminal starvation stage. While secretion is maximal at 36–48 h, we detect CG14075/Mmt secretion earlier: circulating CG14075/Mmt increases progressively during starvation. Moreover, basal secretion is detectable even in fed flies. These observations are consistent with the fact that *CG14075/Mmt* knockout animals exhibit metabolic phenotypes already under fed and early

period of starved conditions (Fig. 2b, and Fig. 4a, b). We have revised the text to clarify this temporal pattern and to avoid over-emphasizing the late starvation peak.

9. Amino-acid metabolism and overexpression (Fig. 4).
CG14075 loss impairs amino-acid consumption during starvation, but this must be tested with adult-specific CC knockdown and in AKH-deficient backgrounds to determine whether effects are dependent or independent of AKH. Using Tub> for overexpression does not isolate CC-derived effects AKH> would be more appropriate. The reported 24-hour requirement for CG14075-induced amino-acid breakdown, combined with its late secretion (36-48 hours), challenges physiological plausibility. If CG14075 acts directly, shorter-term effects should be detectable. To establish mechanistic linkage to amino-acid catabolism, overexpress CG14075 while knocking down Tdh in the fat body to test whether the effect is abolished. This requires perhaps the LexA system.

Response**:** We thank the reviewer for these important comments regarding tissue specificity, AKH dependence, overexpression strategy, timing, and mechanistic linkage to amino-acid catabolism.

First, to exclude potential developmental effects, we performed adult-specific CC knockdown experiments. Adult-specific *CG14075/Mmt* RNAi in CC cells reproduced the key starvation-dependent amino-acid phenotype observed in *CG14075/Mmt* knockout and RNAi conditions (Extended data Fig. 11b), indicating that the phenotype arises during adulthood and is not solely attributable to developmental secondary effects.

Second, to test whether the amino-acid phenotype depends on AKH, we performed co-suppression of Akh. Under these conditions, the starvation-induced amino-acid accumulation phenotype was not rescued (Extended data Fig. 11b), suggesting that the effect of CG14075/Mmt loss on amino-acid homeostasis is at least partially AKH-independent.

Third, we agree that CC-specific overexpression is conceptually important. We therefore tested overexpression using *Akh-GAL4*. However, *Akh-GAL4* induced only a modest increase in *CG14075/Mmt* expression (~1.25-fold), and downstream transcriptional responses (e.g., *Gcat* induction) were correspondingly weak compared to transient-*Tub-GAL4*–driven overexpression (Extended data Fig. 7a, b). Importantly, overexpression of secreted neuropeptides in their native endocrine neurons does not necessarily increase circulating hormone levels, because peptide release is tightly regulated at the level of secretion. Indeed, overexpression of *Akh* using *Akh-GAL4* has previously been reported to have minimal metabolic impact (Lee & Park, 2004), likely due to uncoupling between transcript abundance and peptide release. Similarly, overexpression of the neuropeptide PDF in its endogenous PDF neurons does not alter circadian rhythmicity (Helfrich-Förster et al., 2000), illustrating that biosynthesis and secretion are not linearly coupled in neuroendocrine systems.

Moreover, in the AKH field, functional overexpression experiments are commonly performed in peripheral tissues such as the fat body to bypass secretion-level constraints and test sufficiency at the systemic level (Kim & Neufeld, 2015; Song et al., 2017). In this context, we used *Tub-GAL4ts* to achieve robust overexpression and assess functional sufficiency, particularly under fed conditions. We have revised the manuscript to clarify that *Tub-GAL4ts* overexpression is used as a sufficiency assay, while acknowledging the limitations of CC-specific overexpression in increasing circulating peptide output.

Fourth, regarding the timing concern, our secretion assay in wild-type flies indicates that CG14075/Mmt secretion is maximal at 36–48 h of starvation, but secretion is already elevated at early phase. Thus, CG14075/Mmt is not exclusively a late starvation factor, and its earlier secretion is consistent with the timescale of starvation-induced metabolic changes (Fig. 2b, and 4a, b).
Moreover, to address whether CG14075/Mmt can exert short-term effects, we performed an ex vivo culture assay and found that CG14075/Mmt is sufficient to induce *Bcat* up-regulation within 2 hours, supporting a rapid and potentially direct functional impact on amino-acid catabolic pathways.
Finally, we agree that epistasis experiments are important to mechanistically connect CG14075/Mmt to specific nodes in amino-acid catabolism. We therefore tested the role of Tdh in mediating the *CG14075/Mmt* overexpression phenotype. Notably, the reduction in threonine observed upon Tub^{ts}>*CG14075::HA* was rescued by *Tdh* knockdown, supporting that CG14075-driven metabolic remodeling engages Tdh-dependent amino-acid catabolism (Extended data Fig. 7d). We have incorporated these new results into the revised manuscript.

10. Relationship to AKH signaling, metabolic inconsistencies and quantifications (Figs. 5-6). Provide quantification for Figs. 5f,g and 6b (and why does the non-specific band increase in 6b?). CG14075 loss increases AKH secretion during starvation, suggesting that CG14075 effects might be mediated via AKH. This should be tested explicitly in AKH mutants or double knockdowns. It is puzzling that the double knockdown yields greater starvation resistance than AKH knockdown alone. If CG14075 opposes AKH, an intermediate phenotype would be expected. Moreover, some metabolic results contradict established literature: AKH-loss flies typically show higher TAG and higher glycogen, whereas here they show lower glycogen and no TAG elevation. These inconsistencies suggest potential measurement or background issues. Proper UAS controls (reconsidering whether UAS-LacZ-RNAi is the appropriate background) and background verification are essential. When basic metabolic data diverge from consensus, confidence in amino-acid measurements (which lack external benchmarks) is weakened.

Response: We appreciate the reviewer's thoughtful comments on quantification, genetic interactions, and the consistency of metabolic phenotypes with the established literature.

First, as suggested, we added quantification for Figs. 5f, g (Revised Fig. 5d, e), and 6b in the revised manuscript (Extended Data Fig.9c, d, and Extended Data Fig.11a). Regarding the nonspecific band in Fig. 6b, the molecular identity of this band is currently unknown. However, the volume of hemolymph loaded was kept constant across samples, and other nonspecific bands on the membrane did not show genotype-dependent changes (see source data file Fig.6). Importantly, our quantification was performed specifically on the CG14075/Mmt band (not the non-specific band), and no additional normalization was applied beyond loading equal hemolymph amounts.

Second, we agree that the relationship between CG14075/Mmt and AKH signaling should be tested explicitly. Our data indicate that CG14075/Mmt loss increases AKH secretion during starvation, supporting the idea that several CG14075/Mmt phenotypes are mediated through AKH hypersecretion. Consistent with this, we found that simultaneous knockdown of *Akh* suppresses multiple *CG14075/Mmt* RNAi-dependent phenotypes, including reduced starvation survival, altered TAG levels, and starvation-associated hyperactivity (Fig. 6c-e). These results are consistent with a model in which CG14075/Mmt acts to restrain starvation-induced AKH output, thereby influencing systemic energy homeostasis and behavior.

Third, we agree with the reviewer that under a simple antagonistic relationship, a *CG14075/Mmt* and *Akh* double knockdown might be expected to show an intermediate phenotype. However, as stated in Response 5, we observed that the double knockdown does not produce an intermediate phenotype. Instead, it displays a more severe phenotype that is closer to CC-ablation-like outcomes: compared with either single knockdown, the double knockdown exhibits stronger amino-acid accumulation during starvation and a further reduction trend in starvation-induced activity (Fig. 6e, f). These findings suggest that CG14075/Mmt and AKH may jointly contribute to endocrine output and systemic metabolic stability, and that simultaneously perturbing both may lead to a broader disruption of CC endocrine functions rather than a simple linear epistasis.

Finally, we appreciate the concern that some of our basic metabolic measurements might differ from consensus expectations. We note, however, that the magnitude and direction of AKH-loss phenotypes can depend on sex, age, and experimental context. In adult females, previous studies reported that TAG elevation upon AKH loss can be modest and that glycogen levels show limited or no changes (Gáliková et al., 2015; Wat et al., 2021). Our observations are consistent with these reports, and we also reproduce the well-established increase in starvation resistance in *Akh* knockdown animals (Fig. 1b, and Fig. 6c). In addition, to address potential background effects of the UAS-RNAi controls, we directly compared *UAS-LacZ-RNAi* and *UAS-Luciferase-RNAi* under identical adult-specific CC knockdown conditions. These two control lines did not show appreciable differences from each other, and both retained higher TAG and glycogen levels compared to *UAS-CG14075/Mmt RNAi* flies (Extended Data Fig. 3b). These results indicate that the

observed metabolic phenotypes are specific to *CG14075/Mmt* knockdown rather than attributable to the choice of UAS control background.

Together, these revisions and controls strengthen the confidence in the metabolic and amino-acid phenotypes described in Figs. 5–6.

11. Statistics and distributional assumptions (e.g., Fig. 6e).
Datasets such as activity in Fig. 6e appear non-normally distributed, yet were analyzed with Dunnett's test, which assumes normality. As also mentioned above two-way ANOVA should be used to analyze interactions rather than multiple t-tests per time point.

Response: We thank the reviewer for this important comment. After re-examining the data distribution, we agree that the activity dataset (Fig. 6e) appears not to be normally distributed and therefore should not be analyzed using Dunnett's test, which assumes normality. In addition, we agree that the effects of genotype and feeding state should be evaluated using an interaction framework rather than multiple t-tests at each time point.

Accordingly, we re-analyzed the activity data using the Scheirer–Ray–Hare test (a nonparametric two-way ANOVA alternative). This analysis revealed significant main effects of genotype ($p < 0.001$) and feeding condition ($p < 0.001$), whereas the genotype × feeding interaction was not significant ($p = 0.66131$). Based on this result, we focused on genotype comparisons within each feeding condition and performed pairwise tests using the Wilcoxon rank-sum test (Mann–Whitney U test) with appropriate multiple-comparison correction. The revised statistical results are reflected in the updated figure (Fig. 6e).

These revised analyses support our conclusions that *CG14075/Mmt* knockdown increases activity under starvation, whereas *Akh* knockdown decreases starvation-induced activity. We thank the reviewer for this suggestion, as the revised analysis allowed for a more appropriate statistical comparison across genotypes and improved the interpretability of differences between single and double knockdown conditions.

In summary, the manuscript provides a valuable dataset and a potentially important new angle on CC signaling and starvation physiology. However, the current evidence does not yet convincingly demonstrate that CG14075 is a new CC hormone acting independently of AKH to regulate metabolism and feeding. Addressing the key issues outlined above would substantially strengthen the conclusions and elevate the work to the level required for publication in a high-impact journal.

Reviewer #2 (Remarks to the Author):

The study identifies CG14075/Mmt as a novel peptide secreted from the Drosophila corpora cardiaca (CC) that promotes starvation resistance. While Akh knockdown improved survival, complete CC ablation conferred even greater protection, suggesting additional factors. RNA-seq and RT-qPCR validation pinpointed CG14075/Mmt as a CC specific peptide, whose loss led to starvation sensitivity, excessive depletion of lipid and carbohydrate stores, and elevated feeding. CG14075/Mmt is secreted in the hemolymph in response to a prolonged starvation and suppressed by sugar refeeding. The authors propose that CG14075/Mmt and Akh operate in a reciprocal feedback loop, balancing energy metabolism during starvation. Mechanistically, CG14075/Mmt promotes threonine and branched-chain amino acid catabolism by inducing Bcat and Gcat expression. Its cysteine-rich C-terminal domain is essential for function, and ex-vivo peptide treatment stimulates peripheral tissue metabolism. Loss of CG14075/Mmt or fat body threonine dehydrogenase (Tdh) impaired redox homeostasis and enhanced oxidative stress. Thus, CG14075/Mmt acts as a CC-derived hormone-like factor linking amino acid metabolism to starvation resilience. Together, these findings identify CG14075/Mmt as a novel CC-derived, hormone-like peptide that coordinates the metabolic transition from lipid/carbohydrate to amino acid utilization during prolonged starvation. The study proposes that CG14075/Mmt driven threonine catabolism in the fat body maintains redox balance and energy homeostasis, thereby promoting starvation resistance.
While the authors present compelling evidence supporting the role of CG14075/Mmt as a hormone-like peptide that mediates the starvation response through amino acid catabolism, several aspects require clarification and strengthening. The study lacks a defined molecular mechanism and does not identify a receptor or signaling pathway for CG14075/Mmt. Additionally, there is an inconsistency between the corpora cardiaca (CC) ablation phenotype and the CG14075/Mmt loss-of-function phenotype, which needs to be reconciled. Most major claims including interactions with Akh, regulation of amino acid metabolism, and redox control are based primarily on indirect or correlative evidence rather than direct functional assays.
A major shortcoming is the absence of a known receptor and undefined target tissues, leaving the hormone-like classification mechanistically incomplete. Because the authors have not identified a receptor, which we realize is non-trivial, the data presented in the manuscript need to be carefully presented/discussed considering indirect mechanisms.
Overall, this is an exciting report with a number of very interesting findings. However, the study has a number of shortcomings that requires major revisions. The following points merit further investigation:

Response: We thank the reviewer for this thoughtful and encouraging summary of our study. We greatly appreciate the reviewer’s positive assessment that our work identifies CG14075/Mmt as a novel CC-derived hormone-like factor linking amino acid metabolism to

starvation adaptation, and that the study provides an interesting framework for understanding how metabolic transitions are coordinated during prolonged nutrient deprivation.

We also appreciate the reviewer's careful evaluation of the manuscript's limitations. We agree that several of the major conclusions required clearer mechanistic framing and stronger experimental support. In response, we performed substantial additional experiments and analyses in the revised manuscript, including CC-specific rescue in the mutant background, expanded secretion and imaging analyses, direct ROS and glutathione measurements, and antioxidant rescue experiments.

In addition, we have revised the Discussion to more carefully consider the mechanistic basis of CG14075/Mmt action, including the possibility that CG14075/Mmt may function not only through a receptor-mediated pathway but also via receptor-independent mechanisms.

At the same time, we revised the text and working model to more clearly distinguish conclusions that are directly supported by our data from mechanistic possibilities that remain unresolved. We hope that these additions and revisions have substantially strengthened the manuscript and clarified the scope of our conclusions. We are grateful for the reviewer's constructive suggestions, which have significantly improved both the rigor and the presentation of this study.

1. "CG14075/Mmt is a novel secreted hormone-like peptide involved in starvation resistance"
- While CG14075/Mmt is detectable in the hemolymph, direct evidence for active secretion from the CC is limited. This could be addressed by ex vivo CC culture and measurement of Mmt release into the media, or by blocking CC-specific secretion and showing a corresponding loss of Mmt from the hemolymph. Immunostaining of secretory vesicles and their colocalization with Mmt could further support regulated secretion. Although not strictly required, CC-specific reintroduction of Mmt to rescue starvation sensitivity would strengthen the causal link. Also, have the authors considered that CG14075/Mmt is a distinct isoform from the one from the brain and plays distinct role in starvation resistance?

Response: We thank the reviewer for this thoughtful comment. We agree that direct evidence for active secretion of CG14075/Mmt from the corpora cardiaca (CC) would strengthen the interpretation of CG14075/Mmt as a hormone-like peptide.

To address this point, we functionally inhibited CC activity by expressing the inward-rectifying potassium channel Kir2.1 specifically in CC cells to suppress neuronal excitability. Under control flies, starvation induced a clear increase in circulating CG14075/Mmt levels. However, CC-specific Kir2.1 expression significantly reduced starvation-induced CG14075/Mmt levels in the hemolymph (Fig. 3h). These results indicate that

CG14075/Mmt release depends on CC cellular activity and are consistent with a regular secretion mechanism.

In addition, we revisited the subcellular localization of CG14075/Mmt in CC cells. The original images did not clearly resolve vesicle-like structures. We therefore re-imaged the CC at higher resolution and performed line-plot analyses of AKH and CG14075/Mmt signals. Under optimized imaging conditions, CG14075/Mmt exhibits punctate localization within CC cells, consistent with vesicular storage (Fig. 3i). Notably, these puncta are eliminated upon CC-specific *CG14075/Mmt* knockdown, supporting their specificity (Extended Data Fig. 5g). Interestingly, CG14075/Mmt puncta display a localization pattern distinct from AKH, suggesting separate vesicle populations or differential packaging (Fig. 3j).

Furthermore, we performed CC-specific rescue experiments in the *CG14075/Mmt* mutant background. Re-expression of CG14075/Mmt in CC cells significantly improved starvation survival (although not completely restored to wild-type levels) (Extended Data Fig. 2e) and rescued the associated metabolic phenotypes, including TAG and glycogen depletion defects and amino-acid accumulation (Extended Data Fig. 2f, and Extended Data Fig. 6c, d). These results support the conclusion that the major systemic metabolic functions of CG14075/Mmt derive from its CC expression.

Finally, regarding the potential existence of distinct isoforms between brain- and CC-derived CG14075/Mmt, we note that the presence of discrete isoforms remains unclear. However, it is possible that CG14075/Mmt undergoes tissue-specific processing or post-translational modification in the brain and CC. Consistent with this idea, the CG14075/Mmt species detected in the hemolymph appears slightly larger than the predicted molecular weight of the processed peptide lacking the signal sequence (~15–16 kDa), suggesting that CG14075/Mmt may undergo additional modification. These observations raise the possibility that functionally distinct forms of CG14075/Mmt may be generated in different tissues, with CC-derived CG14075/Mmt regulating systemic metabolic responses during starvation, and brain-derived CG14075 potentially controlling behaviors such as yeast preference. Further biochemical and molecular characterization will be required to determine the precise molecular forms of CG14075/Mmt and their tissue-specific functions.

2. “CG14075/Mmt indirectly regulates lipid and carbohydrate depletion via Akh”
- While there is a clear reduction in lipid and carbohydrate levels in Mmt mutants during starvation, the tissue specificity of Mmt remains unclear. The authors have shown a reduction in TAG in the fat body in MmtKO animals, however, experiments for fat body specific Mmt knockdown or rescue and changes in energy metabolism would be useful. The observation that Mmt mutants feed more but still lose energy reserves suggests other systemic defects not explained by Akh modulation alone. Authors have mentioned a feedback loop between Akh and Mmt and provided data for changes in systemic metabolites driving this loop. However, the feedback claim lacks functional tests. For

example, whether Mmt acts directly on CC cells to alter Akh secretion or synthesis is untested.

Response: We appreciate the reviewer's comment and agree that tissue specificity and mechanistic interpretation are critical.

To test whether CG14075/Mmt acts tissue-autonomously in the fat body, we performed fat body–specific *CG14075/Mmt* knockdown, but this manipulation did not affect starvation survival or TAG levels (Extended Data Fig. 2c, d). These results suggest that the major starvation phenotypes of *CG14075/Mmt* mutants are not simply explained by loss of CG14075/Mmt function within the fat body itself. Rather, they are more consistent with a model in which CG14075/Mmt primarily functions as a CC-derived endocrine factor.

Regarding feeding, *CG14075/Mmt* mutants increased intake specifically after starvation, whereas no significant difference was detected under fed conditions (Fig. 2f). This pattern is consistent with the accelerated depletion of glycogen and other energy reserves in *CG14075/Mmt* mutants, suggesting increased feeding as a compensatory response to starvation-induced energy depletion.

Importantly, we agree that not all *CG14075/Mmt* mutant phenotypes are explained by AKH modulation alone. While the starvation survival defect and TAG depletion phenotype were rescued by double knockdown with Akh, the starvation-induced amino-acid accumulation phenotype was not rescued and instead became stronger (Fig. 6f). These results indicate that *CG14075/Mmt* mutant phenotypes reflect a combination of AKH-dependent and AKH-independent effects.

Finally, we agree that the mechanistic basis of the proposed CG14075/Mmt–AKH feedback remains to be fully resolved. To gain preliminary insight into whether CG14075/Mmt might directly influence CC activity, we performed ex vivo live calcium imaging using GCaMP in CC cells. Under our assay conditions, addition of buffer containing CG14075/Mmt did not produce a detectable difference in GCaMP responses compared with control buffer (Revision Fig. 4). These results suggest that CG14075/Mmt does not directly trigger acute Ca^{2+} signaling in CC cells under the conditions tested.
However, this result does not exclude alternative mechanisms. CG14075/Mmt could influence CC activity through signaling modalities other than Ca^{2+} (for example cAMP-dependent pathways), or through longer-term regulatory effects that cannot be captured by short-term live imaging assays. Therefore, the precise mechanism by which CG14075/Mmt may participate in feedback regulation of AKH secretion remains unclear.

Interestingly, recent work reported that threonine can activate larval CC cells (Ahmad et al., 2025). Although technical challenges currently limit detailed live-imaging analysis in adults, it is possible that amino acids that accumulate upon CG14075/Mmt or AKH inhibition, such as threonine or BCAAs, could modulate CC activity. Investigating whether such metabolites contribute to feedback regulation of CG14075/Mmt and AKH secretion will be an important direction for future studies. We therefore revised the manuscript to describe this relationship more cautiously and to clarify that whether CG14075/Mmt acts directly on CC cells to modulate AKH secretion is an important question for future work.

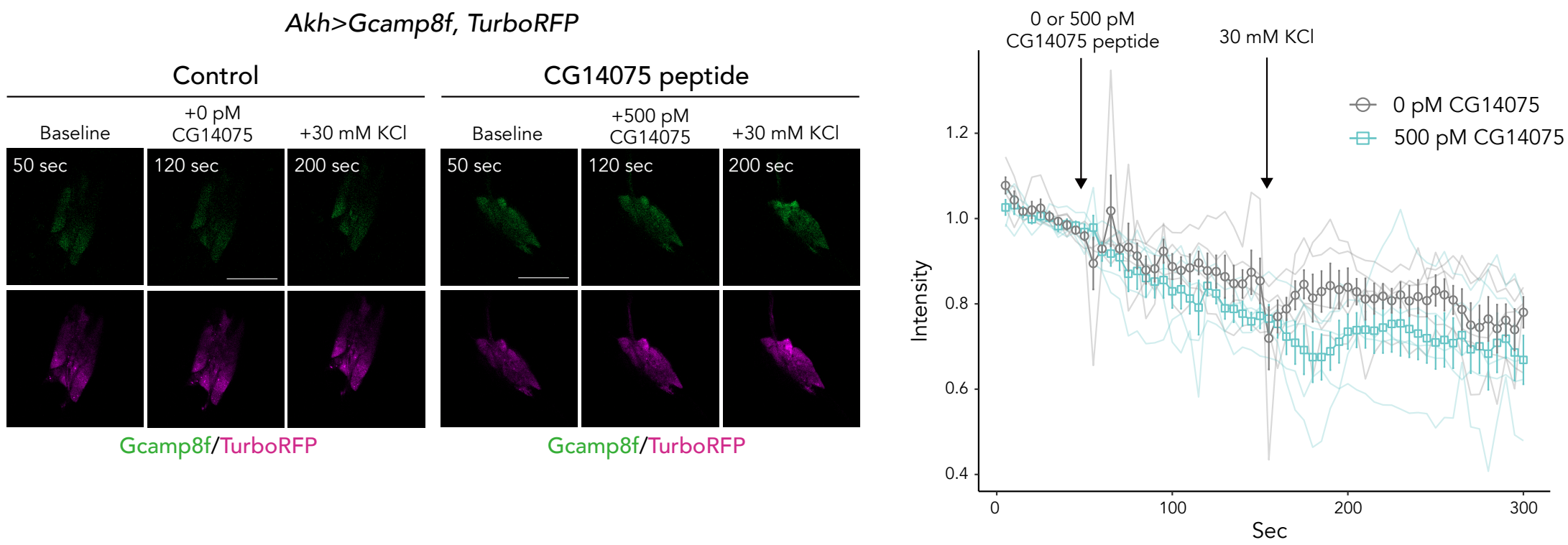

Revision Fig.4 GCaMP imaging of CC cells
Fed flies were dissected in hemolymph-like buffer and subjected to GCaMP imaging. Fifty seconds after the start of recording, buffer containing CG14075/Mmt or control buffer was added. At 150 seconds, KCl (90 mM; final concentration 30 mM) was applied. Normalized GCaMP signal intensity (TurboRFP-normalized) showed no substantial difference between the presence and absence of CG14075.

3. "CG14075/Mmt regulates amino acid metabolism"
- Changes in amino acid metabolism in CG14075/Mmt mutant flies could potentially result from a secondary transcriptional response to altered energy or redox state. A rescue experiment (CC specific reintroduction of Mmt in Mmt mutant background) would be appropriate. Low level of Mmt could be detected from the onset of starvation, so it would be appropriate to test if there is a low level of amino acid catabolism by Mmt at this stage. This might explain the lower level of TAG and glycogen in MmtKO animals from the very early stage of starvation.

Response: We thank the reviewer for this thoughtful suggestion. We agree that changes in amino-acid metabolism in *CG14075/Mmt* mutant flies could, in principle, arise indirectly from secondary transcriptional responses to altered systemic energy or redox state. To address the causal relationship and tissue specificity, we performed the rescue experiment suggested by the reviewer.

Specifically, reintroducing *CG14075/Mmt* selectively in the CC in the mutant background significantly restored the starvation-associated amino-acid phenotype, including the

abnormal amino-acid accumulation observed in *CG14075/Mmt* mutants during starvation (Extended Data Fig. 6c, d). These results support the conclusion that CC-derived CG14075/Mmt contributes to the appropriate regulation of amino-acid homeostasis during starvation.

As reviewer pointed out, low levels of CG14075/Mmt was detected at onset of starvation, indicating that CG14075/Mmt secretion begins early and could potentially contribute to amino-acid catabolism at this stage. To directly address this point, we performed additional metabolomic analyses under fed conditions and at early stages of starvation (8 h), which were not included in the previous version of the manuscript (Fig. 4a, b). Importantly, we also observed altered amino-acid profiles in *CG14075/Mmt* mutants even under fed, and early period of starvation conditions, suggesting that CG14075/Mmt may function not only as a starvation-induced factor but also at a basal physiological level to maintain metabolic homeostasis.

Consistent with this interpretation, we found that the expression of key metabolic regulators, including *Bcat* and the lipase *bmm*, is already elevated in fed *CG14075/Mmt* mutants (Extended Data Fig. 7e), indicating that compensatory transcriptional programs are engaged prior to starvation. Thus, the early depletion of TAG and glycogen in *CG14075/Mmt* mutants may reflect a combination of (i) basal metabolic dysregulation caused by loss of CG14075/Mmt-dependent control and (ii) secondary compensatory remodeling of metabolic gene expression, which together likely contribute to the complex starvation phenotype.

Collectively, these results suggest that CG14075/Mmt contributes to metabolic regulation under fed conditions, but its role becomes increasingly important during starvation, when organisms rely more heavily on amino-acid catabolism and coordinated energy homeostasis. We have revised the Results and Discussion to incorporate these points and to clarify that CG14075/Mmt-dependent metabolic regulation is active prior to starvation and becomes more prominent during nutrient deprivation.

4. "CG14075/Mmt-mediated threonine metabolism is critical for starvation resistance and oxidative stress tolerance"

- A direct relationship between CC derived CG14075/Mmt and threonine metabolism in the fat body is not well established. How does Tdh expression look like in Akh>CG14075RNAi animals? The causal link between threonine catabolism and redox balance is speculative. Only marker genes (GstD1, Sod2) were used, no direct ROS measurements or redox assays (e.g., ROS imaging, NADPH/$NADP^+$ ratio). The hypothesis that threonine catabolism maintains oxidative homeostasis would be strengthened by metabolite profiling (NADH/NAD^+, GSH/GSSG) or rescue with antioxidant treatments. The authors should also clarify how threonine catabolism contributes to starvation resistance. Is this effect primarily due to increased energy substrate availability (e.g., acetyl-CoA or TCA intermediates), modulation of oxidative stress, altered lipid metabolism, or crosstalk with signaling pathways such as mTOR or AMPK? In lines 304–305: "Tdh knockdown in the fat body led

to a reduction of TAG levels under both fed and starved conditions." This observation resembles the phenotype seen in Mmt mutants, which show reduced TAG and glycogen even in the fed state. To mechanistically link these findings, the authors could perform isotope tracing (e.g., using ^{15}N-$^{13}C_4$-threonine) in control, MmtKO, and cg>Tdh-RNAi flies to quantify the proportion of TAG derived from threonine catabolism under fed conditions. This would help clarify whether Mmt also contributes to energy storage during the fed state, in addition to its proposed role in suppressing Akh secretion during starvation.

Response: We appreciate the reviewer's comment and agree that the mechanistic link between CG14075/Mmt, threonine metabolism, and redox homeostasis requires further clarification.

First, we examined whether CG14075/Mmt regulates threonine metabolism via transcriptional control of *Tdh*. However, *Tdh* mRNA levels were not significantly altered by starvation or by CG14075/Mmt inhibition (Fig. 4d, Extended Data Fig. 7e), suggesting that CG14075/Mmt influences threonine catabolic flux through metabolic-state changes rather than direct transcriptional regulation.

Second, we directly assessed oxidative stress using CellROX imaging, as presented in the initial submission, which revealed elevated ROS levels in both *CG14075/Mmt* mutant and fat body–specific *Tdh* knockdown flies (Fig. 5e, Extended Data Fig. 9d). As suggested by the reviewer, we further expanded our analysis for performing redox metabolite profiling. Under starvation, *CG14075/Mmt* mutant and fat body *Tdh* knockdown flies showed a strong increase in oxidized glutathione (GSSG) (Extended Data Fig. 10c, d). Total GSH levels were also increased; however, the GSSG/GSH ratio showed only a modest increasing trend under starvation. The increase in GSH did not appear to depend on elevated expression of glutathione synthetic enzymes (Extended Data Fig. 10e, f) but may instead reflect compensatory glutathione production in response to increased ROS levels. Together, these results suggest that both CG14075/Mmt loss and impaired threonine catabolism are associated with altered redox balance during starvation and lead to elevated ROS levels.

Third, to test whether oxidative stress is causally linked to starvation sensitivity, we performed rescue experiments using the antioxidant vitamin C. We found that starvation survival of *CG14075/Mmt* mutant or *Tdh* RNAi flies was significantly improved by vitamin C supplementation (Extended Data Fig. 10a, b). Moreover, *Sod2* overexpression in the fat body of *CG14075/Mmt* mutants suppressed ROS levels and partially restored starvation resistance and TAG depletion phenotypes (Fig. 5g–i). These results support the interpretation that oxidative stress contributes, at least in part, to starvation sensitivity downstream of CG14075/Mmt loss.

Forth, we agree that threonine catabolism could influence starvation response through multiple, non-mutually exclusive mechanisms, including provision of metabolic

intermediates, modulation of lipid metabolism, and maintenance of redox homeostasis. We therefore revised the Discussion to explicitly present this as a multifactorial model rather than attributing the phenotype solely to one mechanism.

Fifth, regarding signaling pathways, we assessed mTOR and AMPK activity. In *CG14075/Mmt* knockout flies, p-S6K/αTubulin and p-AMPK/AMPK ratios were not detectably altered relative to controls across fed and starved conditions (Extended Data Fig. 10g). In contrast, fat body *Tdh* knockdown showed stronger effects on these signaling readouts (Extended Data Fig. 10h). Thus, CG14075/Mmt loss and *Tdh* knockdown share key metabolic phenotypes but may do so through partially distinct mechanisms. We now clarify this point in the revised text.

Finally, we agree that isotope tracing using labeled threonine would be a powerful approach to quantify threonine contribution to lipid synthesis under fed conditions. However, given the scope of the current revision, we have instead strengthened the mechanistic framework through direct ROS/redox measurements and genetic/chemical rescue experiments. We highlight lipogenesis and mitochondrial homeostasis by threonine-catabolism as an important future direction.

5. Need to clarify the CC ablation procedure. Exact time of UAS-grim induction and how long did it take to achieve ablation. Also, did you try any other procedure for ablation. Was it a complete or partial ablation? Please include ablation data in the supplement section.

Response: We thank the reviewer for this comment. We agree that the CC ablation procedure should be described more clearly and validated explicitly in the manuscript.

In our CC ablation experiments, *UAS-grim* was expressed throughout development under CC-specific Gal4 control, such that CC cells were eliminated during development. We chose *UAS-grim* because previous work reported that grim can ablate CC cells more efficiently than *UAS-reaper* (Yamada et al., 2018).

To assess the extent of ablation, we quantified whole-body transcript levels and found that both *Akh* and *CG14075/Mmt* mRNA were reduced to ~1% of control levels (Extended Data Fig. 1a). In addition, immunostaining confirmed that AKH and CG14075/Mmt signals were no longer detectable at the anatomical location of the CC in ablated animals (Extneded Data Fig. 1b). Together, these results indicate that the genetic ablation produces a near-complete (functionally complete) loss of CC.

We have revised the Methods section to explicitly state the timing and design of grim induction.

6. Have the authors tried inducing starvation like condition by genetic manipulations of either insulin and TOR signaling pathway and measuring hemolymph CG14075/Mmt

levels? This would help further establish the point that the lack of systemic energy level induces the secretion of CG14075/Mmt.

Response: We thank the reviewer for this insightful suggestion. To test whether reduced systemic energy signaling is sufficient to induce CG14075/Mmt secretion, we expressed a dominant-negative form of mTOR (mTOR-DN) specifically in the fat body to genetically mimic a starvation-like state.

However, fat body–specific mTOR inhibition did not induce CG14075/Mmt secretion into the hemolymph (Extended Data Fig. 5c). These results suggest that reduced TOR activity in the fat body alone is not sufficient to trigger CG14075/Mmt release. Therefore, CG14075/Mmt secretion likely requires additional starvation-associated signals beyond simple suppression of mTOR signaling.

7. In order to establish the point made at line 208-209 “CG14075/Mmt secretion is sensitive to carbohydrate availability, not directly to TAG levels.”, have you tried feeding starved animals a high fat diet and measuring changes in of CG14075/Mmt level?

Response: We thank the reviewer for this important suggestion. To test whether CG14075/Mmt secretion responds to dietary fat, we performed refeeding experiments using a coconut oil–based high-fat diet following starvation.

We found that coconut oil refeeding resulted in only a partial and modest suppression of circulating CG14075/Mmt levels, and the effect was substantially weaker than that observed with sucrose refeeding (Fig. 3g). These results indicate that while lipid availability may exert some influence, carbohydrate availability is a stronger suppressive signal for CG14075/Mmt secretion.

8. Although the authors have provided evidence for lipid loss in CG14075/Mmt mutant animals both in fed and starved condition, more experiments are needed to substantiate the claim that CG14075/Mmt suppresses lipolysis (line 336).

Response: We thank the reviewer for this important comment. We agree that our data do not provide direct evidence that CG14075/Mmt suppresses lipolysis per se.

We observed that expression of the lipase gene *bmm* is elevated in *CG14075/Mmt* knockout animals under both fed and starved conditions (Extended Data Fig. 7e), consistent with an enhanced lipolytic state. However, we did not directly measure lipolytic flux. Therefore, we have revised the manuscript to avoid stating that CG14075/Mmt directly suppresses lipolysis (Line 429-431). Instead, we now describe CG14075/Mmt as contributing to lipid homeostasis during starvation via Akh signaling and amino acid catabolism.

9. Need better characterization of the Akh and Mmt role in amino acid catabolism to substantiate the claim in line 360-361 “CG14075/Mmt and AKH act in concert to regulate starvation dependent amino acid metabolism”

Response: We thank the reviewer for this important comment. We agree that the relationship between CG14075/Mmt and AKH in amino-acid metabolism required clearer presentation.

To address this, we now provide a comprehensive heatmap summarizing amino-acid changes in *Akh* knockdown, *CG14075/Mmt* knockdown, and co-suppression conditions under both fed and starved states (Fig. 6f). This analysis reveals that Akh and CG14075/Mmt regulate overlapping but distinct subsets of amino acids. Several essential amino acids, as well as glycine and serine, are further elevated in the co-suppression condition compared to single knockdowns, suggesting additive or cooperative effects in specific metabolic contexts.

At the same time, certain amino acids show genotype-specific regulation, indicating that CG14075/Mmt and AKH do not function identically in amino-acid catabolism. However, because the current data are not sufficient to conclusively demonstrate cooperative regulation, we interpret these results more conservatively as indicating that CG14075/Mmt and AKH act in parallel to influence amino-acid metabolism under starvation conditions. We have therefore revised the manuscript to clarify that CG14075/Mmt and AKH exhibit partially overlapping and context-dependent roles in regulating starvation-dependent amino-acid metabolism, rather than implying a single linear or fully redundant pathway (L468-470).

Minor comments:
- Is CG14075/Mmt expressed during the larval stage as well? Do MmtKO mutants develop into adults with normal morphology and viability comparable to wild-type animals?

Response: We thank the reviewer for this comment. CG14075/Mmt is also expressed in the corpora cardiaca during the larval stage. However, CG14075/Mmt mutants develop into adults at a frequency comparable to wild-type animals and do not exhibit obvious morphological abnormalities (Extended Data Fig. 1d). These observations indicate that loss of CG14075/Mmt does not cause major developmental defects under standard laboratory conditions.

- Figure 1g, add an arrow to point the band in the WB that corresponds to CG14075/Mmt

Response: We thank for the suggestion. We have added an arrow in Fig. 1g to indicate the band corresponding to CG14075/Mmt in the western blot. This arrow marks the band at the expected molecular weight of CG14075/Mmt based on its predicted size.

All detected bands correspond to HA-tagged CG14075/Mmt; however, multiple bands are observed, likely reflecting post-translational modifications. When expressed in HEK cells, CG14075/Mmt appears as multiple bands, suggesting heterogeneous processing or modification of the protein.

Thus, the arrow indicates the band closest to the predicted molecular weight, while the additional bands likely represent modified forms of CG14075/Mmt rather than unrelated proteins.

- Figure 3i- need to add better quality image. Punctate structures are not clearly visible, and they do not match their localization profile described in the text.

Response: We thank the reviewer for the comment. We replaced the image in Fig. 3i with a higher-resolution image in which the punctate structures are more clearly visible. In addition, we included a line intensity plot to demonstrate that the CG14075/Mmt and AKH puncta exhibit distinct localization patterns.

- Extended data fig 3h, i is missing- please add them during next submission

Response: We thank the reviewer for pointing this out. We have now added the missing source data corresponding to Extended Data Fig. 3h and 3i (Revised; Extended Data Fig. 5h).

- Figure 6b, need quantification of CG14075 in Akh-RNAi and AkhR-RNAi animals.

Response: We thank the reviewer for the suggestion. We have added quantification of CG14075/Mmt levels in *Akh*-RNAi and *AkhR*-RNAi animals in the revised Extended Data Fig. 11a.

- Figure 6g, need to adjust the model figure as there is not enough evidence for CG14075 to suppress lipolysis and glycogenolysis.

Response: We thank the reviewer for the suggestion. We have revised the model in Fig. 6g and removed the direct inhibitory links between CG14075/Mmt and lipolysis/glycogenolysis. Based on our data, these effects are more likely mediated indirectly through AKH signaling and changes in amino-acid metabolism.

- Line 231-232, it would be appropriate to mention that there is a lower level of CG14075 presence during very early stages of starvation and it goes up with energy storage depletion.

Response: We thank the reviewer for this suggestion. We agree that the previous wording could be misleading. We have revised the text to clarify that circulating CG14075/Mmt increases gradually during starvation related to Fig. 3c, d.

- Line 234, need citation here- “During prolonged starvation, organisms increasingly mobilize amino acids to support gluconeogenesis and ketogenesis”

Response: We thank the reviewer for the suggestion. We have added an appropriate citation to support this statement in the revised manuscript.

- Figure 4a, a vertical line on the x-axis at value 1 to show the changes in amino acid levels more clearly.

Response: We thank the reviewer for the suggestion. We agree that the original panel made it difficult to clearly visualize increases or decreases in individual amino acids. Therefore, we revised Fig. 4a and now present the starvation-dependent changes of each amino acid using line plots, which more clearly illustrate their dynamic changes.

Reference

Ahmad, M., Wu, S., Luo, S., Shi, W., Guo, X., Cao, Y., Perrimon, N., & He, L. (2025). Dietary amino acids promote glucagon-like hormone release to generate global calcium waves in adipose tissues in Drosophila. *Nature Communications*, *16*(1), 247.

Francisco, A. P., Tastekin, I., Fernandes, A. B., Ezra-Nevo, G., Deplancke, B., Oliveira-Maia, A. J., Gontijo, A. M., & Ribeiro, C. (2022). *marmite*defines a novel conserved neuropeptide family mediating nutritional homeostasis. In *bioRxiv* (p. 2022.12.12.520095). https://doi.org/10.1101/2022.12.12.520095

Gáliková, M., Diesner, M., Klepsatel, P., Hehlert, P., Xu, Y., Bickmeyer, I., Predel, R., & Kühnlein, R. P. (2015). Energy homeostasis control in Drosophila adipokinetic hormone mutants. *Genetics*, *201*(2), 665–683.

Helfrich-Förster, C., Täuber, M., Park, J. H., Mühlig-Versen, M., Schneuwly, S., & Hofbauer, A. (2000). Ectopic expression of the neuropeptide pigment-dispersing factor alters behavioral rhythms in Drosophila melanogaster. *The Journal of Neuroscience: The Official Journal of the Society for Neuroscience*, *20*(9), 3339–3353.

Kim, J., & Neufeld, T. P. (2015). Dietary sugar promotes systemic TOR activation in Drosophila through AKH-dependent selective secretion of Dilp3. *Nature Communications*, *6*(1), 6846.

Lee, G., & Park, J. H. (2004). Hemolymph sugar homeostasis and starvation-induced hyperactivity affected by genetic manipulations of the adipokinetic hormone-encoding gene in Drosophila melanogaster. *Genetics*, *167*(1), 311–323.

Song, W., Cheng, D., Hong, S., Sappe, B., Hu, Y., Wei, N., Zhu, C., O’Connor, M. B., Pissios, P., & Perrimon, N. (2017). Midgut-Derived Activin Regulates Glucagon-like Action in the Fat Body and Glycemic Control. *Cell Metabolism*, *25*(2), 386–399.

Wat, L. W., Chowdhury, Z. S., Millington, J. W., Biswas, P., & Rideout, E. J. (2021). Sex determination gene transformer regulates the male-female difference in Drosophila fat storage via the adipokinetic hormone pathway. *ELife*, *10*(e72350). https://doi.org/10.7554/eLife.72350

Yamada, T., Habara, O., Kubo, H., & Nishimura, T. (2018). Fat body glycogen serves as a metabolic safeguard for the maintenance of sugar levels in Drosophila. *Development (Cambridge, England)*, *145*(6), dev158865.

We thank the editor and reviewers for their continued evaluation of our manuscript and for their constructive comments on the revised version. We are grateful that Reviewer 1 considered the manuscript substantially improved and suitable for publication, and we thank Reviewer 2 for acknowledging that the additional experiments strengthened the evidence supporting CG14075/Mmt as a CC-derived secreted factor involved in starvation resistance and systemic metabolic regulation.

In response to the remaining comments, we have further revised the manuscript to clarify the scope of our conclusions, moderate statements that were not directly supported by the current data and explicitly acknowledge unresolved mechanistic questions. Our point-by-point responses are provided below in blue text. We hope that the revised manuscript is now suitable for publication in Nature Communications.

Reviewer #1 (Remarks to the Author):

The authors have adequately addressed my concerns, and the revised manuscript is significantly improved and suitable for publication.

Response: We thank Reviewer 1 for the positive assessment of our revised manuscript. We are pleased that the reviewer considers our responses adequate and the manuscript suitable for publication.

Reviewer #2 (Remarks to the Author):

The authors have revised the manuscript and performed additional experiments that strengthen the evidence supporting CG14075/Mmt as a CC-derived secreted factor involved in starvation resistance and systemic metabolic regulation. In particular, the CC-specific rescue experiments and functional inhibition of CC activity provide stronger support for a CC origin of Mmt. However, several important conceptual and mechanistic issues remain unresolved, including the lack of identification of the Mmt receptor and target tissue, and the incomplete understanding of how Mmt regulates amino acid metabolism and interacts with AKH signaling, and how systemic energy depletion regulates Mmt secretion.

Response: We thank Reviewer 2 for acknowledging that the additional experiments strengthen the evidence for CG14075/Mmt as a CC-derived secreted factor involved in starvation resistance and systemic metabolic regulation.

We agree that several mechanistic questions remain unresolved, including the identity of the CG14075/Mmt receptor, its direct target tissues, its downstream signalling pathway, the relationship between CG14075/Mmt and AKH signalling, and the upstream cues controlling CG14075/Mmt secretion during starvation. We have revised the manuscript to state these limitations more clearly and to moderate conclusions that are not directly demonstrated by the current data.

Specifically, we now describe abdominal peripheral tissues, including the abdominal carcass/fat body, as candidate responsive tissues rather than established direct targets. We also explicitly note that some metabolic phenotypes may reflect secondary systemic responses, and we describe the CG14075/Mmt–AKH relationship as a functional interaction rather than a defined feedback mechanism. We have also added additional analyses and revised the text to clarify the interpretation of threonine metabolism, oxidative stress, and starvation-like genetic manipulations. These revisions do not alter the central conclusion that CC-derived CG14075/Mmt contributes to systemic metabolic adaptation during starvation, but they more clearly distinguish this conclusion from unresolved mechanistic questions.

1. Secretion of CG14075/Mmt from CC

Assessment: Partially addressed

The authors provide improved evidence for CC-dependent secretion by functionally inhibiting CC activity (Kir2.1), which reduces starvation-induced Mmt levels in the hemolymph. In addition, CC-specific rescue of Mmt in mutant animals restores starvation resistance and metabolic phenotypes, supporting a CC-derived systemic role.

However, the subcellular localization of Mmt remains unclear (Fig. 3i). While line plot analyses suggest punctate structures, the imaging data lack sufficient resolution to convincingly demonstrate distinct localization or secretion dynamics. Higher-resolution images and co-localization with established secretory vesicle markers would substantially strengthen this claim.

Additionally, the possibility of distinct Mmt isoforms (e.g., CC vs brain-derived) should be acknowledged and discussed.

Response: We have revised the manuscript to avoid concluding that CG14075/Mmt and AKH are stored in distinct vesicle populations or that their secretion dynamics differ at the subcellular level.

We also replaced Fig. 3i with higher-resolution Airyscan images of CG14075/Mmt and AKH in CC cells. These images more clearly show spatially heterogeneous, punctate-like signals for both peptides, and line plot analysis indicates that their intensity profiles are not fully identical. We now interpret these data more cautiously, stating only that CG14075/Mmt and AKH may show differences in subcellular distribution within CC cells. We have also moderated the previous statement that CG14075/Mmt localizes near the plasma membrane whereas AKH is broadly distributed throughout the cytoplasm. The revised manuscript now states that CG14075/Mmt and AKH may be differentially organised within CC cells, without assigning them to distinct vesicle populations or specific secretion mechanisms. Because these imaging data are intended as supportive evidence for subcellular organization rather than definitive evidence for vesicle identity, we have avoided making conclusions about specific secretory compartments.

In addition, we revised the Discussion to acknowledge that although current annotations do not support distinct CG14075/Mmt splice isoforms, brain- and CC-derived CG14075/Mmt may differ through peptide processing, post-translational modification, secretion dynamics, or local availability. The manuscript now discusses the possibility that brain- and CC-derived CG14075/Mmt fulfil distinct physiological roles, and that future tissue-specific transcript and peptide analyses will be needed to determine whether they represent identical or distinct molecular species.

2. Role of Mmt in systemic energy metabolism and interaction with AKH
Assessment: Partially addressed
The absence of phenotypic effects upon fat body–specific knockdown supports the conclusion that Mmt is unlikely to originate from the fat body and is instead secreted from the CC.
However, the downstream target tissue and mechanism of action remain unclear. Additional experiments—such as tissue-specific rescue (e.g., fat body reintroduction of Mmt in mutant background)—would strengthen the model of systemic action.
The proposed feedback interaction between Mmt and AKH remains insufficiently defined.
The authors have conducted ex vivo live calcium imaging experiments indicating that Mmt

does not directly regulate its own secretion from the CC. Functional evidence demonstrating whether Mmt directly regulates AKH secretion or synthesis is still lacking

Response: We agree with Reviewer 2 that the downstream target tissue and mechanism of action of CG14075/Mmt remain unresolved. Although fat body-specific reintroduction of CG14075/Mmt could be considered, this experiment would be difficult to interpret unambiguously because CG14075/Mmt is a secreted factor. Expression in the fat body could lead to ectopic secretion and systemic distribution, rather than demonstrating fat body-autonomous action or identifying the fat body as the direct physiological target tissue.

Our ex vivo culture experiments showed that CG14075/Mmt peptide alters amino acid metabolic gene expression in abdominal peripheral tissues, including the abdominal carcass/fat body, suggesting that these tissues are candidate responsive tissues. However, these data do not identify the direct in vivo target tissue, receptor, or downstream signalling pathway. We have therefore revised the Discussion to state this limitation explicitly and to note that some metabolic phenotypes may reflect secondary systemic responses to altered energy balance, feeding behaviour, amino acid accumulation, or AKH secretion.

Regarding the CG14075/Mmt–AKH relationship, our current data do not demonstrate direct regulation of AKH secretion or synthesis by CG14075/Mmt. We have therefore revised the manuscript to describe this relationship as a functional interaction between the two hormonal pathways during starvation, rather than a direct feedback mechanism. Specifically, the revised text states that perturbation of one hormonal pathway is associated with altered secretion of the other, without concluding that CG14075/Mmt directly regulates AKH secretion or synthesis.

3. Regulation of amino acid metabolism by Mmt
Assessment: Partially addressed
CC-specific rescue experiments strengthen the conclusion that Mmt contributes to starvation-associated metabolic regulation. The authors also show that Mmt is present at basal levels and may function under fed conditions.
However, the tissue specificity of Mmt function remains unclear, and it is still difficult to distinguish whether the observed metabolic changes are direct effects of Mmt or secondary systemic responses.

Response: The reviewer raises an important point regarding the tissue specificity of CG14075/Mmt function and the possibility that the metabolic changes observed in CG14075/Mmt mutants may include secondary systemic responses. The CC-specific rescue experiments support a role for CC-derived CG14075/Mmt in starvation-associated metabolic regulation, and the detection of basal circulating CG14075/Mmt is consistent with a possible function under fed conditions. However, these experiments do not identify the direct target tissue or distinguish direct peptide action from indirect systemic effects.

As noted above, ex vivo peptide treatment suggests that abdominal peripheral tissues can respond to CG14075/Mmt, but this does not establish them as direct in vivo targets. We have therefore revised the manuscript to describe these tissues as candidate responsive tissues and to state that some metabolic phenotypes may reflect secondary systemic responses to altered energy balance, feeding behaviour, amino acid accumulation, or AKH secretion. Receptor identification and tissue-specific signalling analyses will be required to distinguish direct actions from indirect systemic effects.

4. Link between Mmt and threonine metabolism / oxidative stress
Assessment: Not fully addressed
A direct mechanistic link between CC-derived Mmt and fat body–specific threonine catabolism remains lacking. While the authors show altered amino acid metabolism in Mmt mutants and Tdh knockdown animals, these datasets are not directly connected mechanistically.

The rescue of starvation sensitivity and TAG levels by antioxidant interventions (e.g., Sod2 overexpression, vitamin C) suggests that oxidative stress contributes to the phenotype. However, fat body–specific rescue experiments—such as reintroduction of Mmt or Tdh—to directly test their role in mitigating oxidative stress are still lacking.

Furthermore, experiments directly testing how threonine catabolism contributes to starvation resistance (e.g., metabolic flux or energy production) are still missing, although the authors appropriately acknowledge this limitation.

Response: We agree that the mechanistic link between CC-derived CG14075/Mmt, threonine metabolism, and oxidative stress remains incompletely resolved. We did not perform fat body-specific *Tdh* overexpression in the *CG14075/Mmt* mutant background

because *Tdh* expression is not reduced in *CG14075/Mmt* mutants, whereas *Gcat* induction is impaired. *Tdh* overexpression would therefore be difficult to interpret as a specific rescue of the CG14075/Mmt-dependent defect.

To examine the relationship between amino acid accumulation, oxidative stress, and TAG depletion, we performed fat body-specific *Sod2* overexpression in *CG14075/Mmt* mutants. *Sod2* overexpression partially rescued starvation sensitivity and TAG depletion and reduced oxidative stress, but did not suppress the accumulation of threonine, BCAAs, or other amino acids (Extended Data Fig. 11a, b). These results suggest that oxidative stress contributes to starvation sensitivity and excessive TAG consumption without correcting the underlying amino acid accumulation, and may act downstream of, or in parallel with, broader amino acid metabolic defects.

We also performed isotope-labelled threonine tracing to assess how threonine catabolism contributes to starvation adaptation. Fat body-specific *Tdh* knockdown reduced label incorporation from threonine into glycine and serine and decreased labelled fumarate, supporting the idea that Tdh-dependent threonine catabolism links threonine-derived carbon to glycine/serine metabolism and TCA-cycle-associated metabolites during starvation (Extended Data Fig. 9e). However, CG14075/Mmt loss also broadly affects BCAAs and other essential and non-essential amino acids. We have therefore revised the Discussion to state that threonine metabolism is one component of a broader CG14075/Mmt-regulated amino acid metabolic programme, and that the roles of individual amino acid species during starvation remain to be defined.

5. CC ablation procedure
Assessment: Addressed
The authors have clarified the experimental details.

Response: We thank the reviewer for noting that this point has been addressed.

6. Induction of starvation-like states
Assessment: Partially addressed
The authors attempted to induce a starvation-like condition using fat body–specific DN-mTOR expression; however, this did not trigger Mmt secretion. It remains unclear whether this manipulation effectively induces a systemic starvation state. Alternative approaches

should be considered to more robustly test this hypothesis.

Response: We thank the reviewer for this important comment. We agree that fat body-specific mTOR inhibition may not fully recapitulate the complex systemic physiological state induced by starvation, which involves coordinated endocrine, metabolic, and neuronal responses across multiple tissues. Thus, suppression of a single nutrient-sensing pathway in the fat body alone may be insufficient to reproduce the integrated starvation-induced physiological conditions required for CG14075/Mmt secretion.

We have revised the manuscript to interpret this result more conservatively, stating that reduced mTOR signalling in the fat body alone is not sufficient to induce CG14075/Mmt secretion. We also acknowledge that additional genetic, physiological, or dietary manipulations that more broadly mimic starvation will be required to define the upstream systemic cues controlling CG14075/Mmt secretion, which will be an important direction for future studies.

7. Carbohydrate vs TAG regulation of Mmt secretion
Assessment: Addressed
This point has been addressed (Fig. 3g).

Response: We thank the reviewer for noting that this point has been addressed.

8. Role in lipolysis
Assessment: Addressed
The authors have appropriately toned down their claims and now describe Mmt as contributing to lipid homeostasis rather than directly suppressing lipolysis. However, the lack of a clear mechanistic link between Mmt, AKH signaling, and amino acid metabolism should be explicitly acknowledged.

Response: We appreciate the reviewer's comment. We have revised the manuscript to avoid describing CG14075/Mmt as directly suppressing lipolysis and now state more cautiously that CG14075/Mmt contributes to lipid homeostasis during starvation. We also explicitly acknowledge that the mechanistic relationship between CG14075/Mmt, AKH signalling, and amino acid metabolism remains unresolved. In the revised Discussion, we describe the CG14075/Mmt–AKH relationship as a functional interaction rather than a defined feedback pathway, and state that the observed metabolic phenotypes may involve

indirect systemic responses through altered energy balance, amino acid accumulation, or AKH secretion. Future identification of the CG14075/Mmt receptor, target tissues, and downstream signalling pathways will be required to determine whether, and how, these endocrine and metabolic pathways are mechanistically connected.

9. Interaction between Mmt and AKH in amino acid metabolism
Assessment: Partially addressed
Additional data suggest an additive effect of Mmt and AKH on amino acid catabolism during starvation. However, the interaction between these pathways remains incompletely characterized.
The authors may consider moderating their conclusions to reflect this limitation. For example, replacing: "...restraining excessive AKH-dependent responses and promoting amino acid catabolism"
with: "...promoting amino acid catabolism"

Response: The interaction between CG14075/Mmt and AKH in amino acid metabolism remains incompletely characterized. Although simultaneous inhibition of CG14075/Mmt and AKH suggests that both pathways influence amino acid metabolism during starvation, our data do not define the mechanistic relationship between them.

We have therefore moderated the conclusion as suggested. In the revised manuscript, we no longer state that CG14075/Mmt restrains excessive AKH-dependent responses in the context of amino acid metabolism. Instead, we describe CG14075/Mmt as contributing to starvation adaptation by promoting amino acid catabolism, and we explicitly state that the mechanistic relationship between CG14075/Mmt and AKH signalling remains unresolved.

Additional comments:

Lines 494–495: The authors state that "Our data indicate that CG14075/Mmt primarily promotes the degradation of threonine and BCAAs." However, based on Figures 4a and 6f, while threonine catabolism appears to be most strongly affected in Mmt mutants, the degradation of several other amino acids is also significantly altered. Therefore, it would be more accurate to conclude that Mmt broadly influences amino acid catabolism, with threonine metabolism being the most prominently affected.

Response: We have revised the text to avoid implying that CG14075/Mmt acts only or primarily on threonine and BCAA degradation. The revised sentence now states that CG14075/Mmt broadly influences amino acid catabolism during starvation, with particularly prominent effects on threonine and BCAA metabolism.

Line 532: The authors claim that "We further found that CG14075/Mmt and AKH negatively regulate each other's secretion." However, no conclusive evidence is presented to support this statement, and this claim requires further experimental validation or clarification.

Response: We appreciate the reviewer's careful evaluation. In the revised manuscript, we no longer state that CG14075/Mmt and AKH negatively regulate each other's secretion. Instead, we now describe the relationship more cautiously as a functional interaction between CG14075/Mmt and AKH signalling during starvation. Specifically, we state that perturbation of one hormonal pathway is associated with altered secretion of the other, without concluding reciprocal negative regulation or a direct feedback mechanism.

Lines 534–535: The authors state that "this AKH-dependent suppression of CG14075/Mmt secretion appears to be mediated via fat body AkhR." Again, there is insufficient evidence provided to substantiate this conclusion, and this point should be addressed more rigorously.

Response: We agree with the reviewer that the original statement was too strong. Our data show that fat body-specific *AkhR* knockdown is associated with increased circulating CG14075/Mmt levels during starvation, but this does not establish that fat body AkhR directly mediates suppression of CG14075/Mmt secretion. We have therefore revised the manuscript to avoid implying a defined feedback pathway from the fat body to the CC. The revised text now states that the increase in circulating CG14075/Mmt upon fat body *AkhR* knockdown is consistent with the possibility that peripheral AKH/AkhR signalling influences CG14075/Mmt levels, but does not establish a direct feedback mechanism. We also acknowledge that indirect systemic metabolic responses, including changes in amino acid levels, may contribute to altered CC endocrine output.

Lines 548–550: The authors claim that "CG14075/Mmt is localized near the plasma membrane in CC cells, whereas AKH is more uniformly distributed throughout the CC cytoplasm." This distinction is not clear from the images shown in Figure 3i, and higher-resolution or more definitive localization data are needed to support this statement.

Response: We appreciate the reviewer's careful evaluation of the localization data. In response to this comment, we have revised the manuscript to moderate our previous interpretation that CG14075/Mmt is localized near the plasma membrane whereas AKH is broadly distributed throughout the CC cytoplasm.

We also replaced Fig. 3i with higher-resolution Airyscan images and now describe the data more cautiously, stating that CG14075/Mmt and AKH show a spatially heterogeneous, punctate distribution, and their intensity profiles are not identical. The revised text suggests only that CG14075/Mmt and AKH may differ in their subcellular distribution or organisation within CC cells, without assigning CG14075/Mmt to the plasma membrane or suggesting that the two peptides occupy distinct secretory vesicle populations.

Minor comments:
- Is CG14075/Mmt expressed during the larval stage as well? Do MmtKO mutants develop into adults with normal morphology and viability comparable to wild-type animals?

The authors have mentioned that the loss of CG14075/Mmt does not cause major developmental defects under standard laboratory conditions.

Response: We thank the reviewer for noting that this point has been addressed.

- Figure 1g, add an arrow to point the band in the WB that corresponds to CG14075/Mmt

An arrow now marks the band corresponding to the expected molecular weight of Mmt based on its size.

Response: We thank the reviewer for noting that this point has been addressed.

- Figure 3i- need to add better quality image. Punctate structures are not clearly visible, and they do not match their localization profile described in the text.

Line intensity plot is useful here. However, the images provided are still vague and does not clearly show punctate structures for Mmt and Akh; need to be replaced with clearer and

more zoomed in image.

Response: We have replaced Fig. 3i with higher-resolution images acquired using Airyscan microscopy. The revised images more clearly show spatially heterogeneous, punctate-like CG14075/Mmt and AKH signals within CC cells. We have also retained the line intensity plot to support the comparison of their signal profiles.

- Extended data fig 3h, i is missing- please add them during next submission

Missing image panels added

Response: We thank the reviewer for noting that this point has been addressed.

- Figure 6b, need quantification of CG14075 in Akh-RNAi and AkhR-RNAi animals.

Quantification of CG14075/Mmt levels in Akh-RNAi and AkhR-RNAi animals added in extended data fig 11a

Response: We thank the reviewer for noting that this point has been addressed.

- Figure 6g, need to adjust the model figure as there is not enough evidence for CG14075 to suppress lipolysis and glycogenolysis.

Adjustment has been made to the model figure

Response: We thank the reviewer for noting that this point has been addressed.

- Line 231-232, it would be appropriate to mention that there is a lower level of CG14075 presence during very early stages of starvation and it goes up with energy storage depletion.

Revised accordingly

Response: We thank the reviewer for noting that this point has been revised.

- Line 234, need citation here- “During prolonged starvation, organisms increasingly mobilize amino acids to support gluconeogenesis and ketogenesis”

Citation added

Response: We thank the reviewer for noting that this point has been revised.

- Figure 4a, a vertical line on the x-axis at value 1 to show the changes in amino acid levels more clearly.

New line plots added which clearly shows changes in amino acid levels

Response: We thank the reviewer for noting that this point has been addressed.

Response to reviewers

Ref.: NCOMMS-25-69872B

Manuscript title: “CG14075/Mmt promotes amino acid catabolism and cooperates with AKH during starvation in Drosophila”

We thank the editor and reviewers for their continued evaluation of our manuscript. We are grateful that Reviewer #2 has indicated that we have addressed their concerns and that the revised manuscript is much improved and appropriate for publication.

In this final revision, we have addressed the editorial and production requirements requested by the journal.

Reviewer #2 (Remarks to the Author):

The authors have addressed my concerns. The revised manuscript is much improved and appropriate for publication as far as I am concerned.

Response:

We thank Reviewer #2 for the positive assessment of our revised manuscript. We are grateful that the reviewer considers that we have addressed their concerns and that the revised manuscript is much improved and appropriate for publication.
